# One-pot chemoenzymatic synthesis of trolline and tetrahydroisoquinoline analogues†
†Electronic supplementary information (ESI) available: Experimental details. Preparation of NCS enzymes, reaction details, compound characterization and analytical details. See DOI: 10.1039/c7cc08024g


**DOI:** 10.1039/c7cc08024g

**Published:** 2018-01-18

**Authors:** Jianxiong Zhao, Benjamin R. Lichman, John M. Ward, Helen C. Hailes

**Affiliations:** a Department of Chemistry , University College London , Christopher Ingold Building, 20 Gordon Street , London , WC1H 0AJ , UK . Email: h.c.hailes@ucl.ac.uk; b Department of Biochemical Engineering , University College London , Gower Street , London , WC1E 6BT , UK

## Abstract

Chemoenzymatic reaction cascades can provide access to chiral compounds from low-cost starting materials in one pot. Here we describe one-pot asymmetric routes to tetrahydroisoquinoline alkaloids (THIAs) using the Pictet–Spenglerase norcoclaurine synthase (NCS) followed by a cyclisation, to give alkaloids with two new heterocyclic rings. These reactions operated with a high atom economy to generate THIAs in high yields.

## Abstract 2

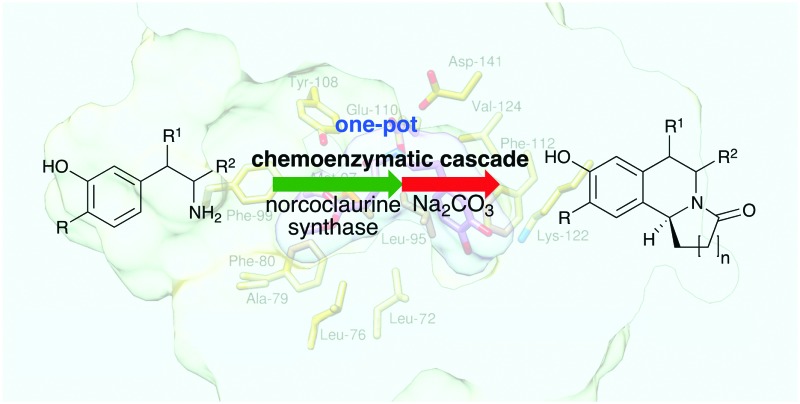
A highly efficient one-pot asymmetric route to tetrahydroisoquinoline alkaloids including the natural product trolline is described.

## 

The use of biocatalytic strategies in synthetic applications continues to offer many advantages compared to traditional chemical approaches due to their sustainability, the use of mild reaction conditions and the high levels of stereocontrol that can be achieved.^1^ Biocatalysts that enable C–C bond formation are particularly useful and norcoclaurine synthases (NCSs) are of significant interest for the synthesis of tetrahydroisoquinoline alkaloids (THIAs).^2^ THIAs are a large group of secondary metabolites with a range of pharmacological activities including the analgesic morphine, anti-hypertensive magnoflorine, and anti-mycobacterial leucoxine.^3^–^5^

In plant biosynthetic pathways, NCS catalyses the Pictet–Spengler reaction (PSR)^6^ between dopamine **1** and 4-hydroxyphenyl acetaldehyde (4-HPAA) to generate (*S*)-norcoclaurine.^7^ Recently, it has been established that a range of aldehydes, particularly substituted phenylacetaldehydes, and some heteroaromatic and aliphatic substrates, can be accepted by recombinant wild-type (WT) *Thalictrum flavum* (*Tf*NCS) and *Coptis japonica* NCS (*Cj*NCS) to generate single-isomer THIAs.^8^–^10^ NCS has been used in chemoenzymatic cascades, with hypochlorite to produce the aldehyde component.^11^,^12^ In addition, NCS enzyme cascades have incorporated an amine oxidase or transaminases to generate aldehydes:^13^,^14^ the latter strategy used a second PSR to produce a tetrahydroprotoberberine.^14^ Engineered microbial pathways to natural opioids incorporating NCS have also been described.^15^,^16^ The reported *Tf*NCS X-ray crystal structure and mechanistic studies,^17^–^19^ together with the aldehyde promiscuity and recent ketone acceptance,^20^ have highlighted the significant potential of using NCSs more widely.

The alkaloid trolline **2**, isolated from the flowers of *Trollius chinensis*, has been reported to be effective against *Staphylococcus aureus* and possess antiviral activity.^21^ There are few reported syntheses, however **3** was used in a Bischler–Napieralski strategy to give **2** in 44% yield (Scheme 1).^22^ Another 3-step approach gave **2** in 73% yield, however the amine starting material was not commercially available.^23^ Multistep asymmetric strategies have been described. One used Jacobsen's catalyst and HCN to establish the C-1 stereochemistry,^24^ while another started with **4** and used the (*R*,*S*_a_)-*N*-pinap chiral catalyst to give (*S*)-**2** in 41% yield (5 steps);^25^ strong bases and toxic solvents were also required.^25^

**Scheme 1:**
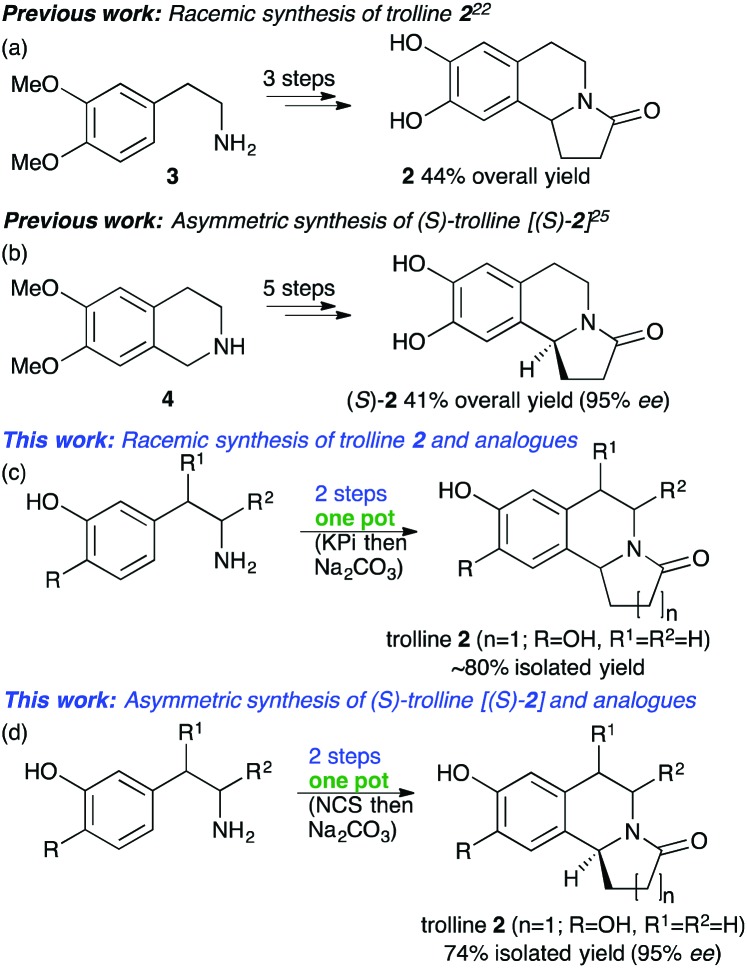
Previous syntheses of trolline (a and b) and the approach used in this work (c and d).

To develop more sustainable rapid routes to these important THIAs, and inspired by the acceptance of non-aromatic aldehydes by NCS, here we describe efficient chemocatalytic cascades to **2** and its analogues in up to 96% yield and >99% ee. One-pot chemoenzymatic cascades offer many advantages, such as avoiding the need for intermediate isolation.^26^–^28^ Two strategies to trolline **2** were adopted: first a biomimetic phosphate-mediated PSR^29^ and subsequent cyclisation to establish the one-pot reaction conditions. Secondly, a one-pot NCS-mediated PSR and then cyclisation to give (*S*)-trolline **2** and its analogues. Initial reactions were carried out using **1** and commercially available **5** under aqueous potassium phosphate (KPi) conditions.^29^ The PSR readily occurred at pH 6 to give a mixture of the linear THIA **6** and, due to spontaneous ring cyclization, **2**. A ratio of amine : aldehyde of 1 : 1.5 gave higher yields. A reaction temperature of 60 °C was optimal; above this, some product decomposition occurred. In addition, as dopamine is oxidatively sensitive, ascorbate was added to avoid side-product formation.^20^ These conditions gave a mixture of linear and cyclised products in a quantitative yield (Table 1). It should be noted that the major *para*-isomer **6** was formed, with some minor *ortho*-isomer (ratio 15 : 1).^12^ The reaction conditions for the cyclisation of linear intermediate **6** were then investigated. Acidic and basic conditions were explored using **6** (*para* : *ortho* 15 : 1) (ESI;† Fig. S1), and sodium carbonate at pH 7.5 gave the highest yields, 89%, of the lactam. A one-pot procedure was then established with **1** and **5** using first step a and then, *via* the addition of sodium carbonate and adjustment of pH, step b, to give **2** in 97% yield (*para* : *ortho* 18 : 1) (Table 1). An extraction and purification protocol was developed^12^,^20^ to avoid the need for chromatographic purification, and *rac*-**2** was isolated in 81% yield (*para* : *ortho* 34 : 1 by HPLC). The one-pot protocol was then applied to aldehyde **7** (*n* = 2). Notably, more linear THIA **8** was generated in the first step while less cyclized product **9** was formed (and some *ortho*-product). The formation of lactam **9** was readily achieved upon the addition of sodium carbonate, and isolated (69% yield, *para* : *ortho* 50 : 1). When aldehyde **10** (*n* = 3) was used, the linear THIA **11** (72% yield, *para* : *ortho* 7 : 1) was formed. No cyclisation to give **12** was observed and **11** was isolated by preparative HPLC.

**Table 1:** One-pot routes to *rac*-**2**, (*S*)-trolline **2** and analogues *via* step a and steps a + b

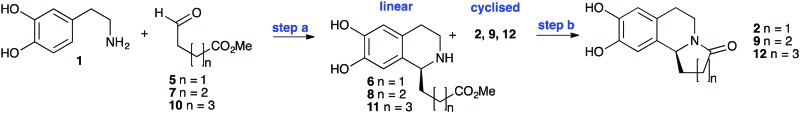						
Aldehyde	Solvent	Step a^*a*^ ^or^^*f*^		Steps a + b^*b*^		
		Linear product yield^*c*^ (*para* : *ortho*)	Cyclised product yield^*c*^ (*para* : *ortho*)	Product yield^*c*^ (*para* : *ortho*)	Isolated product yield (*para* : *ortho*)	Product ee^*h*^
KPi step a^*a*^						
**5**	50% CH_3_CN/KPi	**6** 32% (15 : 1)	**2** 68% (22 : 1)	**2** 97% (18 : 1)	2 81% (34 : 1)^*d*^	na
**7**	50% CH_3_CN/KPi	**8** 75% (8 : 1)	**9** 14% (13 : 1)	**9** 89% (9 : 1)	9 69% (50 : 1)^*e*^	na
**10**	50% CH_3_CN/KPi	**11** 72% (7 : 1)	Only **11**	**11** 72% (7 : 1)	11 26% (7 : 1)^*e*^	na

Enzymatic step a^*f*^						
**5** ^*g*^	10% CH_3_CN/HEPES	**6** 35%	**2** 3%	na	na	nd
**5** ^*g*^	10% DMSO/HEPES	**6** 61%	**2** 7%	na	na	nd
**5**	10% DMSO/HEPES	**6** 71%	**2** 15%	na	na	nd
**5**	1% DMSO/HEPES	**6** 67%	**2** 19%	**2** 75%	**2** 74%^*d*^	95%
**7**	1% DMSO/HEPES	**8** 82%	**9** 15%	**9** 96%	**9** 87%^*d*^	96%
**10**	1% DMSO/HEPES	**11** 92%	only **11**	**11** 92%	**11** 63%^*e*^	>99%

^*a*^ **1** and aldehyde (1 : 1.5) in KPi buffer (0.3 M)/CH_3_CN (1 : 1), 18 h, under Ar, 60 °C, pH 6, and ascorbic acid (1 equiv.).

^*b*^Na_2_CO_3_ (1 M), pH 7.5, and 4 h.

^*c*^HPLC yields: calculated by analytical HPLC.

^*d*^Isolated in high purity by a basic and then acidic extraction procedure using EtOAc and then MeOCO_2_Me.

^*e*^Isolated by preparative HPLC.

^*f*^ **1** and aldehyde (ratio 1 : 1.5) in co-solvent/HEPES buffer (pH 7.5 and 0.1 M), 37 °C, WT-*Tf*NCS (0.1 mg mL^–1^), sodium ascorbate (1 equiv.), and 6 h.

^*g*^As for *f* but **1** : **5** in a ratio of 1.5 : 1, and 3 h reaction.

^*h*^The ees determined by chiral HPLC: for trolline the absolute stereochemistry was confirmed by the optical rotation. na, not applicable. nd, not determined.

The stereoselective approach using NCS was then investigated. Initially, the reaction conditions for the NCS reaction with the novel aldehyde substrates were explored. Using WT-Δ29*Tf*NCS (see the ESI†) and **1** and **5**, the use of acetonitrile and DMSO as co-solvents was investigated as recent work has highlighted several-fold higher product formation with DMSO, perhaps due to enzyme stabilisation effects.^20^ Here DMSO also gave a significantly higher yield (∼2-fold increase) compared to acetonitrile after a 3 h reaction. The extension of the reaction time from 3 h to 6 h, and the use of 1.5 equiv. of aldehyde, enhanced the combined yield of THIAs to 86%: no *ortho*-products were formed. The yield of **2** formed in step a using NCS was lower than that using KPi, reflecting the lower reaction temperatures used. Furthermore, the amount of DMSO co-solvent could be reduced to 1% with little effect on the reaction conversion, and this enhanced the ease of product isolation.

Following the reaction conditions established for step b, (*S*)-**2** was then formed in the one-pot 2-step chemoenzymatic cascade in 75% yield, and 95% ee by chiral HPLC. In a preparative scale reaction, (*S*)-trolline **2** was readily isolated in 74% yield without the use of chromatographic methods using the extraction protocol developed. Remarkably, although aldehyde **5** is structurally quite different from the natural substrate 4-hydroxyphenylacetaldehyde, it was still readily accepted. The extension to aldehyde **7** (*n* = 2) similarly gave lactam **9** in 96% yield (87% isolated yield on a preparative scale) and 96% ee. When using aldehyde **10** (*n* = 3), the linear THIA **11** was formed in 92% yield and in very high optical purity (>99% ee) (Table 1).

The NCS reaction profiles using WT-*Tf*NCS and aldehydes **5**, **7**, and **10** revealed that the bioconversion proceeded more rapidly with the longer chain aldehyde (ESI;† Fig. S2). Computational docking, using the NCS X-ray structure (5NON),^19^ of the imine reaction intermediates when using **1** and **5**, **7**, and **10** was carried out to try and understand this (ESI;† Table S4). Two ‘dopamine-first’ binding modes were observed, both involving the key interaction between the catechol 3-OH and Lys-122 residues.^9^,^18^,^19^ In productive binding modes (Fig. 1A–C), the intermediate occupies a conformation conducive to the subsequent cyclisation step. There were also non-productive binding modes (Fig. 1D–F), with the ester substituent extended into a water channel. The docking experiments suggested that as the aldehyde chain length increases, the productive pose becomes more favourable relative to the non-productive one. For example, docking calculations with **5** (*n* = 1) identified the non-productive pose (Fig. 1D) to be more favourable than the productive pose (Fig. 1A). By comparison, docking with **10** (*n* = 3) indicated the productive pose (Fig. 1C) as more favourable than the non-productive one (Fig. 1F). Such alternative favourable binding arrangements of shorter, less sterically demanding aliphatic aldehydes could lower the enzyme efficiency leading to reduced yields. This has implications in other synthetic applications using less sterically challenging aldehydes, and provides insights for future NCS enzyme engineering.

**Fig. 1:**
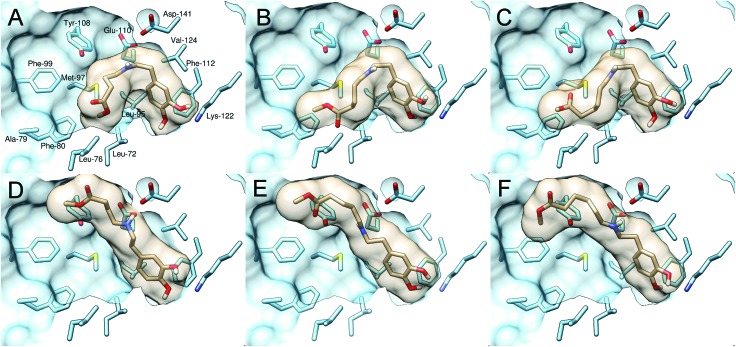
Reaction imine intermediates docked into the NCS X-ray structure (5NON).^19^ Binding modes depicted are the most favoured productive binding modes enabling cyclisation (A–C) or extended non-productive modes (D and E). (A) Productive imine intermediate with aldehyde **5**. (B) Productive imine intermediate with aldehyde **7**. (C) Productive imine intermediate with aldehyde **10**. (D) Non-productive iminium intermediate with aldehyde **5**. (E) Non-productive iminium intermediate with aldehyde **7**. (F) Non-productive iminium intermediate with aldehyde **10**.

The *Cj*NCS and three variants of Δ29*Tf*NCS (A79I, A79F, and F80L) with modified residues at the active site entrance region^9^,^18^,^20^ were also screened against **1** and **5**, **7**, and **10** (ESI;† Fig. S3 and S4). Similar reaction profiles were observed with WT-Δ29*Tf*NCS, although A79F gave slightly higher conversions with **7**: a scale-up reaction (40 mL) generated lactam **9** in 93% isolated yield and >99% ee. The higher ee may reflect steric effects of a more bulky residue on the orientation of intermediates.

The one-pot, 2-step reaction sequence was then extended to dopamine analogues **13–15** using aldehyde **5**. In the KPi-mediated reactions, THIAs were readily formed in up to quantitative yields, again containing predominantly *para*-isomers *rac*-**16–18**, with some *ortho*-product: when using **14**, THIAs were formed as a mixture of isomers (see the ESI†). For WT-*Tf*NCS reactions with **13**, (*S*)-**16** was formed in 51% yield as the only regioisomer and 93% ee (Fig. 2). The bulkier amine metaraminol **14** reacted with **5** in 76% yield, and gave (1*S*)-**17** (at C-1) as the major isomer (ratio 10 : 1) where the minor isomers were a mixture of (1*R*)-**17** (at C-1) and the *ortho*-product (ratio ∼1 : 1). The stereochemistry at C-1 was confirmed by 2D ^1^H NMR spectroscopy. While higher enzymatic stereoselectivities were observed with metaraminol **14** and phenylacetaldehyde,^28^ here a non-aromatic aldehyde, which can give rise to alternative binding arrangements, led to lower selectivities. When using **15**, (*S*)-**18** was formed in 57% yield (87% ee). This is the first time that a fluorinated dopamine analogue has successfully been used with NCS to our knowledge. Small-scale reactions were performed to obtain THIAs **16–18***via* the one-pot reactions and the products were isolated by preparative HPLC.

**Fig. 2:**
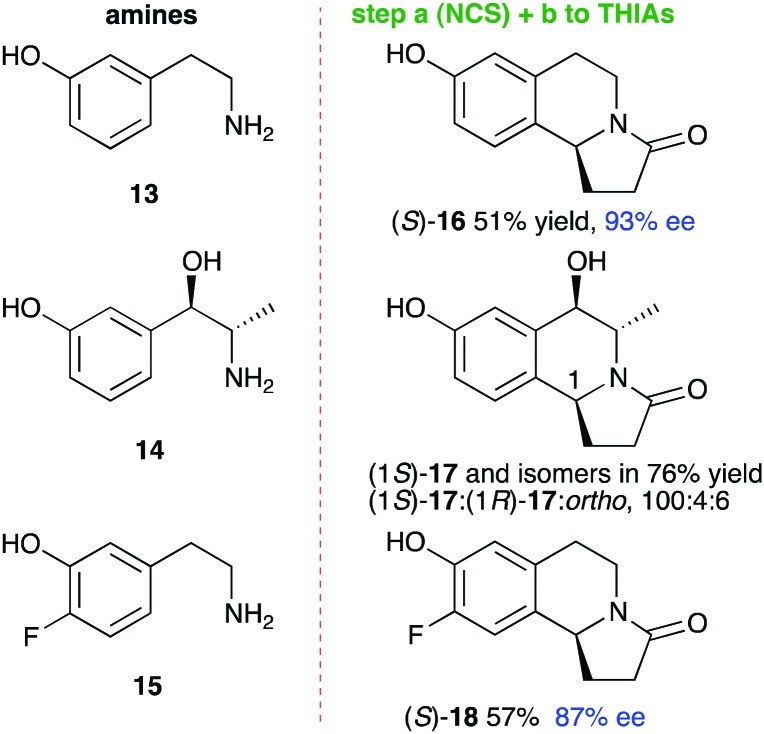
THIAs **16–18** prepared using dopamine analogues and the one-pot 2-step cascade.

Computational modelling of the aminol intermediate with **15** confirmed a similar fit into the active site compared to dopamine (ESI;† Fig. S5). Note that ees were determined by chiral HPLC and the absolute stereochemistry of **16** and **18** was assigned following the established stereoselectivity of the NCS enzymes at C-1 and the correlation to the known stereochemistry of trolline **2**.

Overall, this work highlights the potential of using efficient one-pot chemoenzymatic cascades, not requiring the purification of intermediates, for the rapid stereoselective synthesis of natural and novel alkaloids. It also illustrates the wide substrate tolerance of the NCS enzyme. Furthermore, it demonstrates the application of the sustainable asymmetric catalyst NCS in aqueous media for the synthesis of tricyclic alkaloids.

We gratefully acknowledge UCL (Dean's Prize) and the China Scholarship Council-UCL Joint Research Scholarship for funding to J. Z. and the Wellcome Trust for studentship funding to B. R. L. We also thank K. Karu (UCL Mass Spectrometry Facility) and A. E. Aliev (UCL NMR Facility) of the Department of Chemistry.

## Conflicts of interest

There are no conflicts to declare.

## Supplementary Material

Supplementary information
