# Supplementary material for: One-pot chemoenzymatic synthesis of trolline and tetrahydroisoquinoline analogues
Source: Chem Commun (Camb). 2018 Jan 18;54(11):1323–6. doi: 10.1039/c7cc08024g (PMC5804477; doi:10.1039/c7cc08024g)
Supplement: Supplementary file 1 [file CC-054-C7CC08024G-s001.pdf]

Electronic Supplementary Material (ESI) for ChemComm.
This journal is © The Royal Society of Chemistry 2017

Supporting Information

One-Pot Chemoenzymatic Cascades to Trolline and Tetrahydroisoquinoline Analogues

Jianxiong Zhao, Benjamin R. Lichman, J. M. Ward and Helen C. Hailes*

Table of Contents

1 Supplementary Figures 1-5 Page S3

2 HPLC Methods and Representative HPLC Traces Page S6

3 General Procedures and Characterization Page S11

4 Spectral Data Page S28

5 References Page S48

1 Supplementary Figures 1-5

Supplementary Figure 1 Reaction condition screening for lactam formation using THIA **6** (bases or acids were used at concentrations of 1 M). The final pH of the reaction mixture was 7.5, except for that of the control and the HCl, which was pH 6 and pH 3 respectively. Conversion yields were calculated by HPLC analysis via the consumption of THIA **6** (*para*:*ortho* 15:1).

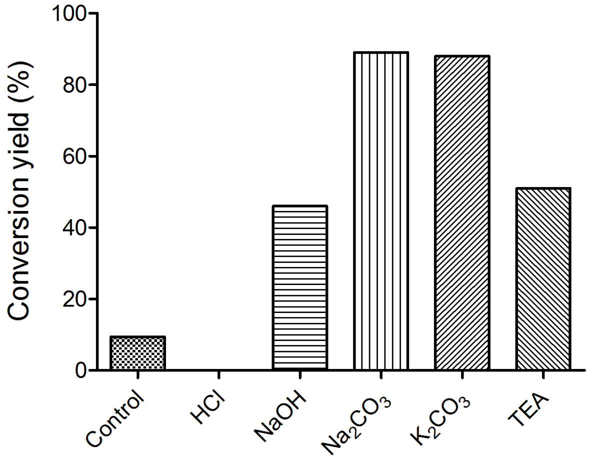

Supplementary Figure 2 Time-dependent depletion of dopamine when using NCS and aldehydes **5**, **7**, **10**. Reactions were carried out on a 1 mL scale and error bars represent the standard errors of three independent experiments at each time point.

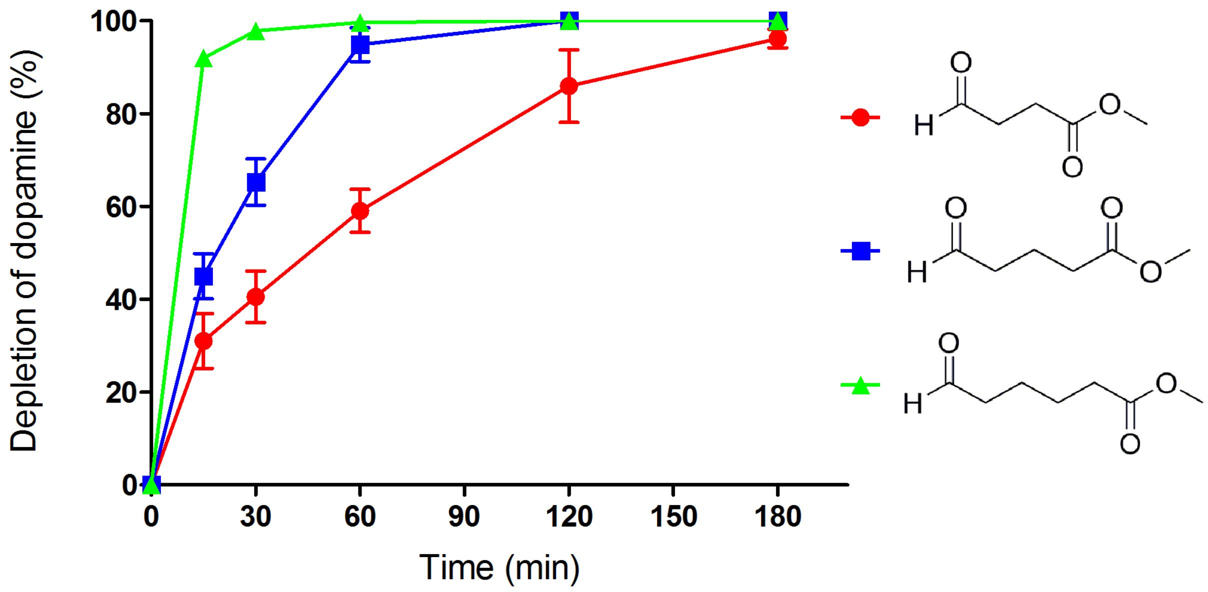

Supplementary Figure 3 Screening of NCS variants[1] in the Pictet-Spengler reaction; Reactions were carried out in triplicate on a 200 μL scale and analysed by HPLC at 280 nm. The HPLC yields were calculated from standard curves of the corresponding products.

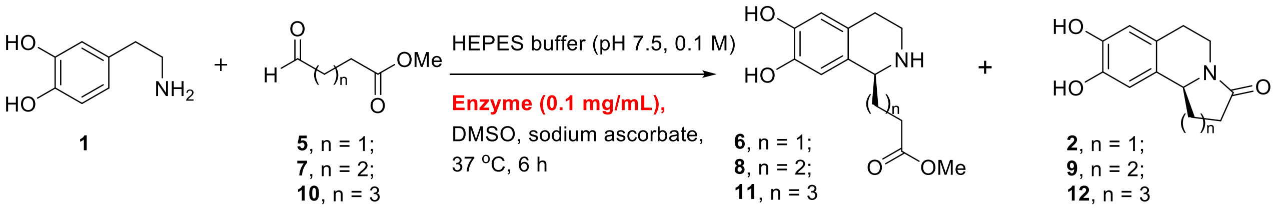

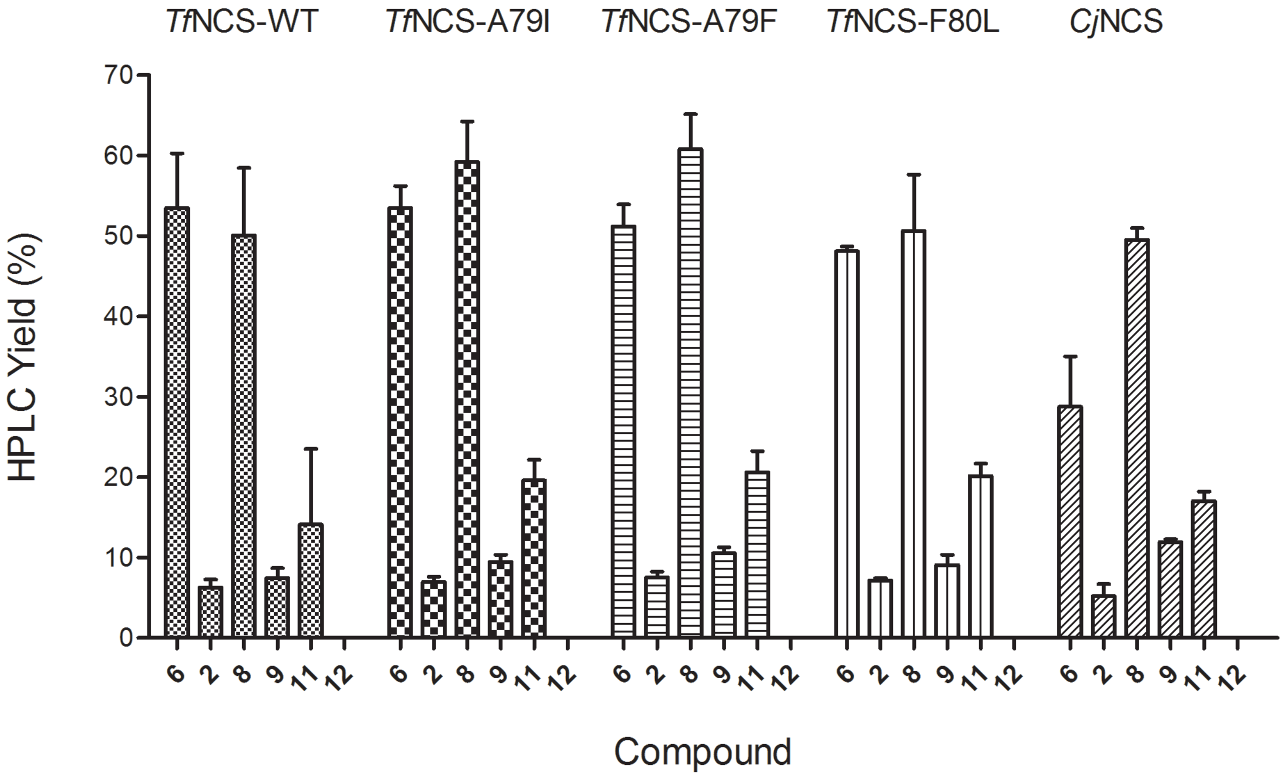

Supplementary Figure 4 NCS protein gels, WT and variants. Figure 4A and 4B: lane 1, protein ladder; lane 2, flow through with *binding buffer A*; lane 3, flow through with *binding buffer B*; lane 4-10, purified protein. Figure 4C: lane 1, protein ladder; lane 2, 5, 8, flow through with *binding buffer A*; lane 3, 6, 9, flow through with *binding buffer B*; lane 4, 7, 10, purified protein.

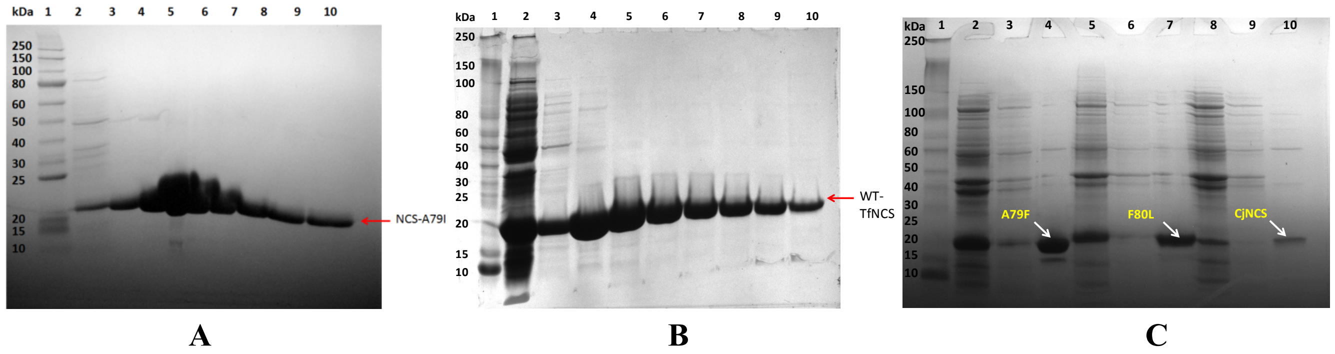

Supplementary Figure 5 a) Aminol reaction intermediate when using **5** and **15** docked into the recently reported NCS X-ray structure 5NON (ΔN33C196*Tf*NCS holo).[2] **b)** The comparable aminol reaction intermediate when using **1** and **5** docked into NCS X-ray structure 5NON.

a)

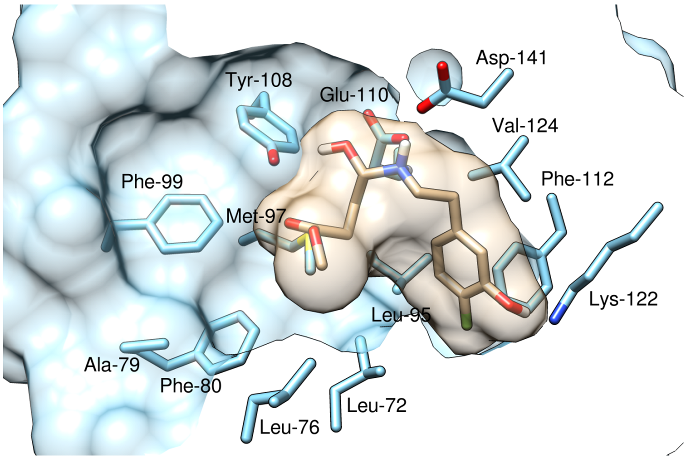

b)

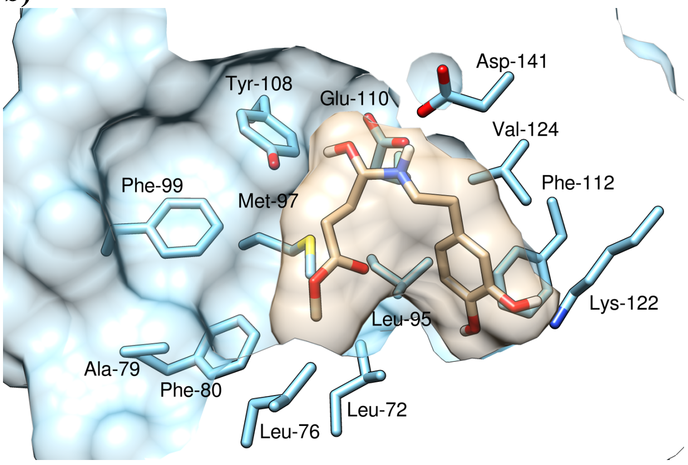

2 HPLC Methods and Representative HPLC Traces

Analytical HPLC method[1] HPLC analysis was carried out using an Agilent 1260 Infinity instrument equiped with ACE 5 C_{18} reverse phase column (150 mm × 4.6 mm). The injection volume was 10 μL, flow rate was 1 mL/min, and UV detection was used at 280 nm (10 min run time). The column temperature was set at 30 °C, eluting with mobile phase A (water with 0.1% TFA) and B (acetonitrile), A/B = 90%/10% at 0-1 min, A/B = 90%/10% to A/B = 30%/70% at 1-6 min, 100% B at 6-6.5 min, A/B = 90%/10% at 6.5-10 min. The retention time of compounds is listed in Table S1.

Table S1

Compound	Retention time (min)	Compound	Retention time (min)
**1** dopamine	2.4	**13**	3.7
**2** trolline	4.9	**14**	3.1
**6**	4.3	**15**	4.5
**8**	4.9	**16**	5.7
**9**	5.3	**17**	5.4
**11**	5.1	**18**	5.9

Semi-preparative HPLC method A. Semi-preparative HPLC was carried out on a C_{18} reverse phase column, Agilent ZORBAX 300SB-C18 (250 mm × 9.4 mm, 5 μm). The flow rate was 2 mL/min with detection at 280 nm. Injection volumes varied depending on the separation achieved (run time 22 min). The column temperature was set at 25 °C, eluting with mobile phase A (water with 0.1% TFA) and B (acetonitrile with 0.1% TFA), A/B = 95%/5% at 0-10 min, A/B = 95%/5% to A/B = 5%/95% at 10-12 min, A/B = 5%/95% at 12-15 min, A/B = 5%/95% to A/B = 95%/5% at 15-16 min, A/B = 95%/5% at 16-22 min.

Semi-preparative HPLC method B. Semi-preparative HPLC was carried out on a C_{18} reverse phase column, Agilent ZORBAX 300SB-C18 (250 mm × 9.4 mm, 5 μm). The flow rate was 2 mL/min with detection at 280 nm. Injection volumes varied depending on the separation achieved. The run time was 25 min. The column temperature was set at 25 °C, eluting with mobile phase A (water with 0.1% TFA) and B (acetonitrile with 0.1% TFA), A/B = 95%/5% at 0-6 min, A/B = 95%/5% to A/B = 55%/45% at 6-10 min, A/B = 55%/45% at 10-15 min, A/B = 55%/45% to A/B = 10%/90% at 15-16 min, A/B = 10%/90% at 16-18 min, A/B = 10%/90% to A/B = 95%/5% at 18-20 min, A/B = 95%/5% at 20-25 min.

Semi-preparative HPLC method C. Semi-preparative HPLC was carried out on a C_{18} reverse phase column, Agilent ZORBAX 300SB-C18 (250 mm × 9.4 mm, 5 μm). The flow rate was 3 mL/min with detection at 280 nm. Injection volumes varied depending on the separation achieved. The run time was

16 min. The column temperature was set at 25 ^{o}C, eluting with mobile phase A (water with 0.1% TFA) and B (acetonitrile with 0.1% TFA), A/B = 95%/5% at 0-1 min, A/B = 95%/5% to A/B = 5%/95% at 1-11 min, A/B = 5%/95% at 11-13 min, A/B = 95%/5% 13-16 min.

Chiral HPLC method

Method A: Machine: Agilent HPLC; Column: ASTEC CHIROBIOTIC^{TM} T column (25 cm × 4.6 mm, 5 μm); Flow rate: 1 mL/min; Temperature: 25 ^{o}C; Injection: 5 μL; Wavelength: 280 nm; Mobile phase: methanol (0.2% AcOH, 0.1% TEA): H_2O = 97: 3. Run time: 30 min.

Method B: Machine: Agilent HPLC; Column: ASTEC CHIROBIOTIC^{TM} T column (25 cm × 4.6 mm, 5 μm); Flow rate: 1 mL/min; Temperature: 30 ^{o}C; Injection: 5 μL; Wavelength: 210 nm; Mobile phase: Isopropanol - NH_4OAc (5 mM, pH = 4.1) = 20:80. Run time: 10 min.

Method C: Machine: Dionex HPLC; Column: ASTEC CHIROBIOTIC^{TM} T column (25 cm × 4.6 mm, 5 μm); Flow rate: 1 mL/min; Temperature: 30 ^{o}C; Injection: 5 μL; Wavelength: 254 nm; Mobile phase: Isopropanol - NH_4OAc (5 mM, pH = 4.9) = 15:85. Run time: 20 min.

HPLC standard curves Compounds were accurately weighed and dissolved in acetonitrile-water (1:1) or methanol-water (1:1), based on their solubility, to make a stock solution of 5.0 mM. The stock solution was diluted with water to 2.0 mM, 1.0 mM, 0.50 mM, 0.20 mM and 0.10 mM. All the solutions with known concentrations were analysed in the HPLC following the *analytical HPLC method*. HPLC peak area S (mAU^2) and compound concentration C (mM) were recorded and fitted to a straight line using EXCEL software, giving the standard curve as the format of $S = aC + b$.

Representative HPLC Traces

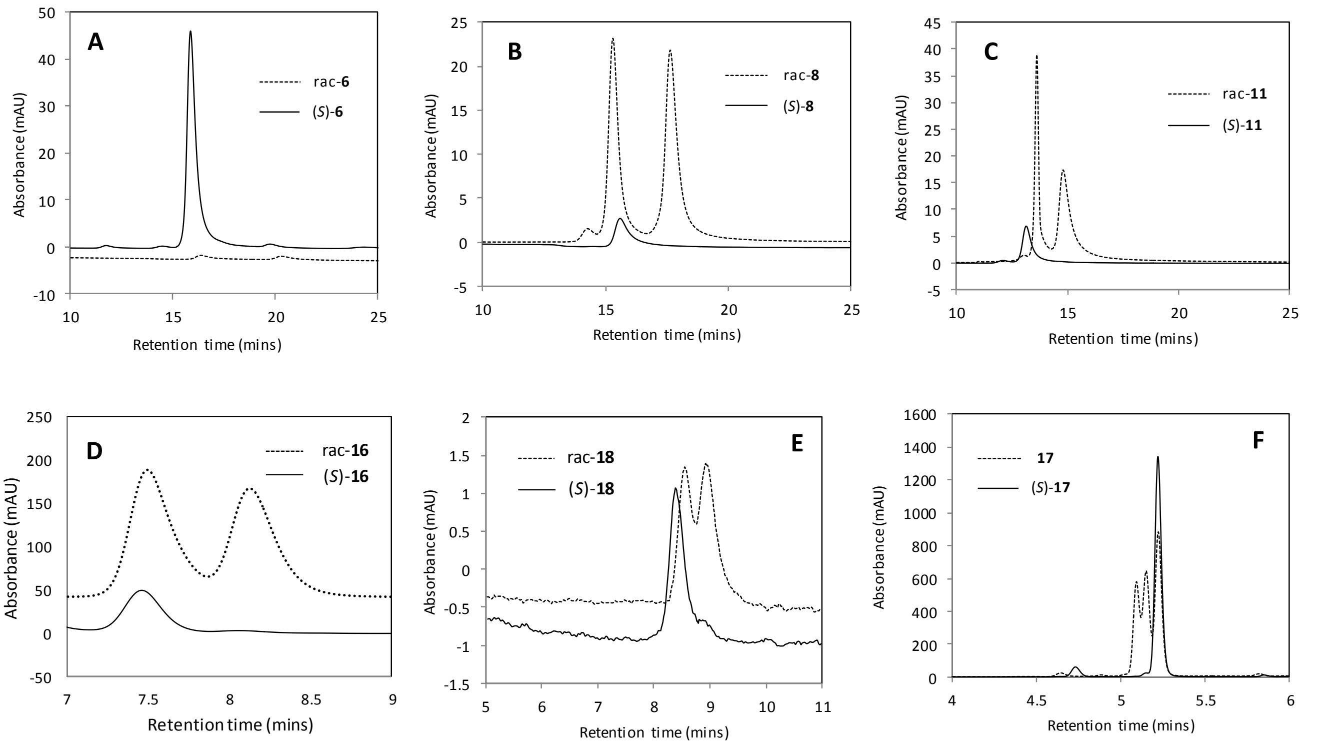

Supplementary Figure 6 Chiral HPLC chromatogram of chemoenzymatic cascades to trolline and analogues.

Table S2

Compound	Method	Retention time (min), (*S*)-C-1	Retention time (min), (*R*)-C-1	*ee*
**6**	*Chiral HPLC Method A*	16.4	20.3	95%
**8**	*Chiral HPLC Method A*	15.3	17.6	96%
**11**	*Chiral HPLC Method A*	13.6	14.8	>99.5%
**16**	*Chiral HPLC Method B*	7.5	8.1	93%
**18**	*Chiral HPLC Method C*	8.6	8.9	87%
**17**	*Analytical HPLC method*	(*S*)-C-1, 5.22; (*R*)-C-1, 5.15; *ortho*, 5.09		--

Compound 2

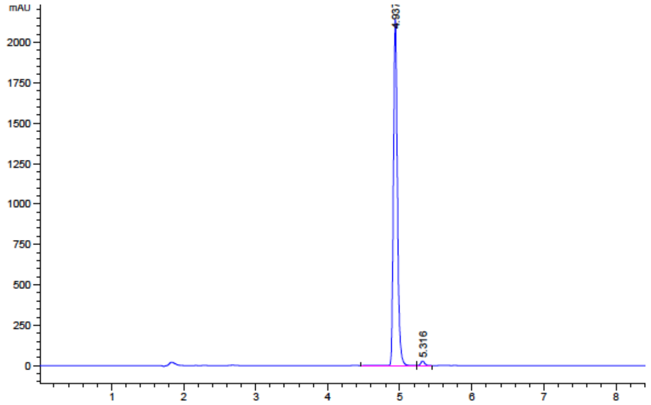

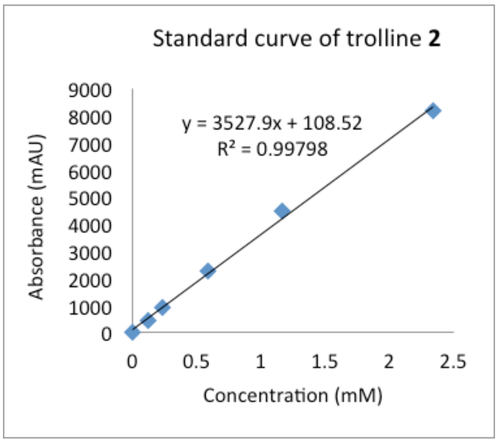

Compound 6

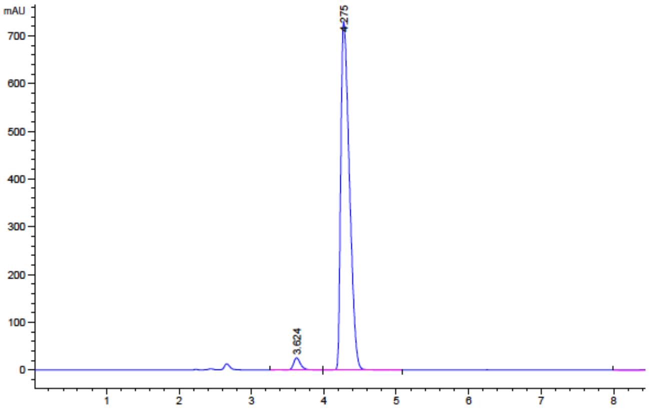

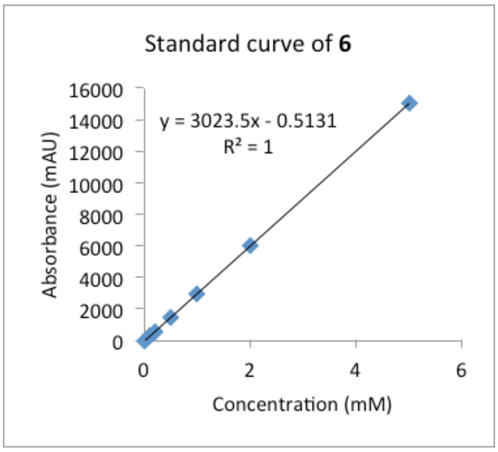

Compound 8

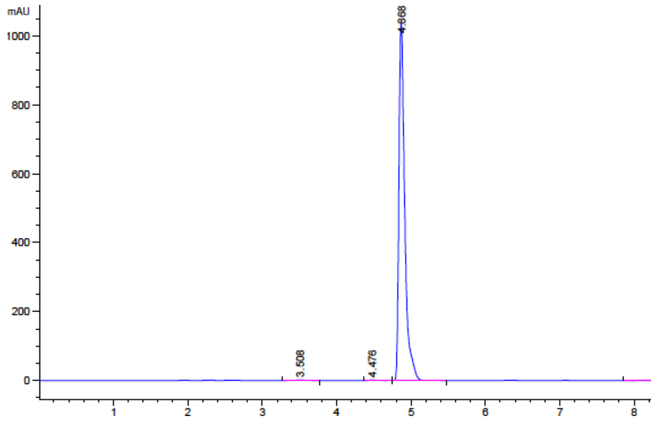

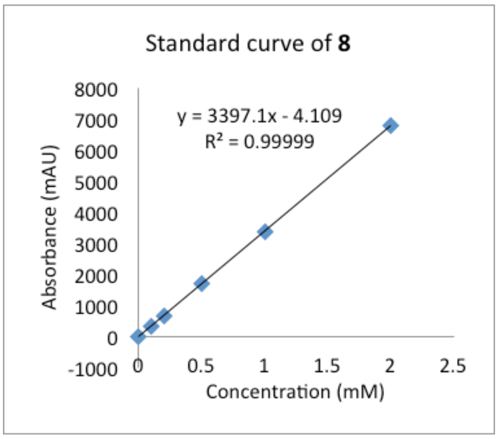

Compound 9

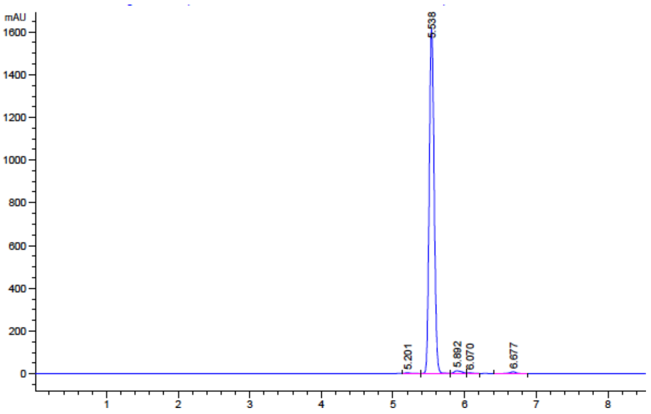

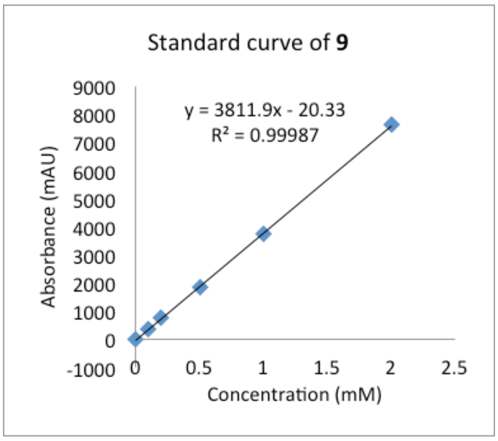

Compound 11

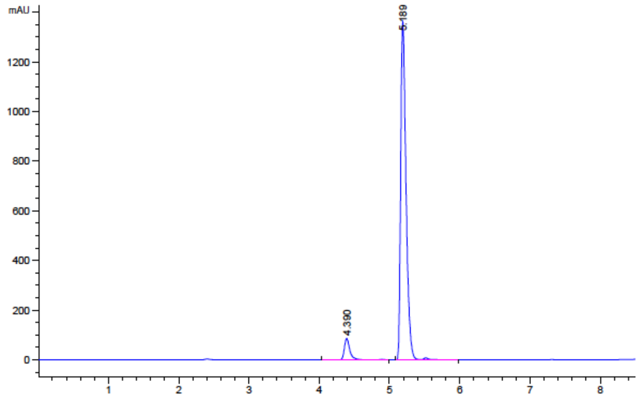

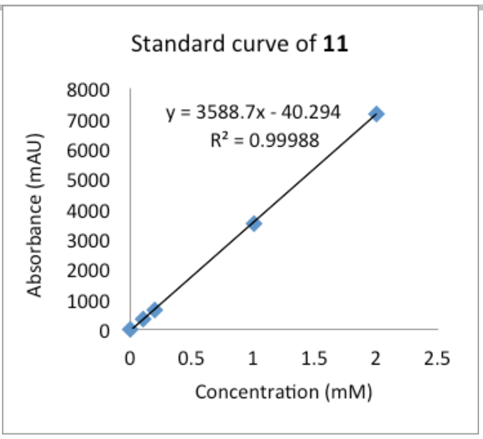

Compound 16

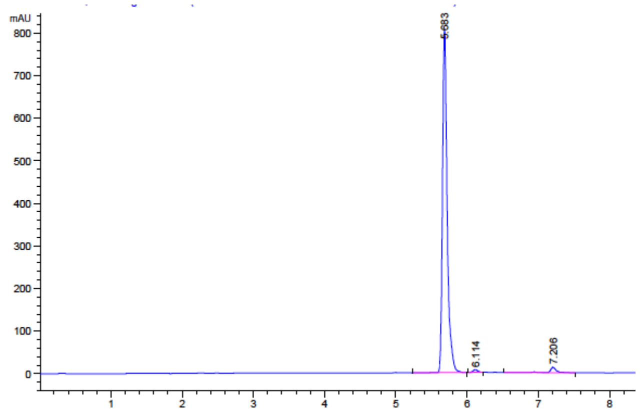

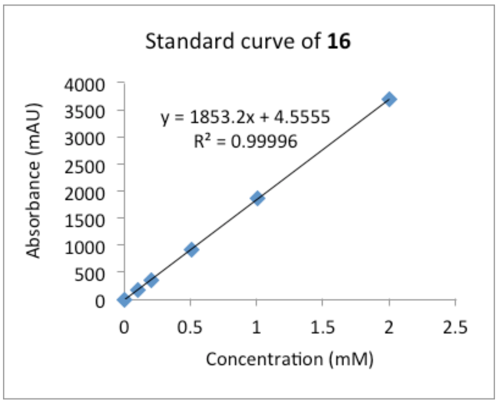

Compound 17

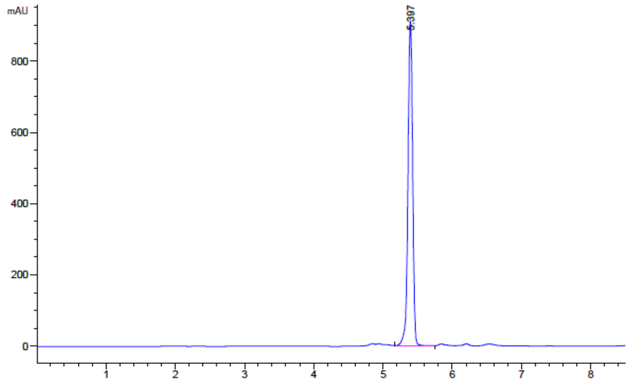

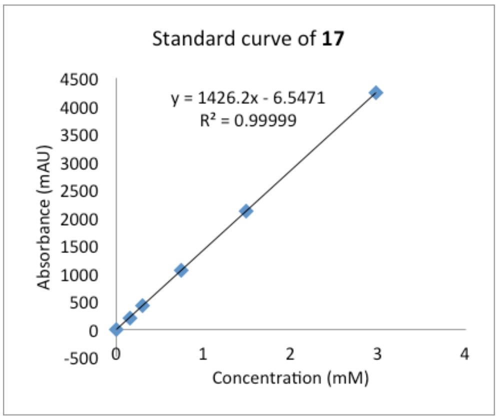

Compound 18

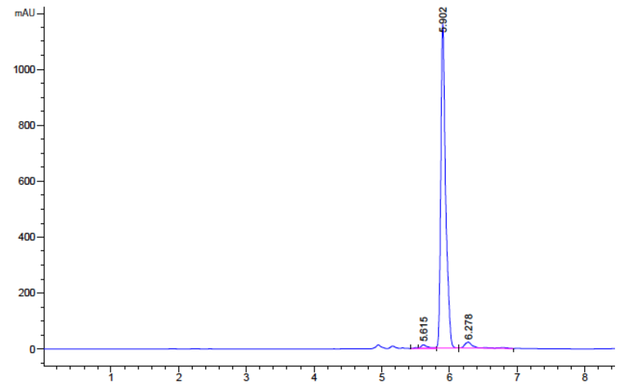

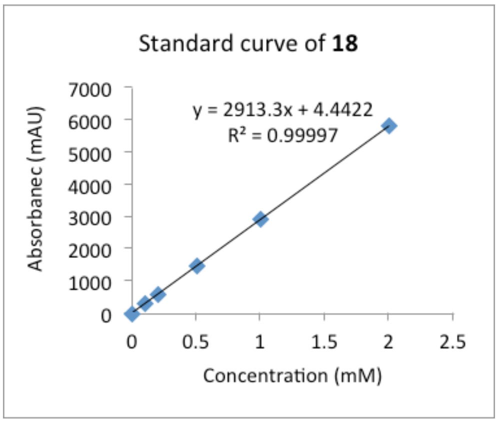

Supplementary Figure 7 HPLC chromatograms (2.0 mM for compounds **2**, **6**, **8**, **9**, **11**, **16**, **18** and 3.0 mM for compound **17**) and standard curves of the reference compounds.

3 General Procedures and Compound Characterization

Protein Expression Glycerol stocks of *E. coli* BL21 (DE3) cells transformed with plasmids (His-tag NCS or variants, with 29 amino acids deleted at the C-terminus) were incubated in terrific broth (TB, Fluka Analytical company) medium (20 mL) containing 50 µg/mL of kanamycin for 16 h at 37 °C, 250 rpm. The starter culture (4 mL) was then incubated with TB medium (100 mL) containing 50 µg/mL of kanamycin for 1 h at 37 °C, 250 rpm until OD_{600} = 1.0 detected by the spectrophotometer (JENWAY 7315). Isopropyl β-D-1-thiogalactopyranoside (IPTG, final concentration was 500 µM) was added and the mixture was incubated for 5 h at 25 °C, 250 rpm. The expression cultures were centrifuged at 12000 rpm for 10 min at 4 °C and the supernatant was removed. Cell pellets were collected and stored at -20 °C.[3]

Protein Purification Cell Pellets were resuspended in 10% v/v original culture volume of Bugbuster (Novagen Bugbuster® 10X Protein Extraction Reagent, diluted to 1X prior to usage). The mixture was centrifuged for 30 min at 4 °C, 12000 rpm. The supernatant was then filtered using a 0.22 µm cellulose acetate syringe filter (Millex®-GP) and passed through the Ni-NTA column (a PD-10 column filled with 2.3 mL of Nickel-NTA resin Agarose, Invitrogen) previously equilibrated with *binding buffer A* (100 mM HEPES, 100 mM NaCl, 20 mM imidazole, pH 7.5). The column was washed with *binding buffer B* (100 mM HEPES, 100 mM NaCl, 40 mM imidazole, pH 7.5) and eluted with *elution buffer* (100 mM HEPES, 100 mM NaCl, 500 mM imidazole, pH 7.5) until no protein was left on the column (checked by Bio-Rad Bradford 1x Dye Reagent). The eluent containing pure protein was exchanged into *HEPES buffer* (100 mM HEPES, pH 7.5) using a PD-10 desalting column (GE Healthcare Sephadex™ G-25 M). Protein purity was tested by SDS-PAGE (Gel results referred to supplementary Figure 4) and protein concentration was tested with a Thermo Scientific NANODROP 2000c Spectrophotometer at 280 nm. Glycerol (final concentraion 10% v/v) was added to the pure protein solution and stored at -80 °C.[3]

Protein Purity Test Protein sample was analysed by SDS-12% polyacrylamide gel electrophoresis (SDS-PAGE) on Mini-Protein Precast Gels (Bio-Rad company). Briefly, the protein composition was prepared by mixing 10 µL loading buffer, 10 µL DTT and 20 µL testing sample. The prepared mixture as well as a Ladder protein marker (10-25 kDa, New England Biolabs) were boiled at 95 °C for 15 min and spun at 13000 rpm for 10 min. The supernatants were loaded to the gels and run (200 V for 40 min). The gel was then washed with distilled water and treated with instant blue overnight. A ProteinSimple camera and AlphaImager MiNi software were used to take a picture of the gel.

Chemical Reagent Flash silica chromatography was carried out using Geduran® Si 60 (40-63 µm). Thin layer chromatography (TLC) used aluminium backed silica gel plates from Merck Keiselgel and were visualised using ultra-violet light (254 nm). The solvents and chemicals were purchased from Sigma Aldrich, Alfa Aesar, Santa Cruz Biotechnology and were used as supplied unless indicated otherwise.

Chemical Characterization Melting points were tested using Stuart (SMP11) and DigiMelt MPA161 melting point machines. The value of $[\alpha]_D$ was recorded using PerkinElmer Model 343 Polarimeter machine in the solvent indicated. ^{1}H and ^{13}C NMR spectra were recorded on Bruker Avance III 600, Bruker Avance III 400, Bruker Avance 300 spectrometer instruments at the field indicated. Chemical

shifts (in ppm) were determined relative to tetramethylsilane (TMS) and referenced to residual protonated solvent. Coupling constants (*J*) were measured in Hertz (Hz) and multiplets of ^{1}H NMR spectroscopy couplings are shown as singlet (s), doublet (d), triplet (t) etc. Two dimentional NMR spectra ^{1}H-^{1}H COSY, ^{1}H-^{13}C HSQC, ^{1}H-^{13}C HMBC and ^{1}H-^{1}H NOESY were used for compound identification when required. Mass spectra were aquired using a Finnigan MAT900 XE Mass spectrometer for Chemical Ionization (CI) / Electron Ionization (EI) and a Waters Premier XE QTOF Mass Spectrometer for ESI.

Small-scale (1 mL) KPi and NCS reactions

For phosphate reactions, a mixture of KPi buffer (0.3 M, pH 6, 0.5 mL) and acetonitrile (0.5 mL) was added to give a final concentration of dopamine **1** (10 mM), methyl 4-oxobutanoate **5** (15 mM) and ascorbic acid (10 mM) in a 1.5 mL capped microcentrifuge tube. The reactions were flushed with argon, sealed and shaken for 4 h at 60 ^{o}C, 500 rpm. Reactions were monitored by taking 100 μL samples, diluting with water (450 μL) and analysed by HPLC following the *analytical HPLC method*. Then 800 μL of the reaction mixtures were treated with 200 μL sodium carbonate solution (1 M) and reactions were shaken for 1 h at 60 ^{o}C, 500 rpm. Similar procedures were used with methyl 5-oxopentanoate **7** and methyl 6-oxohexanoate **10**. For amines **13**, **14** and **15** reacted with **5**, the first reactions (step a) were performed for 18 h and the second reactions (cyclisations) were for 2 h (**13** and **15**) and 4 h (**14**). HPLC yields were calculated based on standard curves established with pure compounds (see Table 1 in the main text).

For enzyme reactions, HEPES buffer (100 mM HEPES, pH 7.5) was added with a final concentration of DMSO (1% v/v), dopamine **1** (10 mM), methyl 4-oxobutanoate **5** (15 mM), sodium ascorbate (10 mM) and Δ29*Tf*NCS (wild type, 0.1 mg/mL) in a 1.5 mL capped microcentrifuge tube. The reactions were flushed with argon, sealed and shaken for 3 h at 37 ^{o}C, 500 rpm. Reactions were monitored by taking 100 μL sample diluting with water/HCl (10 μL HCl + 440 μL water) and analysed by HPLC following the *analytical HPLC method*. Then 800 μL of the reaction mixtures were treated with 80 μL HCl (1 M) and 120 μL sodium carbonate solution (1 M) and shaken for 2 h at 60 ^{o}C, 500 rpm. Similar procedures were used with methyl 5-oxopentanoate **7** and methyl 6-oxohexanoate **10**. For amines **13, 14** and **15** reacted with **5**, the first reactions (step a, 0.5 mg/mL WT-Δ29*Tf*NCS) were 6 h (**13** and **15**) or 18 h (**14**) and the second reactions (cyclisations) were for 2 h (**13** and **15**) and 4 h (**14**). HPLC yields were calculated based on standard curves as above (see Table 1 in the main text).

Screening of reaction conditions for second-step (cyclization). Dopamine hydrochloride (76 mg, 0.4 mmol), methyl 4-oxobutanoate **5** (64 μL, 0.6 mmol) and ascorbic acid (36 mg, 0.2 mmol) were dissolved in KPi buffer (0.3 M, pH 6, 20 mL) and acetonitrile (20 mL). The mixture was stirred for 18 h at 60 ^{o}C under argon. The reaction mixtures were then seperated intro 6 fractions, among which 5 fractions (each 5 mL) were treated with NaOH (0.55 mL, 1 M), Na_2CO_3 (0.48 mL, 1 M), K_2CO_3 (0.45 mL, 1 M), triethylamine (0.08 mL) to adjust the pH to 7.5 and 1 M HCl (0.47 mL) to adjust the pH to 3.0. The fraction remaining was used as a blank control. All the reactions were stirred for 4 h at 60 ^{o}C under argon and analysed by HPLC following the *analytical HPLC method*. Conversion yields were calculated based on depletion of the linear intermediate **6** and results were shown in Supplementary Figure 1.

NCS variant screening Reactions were carried out in a 96-well plate on a 200 μL scale (in triplicate). Stock solutions were prepared with HEPES buffer (100 mM, pH 7.5). *Stock Solution A*: dopamine and sodium ascorbate (100 mM each, 2 mL); *Stock Solution B*: aldehydes (**5**, **7**, **10**) and DMSO (150 mM and 10% v/v, 2 mL). *Stock Solution A* (40 μL) and *Stock Solution B* (40 μL) were added with enzymes (∆29*Tf*NCS-WT, ∆29*Tf*NCS-A79I, ∆29*Tf*NCS-A79F, ∆29*Tf*NCS-F80L, *Cj*NCS) and HEPES buffer (100 mM, pH 7.5) to make the final volume of 200 μL. The recipe for each well was shown as below in Table S2. The plates were sealed and shaked for 6 h at 37 ^{o}C, 250 rpm. The reactions were quenched by adding 20 μL HCl (1 M) to each well. Then 100 μL of reaction mixture was collected from each well, diluted with H_2O (500 μL) and analysed by HPLC following the *analytical HPLC method*. Average HPLC yields (linear and cyclized products) and standard errors were calculated (Supplementary Figure 3).

Table S3 Final concentration in each well (total volume 200 μL)

Component	Concentration
Dopamine	10 mM
Aldehyde	15 mM
Sodium Ascorbate	10 mM
DMSO	10% v/v
Enzyme	0.1 mg/mL

Time-dependent study of NCS reactions Dopamine hydrochloride (7.6 mg, 0.04 mmol, 10 mM), sodium ascorbate (8.0 mg, 0.04 mmol, 10 mM), DMSO (40 μL, 1% v/v) and WT-∆29*Tf*NCS (final concentration 0.1 mg/mL) were added to HEPES buffer (pH 7.5, 100 mM) to make a final volume of 4 mL. The mixture was seperated into 4 fractions (equal volume 1 mL) into capped microcentrifuge tubes. To each fraction was added freshly purified methyl 4-oxobutanoate **5** (95%, 1.8 μL, 0.06 mmol) except one control reaction. Similar procedures were applied with methyl 5-oxopentanoate **7** and methyl 6-oxohexanoate **10**. All mixtures were shaken under argon for up to 3 h at 37 ^{o}C, 500 rpm. At each time point 15 min, 30 min, 60 min, 120 min and 180 min, 100 μL of the reaction mixture was collected, quenched by aq. HCl (10 μL, 1 M) and diluted with H_2O (440 μL). Collected samples were analysed by HPLC following the *analytical HPLC method*. The depletion percentage of dopamine at each time point was calculated following the formula: $(S_{control}-S_{experiment})/ S_{control}\times100\%$, S was the HPLC peak area of dopamine. Average depletion of dopamine of three independent reactions and standard errors were calculated and displayed in Supplementary Figure 2. Note that dopamine depletion was used to monitor the reactions to reduce complexity of the assay due to linear and cyclic products being formed.

Computational docking Computational docking calculations were conducted using AutoDock Vina[4], implemented through UCSF Chimera and the Opal web service. Ligand structures were prepared and energy minimised on the molview.org server prior to docking. PDB structure 5NON was prepared by converting selenomethionines to methionines, removing ligands and adding hydrogens as previously.[5] The docking prameters were: center_x = -16.99; center_y = -5.81; center_z = 15.42; size_x = 16.56; size_y = 14.22; size_z = 22.15; energy_range = 3; exhaustiveness = 8; num_modes = 10. Ten binding modes were predicted for each ligand, and only top ranked modes which showed interactions in line with the dopamine-first mechanism[2,5] were analysed.

Table S4 Binding energies in kcal mol^{-1} for imine intermediates (between **1** and **5**, **7**, **10**)

	Imine (**1** and **5**)		Imine (**1** and **7**)		Imine (**1** and **10**)	
Rank	Energy	Pose	Energy	Pose	Energy	Pose
1	-6.6		-6.7	2	-6.2	
2	-6.4	2	-6.4	1	-6.1	1
3	-6.3		-6.4		-6.1	
4	-6.1		-6.3		-6.1	
5	-6.0		-6.3		-6.0	
6	-6.0		-6.2		-6.0	2
7	-6.0	1	-6.2		-5.9	
8	-5.9		-6.0		-5.8	
9	-5.8		-5.9		-5.8	
10	-5.8		-5.8		-5.7	

Pose 1: expected productive binding mode of binding enabling cyclisation in iminium.
Pose 2: extended pose non-productive for cyclisation

Methyl 5-oxopentanoate (7)[6]

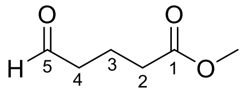

To a solution of δ-valerolactone (10.0 g, 99.9 mmol) in methanol (30 mL), concentrated sulfuric acid (0.4 mL) was added and the mixture was stirred for 2 h at 65 °C. It was then cooled to 0 °C and H_2O (30 mL) was added. The solution was adjusted to pH 8.0 by adding aq. Na_2CO_3 (1 M). The mixture was extracted with ether (60 mL, then 2 × 30 mL), washed with brine (2 × 30 mL), dried (Na_2SO_4) and concentrated under vacuum to give the alcohol as a colourless oil (5.14 g, 39%), which was used directly in the next step. ^{1}H NMR (400 MHz; $CDCl_3$) δ 3.66 (s, 3H, OMe), 3.62 (t, *J* = 6.1 Hz, 2H, CH_2OH), 2.34 (t, *J* = 7.2 Hz, 2H, CH_2CO_2Me), 1.74–1.65 (m, 2H, CH_2), 1.63–1.54 (m, 2H, CH_2). To a solution of the alcohol (1.29 g, 9.76 mmol) in dichloromethane (50 mL) was added pyridinium chlorochromate (3.31 g, 15.4 mmol) and silica (6.50 g). The mixture was stirred for 2 h at room temperature, then filtered through a sintered funnel containing SiO_2 (1 cm height). The filtrate was concentrated under the vacuum and purified by SiO_2 column chromatography (petroleum ether and ethyl acetate, 5:1) to give aldehyde **7**[6] as a colourless oil (295 mg, 23%). R_f = 0.27 (petroleum ether and ethyl acetate, 5:1); ^{1}H NMR (600 MHz; $CDCl_3$) δ 9.68 (t, *J* = 1.2 Hz, 1H, CHO), 3.58 (s, 3H, OMe), 2.45 (td, *J* = 7.2, 1.2 Hz, 2H, 4-H), 2.29 (t, *J* = 7.2 Hz, 2H, 2-H), 1.87 (quint, *J* = 7.2 Hz, 2H, 3-H); ^{13}C NMR (151 MHz; $CDCl_3$) δ 201.6 (CHO), 173.4 (CO_2Me), 51.6 (OMe), 42.9, 32.9, 17.4 (C-3); *m/z* [CI] 148 ($[MH+NH_3]^+$, 48%), 131 ($[MH]^+$, 9), 130 ($[M]^+$, 100).

Methyl 6-oxohexanoate (10)[7]

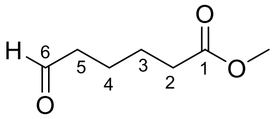

To a solution of ε-caprolactone (10.0 g, 87.6 mmol) and methanol (30 mL), concentrated sulfuric acid (0.4 mL) was added, and the mixture was heated at reflux for 2 h. It was then cooled to room temperature, and water (30 mL) was added. The solution was adjusted to pH 8.0 by adding 1 M aq.

Na_2CO_3. Then the mixture was extracted with ether (3 × 30 mL), washed with brine (2 × 20 mL), dried (Na_2SO_4) and concentrated under vacuum to give the alcohol as a colourless oil (10.0 g, 78%), which was used directly in the next step. ^{1}H NMR (400 MHz; $CDCl_3$) δ 3.60 (s, 3H, OMe), 3.56 (t, J = 7.0 Hz, 2H, $C\underline{H}_2OH$), 2.26 (t, J = 7.2 Hz, 2H, $C\underline{H}_2CO_2Me$), 1.60 (quint, J = 7.0 Hz, 2H, $C\underline{H}_2CH_2OH$), 1.51 (quint, J = 7.2 Hz, 2H, $C\underline{H}_2CH_2CO_2Me$), 1.33 (m, 2H, CH_2). To a solution of the alcohol (1.46 g, 9.99 mmol) in dichloromethane (50 mL) was added pyridinium chlorochromate (3.31 g, 15.4 mmol) and silica (6.5 g). The mixture was stirred for 2 h at room temperature, then filtered through a sintered funnel containing SiO_2 (1 cm height). The filtrate was concentrated under the vacuum and purified by SiO_2 column chromatography (petroleum ether and ethyl acetate, 5:1) to give aldehyde **10**[7] as a colourless oil (637 mg, 44%). R_f = 0.47 (petroleum ether and ethyl acetate, 5:1); ^{1}H NMR (600 MHz; $CDCl_3$) δ 9.66 (t, J = 1.5 Hz, 1H, CHO), 3.56 (s, 3H, OMe), 2.38–2.36 (m, 2H, 5-H), 2.25–2.23 (m, 2H, 2-H), 1.57–1.54 (m, 4H, 3-H and 4-H); ^{13}C NMR (151 MHz; $CDCl_3$) δ 202.2 (CHO), 173.7 ($\underline{C}O_2CH_3$), 51.5 ($CO_2\underline{C}H_3$), 43.5, 33.7, 24.3, 21.5; *m/z* [ES+] 145 ([MH]$^+$, 5%), 144 ([M]$^+$, 20), 143 (100).

Trolline (2)[8]

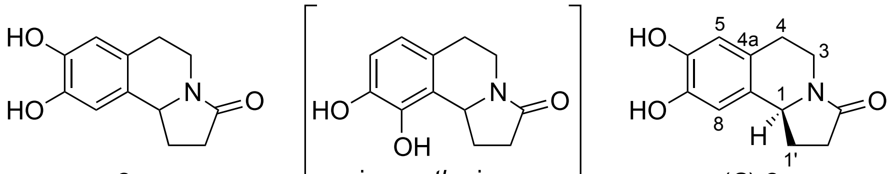

2 minor *ortho*-isomer (*S*)-**2**

Synthesis of (±)-trolline **2**.

(i) Small-scale (1 mL) KPi reaction.

The procedure described on pS11 was used and reaction yields determined by HPLC. Trolline **2** was formed in 97% yield in a *para*:*ortho* isomeric ratio of 18:1 (see Supplementary Figure 8B).

(ii) Preparative scale reaction for product isolation and characterisation.

To a solution of dopamine hydrochloride (76 mg, 0.40 mmol), ascorbic acid (70 mg, 0.40 mmol), KPi buffer (20 mL, pH 6, 0.3 M) and CH_3CN (20 mL) was added methyl 4-oxobutanoate **5** (64 μL, 0.60 mmol). The mixture was heated at 60 °C and stirred for 18 h under argon. The pH of the solution was then adjusted to 7.5 by adding aq. Na_2CO_3 (1 M) and stirred for another 4 h at 60 °C under argon. The product was extracted with ethyl acetate (3 × 30 mL), the organic layer dried (Na_2SO_4) and evaporated to give a yellow solid. The residue was re-suspended in aq. HCl (10 mL, 1 M) and dimethyl carbonate (DMC) (10 mL), and the aqueous layer was washed with DMC (3 × 5 mL). The DMC fractions were combined and washed with aq. HCl (3 × 5 mL, 1 M). The aqueous phase was co-evaporated with methanol at 55 °C to obtain a white solid, (±)-trolline **2** (71 mg, 81%) in a *para*:*ortho* isomeric ratio of 34:1. The characterization data is given below for the (*S*)-isomer.

*Synthesis of (-)-trolline (S)-***2**.

(i) Small-scale (1 mL) NCS reaction.

The procedure described on pS11 was used and reaction yield determined by HPLC. Trolline (*S*)-**2** was was formed in 75% yield (see Supplementary Figure 8D) and no minor isomeric product was observed. The *ee* was 95% by chiral HPLC (of intermediate (*S*)-**6**: see Supplementary Figure 6A and Table S2).

(ii) Preparative scale reaction for product isolation and characterisation.

Dopamine hydrochloride (76 mg, 0.40 mmol) and sodium ascorbate (80 mg, 0.40 mmol) were added to HEPES buffer (38.9 mL, pH 7.5, 100 mM). Methyl 4-oxobutanoate **5** (64 µL, 0.60 mmol) in DMSO (0.4 mL) was added, followed by WT-Δ29*Tf*NCS (final concentration 0.1 mg/mL). The mixture was stirred at 37 ^{o}C under argon. After 6 h, the reaction was quenched with aq. HCl (4 mL) and adjusted to pH 7.5 by adding aq. Na_2CO_3 (1 M). The mixture was then stirred for another 4 h at 60 ^{o}C under argon and extracted with ethyl acetate (4 × 20 mL). The organic layer was washed with brine (3 × 40 mL), dried (Na_2SO_4) and evaporated to give the crude product. The residue was re-suspended in aq. HCl (10 mL, 1 M) and DMC (10 mL), and the aqueous layer was washed with DMC (3 × 5 mL). DMC fractions were combined and washed with aq. HCl (3 × 5 mL, 1 M). The aqueous phase was co-evaporated with methanol at 55 ^{o}C to obtain a white precipitate, (-)-trolline (*S*)-**2** (65 mg, 74%). No minor ortho-regioisomer was observed, and derivatisation of both phenolic groups with (*R*)-(-)-methoxy-α-trifluoromethylphenylacetyl chloride confirmed the 95% *ee* of (*S*)-**2** (above) by ^{1}H NMR spectroscopy (integration of 5-H and 8-H signals signals at δ 7.161 ppm and 7.188 ppm in (*S*)-isomer *vs* δ 7.176 ppm in the (*R*)-isomer, and OMe groups on the Mosher's esters). Data for (*S*)-**2**: Mp. >180 ^{o}C (MeOH/H_2O) decomposed; $[\alpha]_D^{20}$ -141 (*c* 0.10, MeOH), Lit. $[\alpha]_D^{20}$ -197 (*c* 0.8, MeOH)[8]; IR (neat, cm^{-1}) 3343, 2926, 1643, 1524; ^{1}H NMR (600 MHz; CD_3OD) δ 6.55 (s, 2H, 5-H and 8-H), 4.71 (t, *J* = 7.8 Hz, 1H, 1-H), 4.09 (ddd, *J* = 12.3, 6.0, 2.9 Hz, 1H, 3-$\underline{H}$H), 3.05 (td, *J* = 12.3, 4.2 Hz, 1H, 3-H$\underline{H}$), 2.77–2.70 (m, 1H, 4-$\underline{H}$H), 2.67–2.60 (m, 2H, 4-H$\underline{H}$ and 1'-$\underline{H}$H), 2.60–2.54 (m, 1H, 2'-$\underline{H}$H), 2.40 (dd, *J* = 15.6, 9.6 Hz, 1H, 2'-H$\underline{H}$), 1.83–1.73 (app. quint, *J* = 9.6 Hz, 1H, 1'-H$\underline{H}$); ^{13}C NMR (151 MHz; CD_3OD) δ 175.9 (C=O), 145.6, 145.5, 129.8 (C-8a), 125.5 (C-4a), 116.2 (C-5), 112.4 (C-8), 58.3 (C-1), 38.6 (C-3), 32.7 (C-2'), 28.8 (signals superimposed); *m/z* [CI] 439 ([2M+H]$^{+}$, 21%), 237 ([MH+NH_3]$^{+}$, 89), 220 ([MH]$^{+}$, 100).

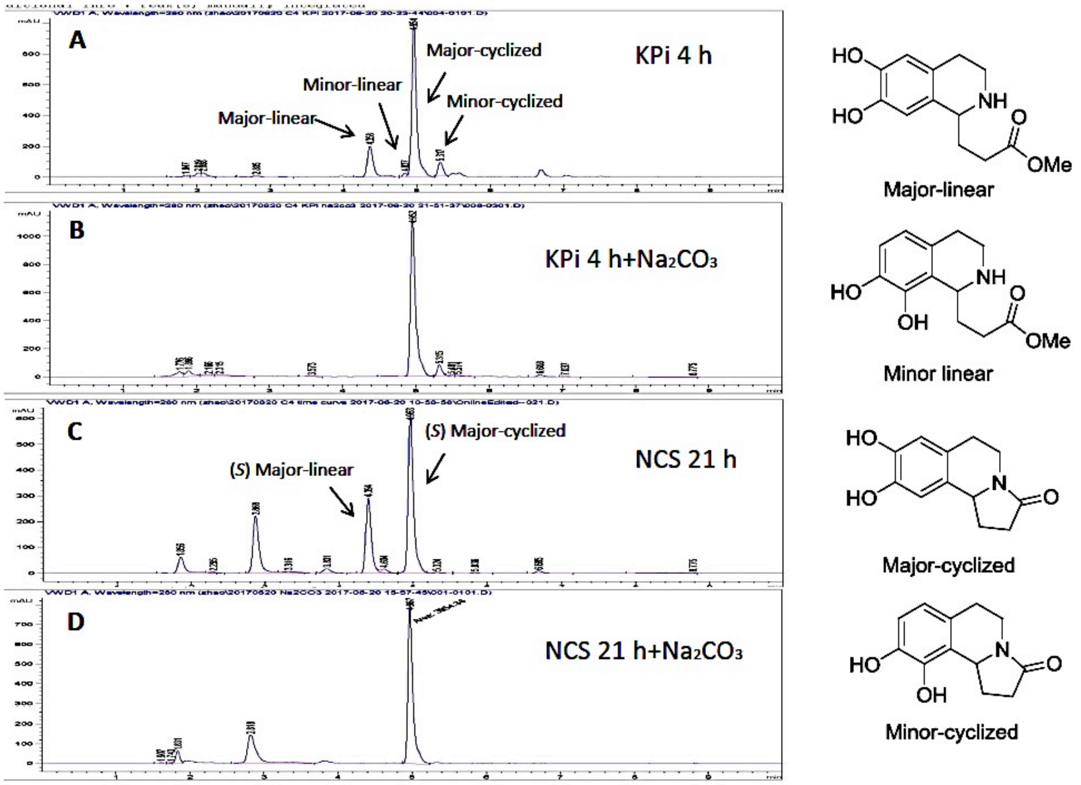

Supplementary Figure 8. Chromatogram of the KPi and NCS mediated synthesis (1 mL scale) of trolline **2**.

Methyl (1*S*)-3-(6,7-dihydroxy-1,2,3,4-tetrahydroisoquinolin-1-yl)propanoate.TFA (6)

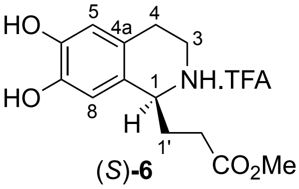

(*S*)-**6**

Synthesis of (S)-**6**. Dopamine hydrochloride (28 mg, 0.15 mmol) and sodium ascorbate (20 mg, 0.10 mmol) were added to HEPES buffer (9.73 mL, pH 7.5, 100 mM). Methyl 4-oxobutanoate **5** (12 µL, 0.10 mmol) and DMSO (0.10 mL) were then added to the solution, followed by WT-Δ29*Tf*NCS (final concentration 0.10 mg/mL). The mixture was stirred at 37 °C under argon for 3 h. The reaction mixture was quenched with aq. HCl (1 mL, 1 M), centrifuged to remove the protein and adjusted to pH 7.5 by adding NaOH (1 M). The supernatant was then washed with ethyl acetate (3 × 5 mL). The water phase was evaporated at 40 °C for 30 min to remove ethyl acetate, and purified by preparative HPLC following the *semi-preparative HPLC method B*. Product (*S*)-**6** was obtained as white solid as the TFA salt (8.4 mg, 23%) which was then used as a standard in HPLC reaction analysis. ^{1}H NMR (600 MHz; CD_3OD) δ 6.67 (s, 1H, 8-H), 6.62 (s, 1H, 5-H), 4.44 (dd, *J* = 8.1, 4.5 Hz, 1H, 1-H), 3.71 (s, 3H, OMe), 3.55-3.51 (m, 1H, 3-HH), 3.36-3.31 (m, 1H, 3-HH), 2.97 (dt, *J* = 16.8, 6.6 Hz, 1H, 4-HH), 2.91 (dt, *J* = 16.8, 6.6 Hz, 1H, 4-HH), 2.63–2.56 (m, 2H, 2'-H), 2.35–2.27 (m, 1H, 1'-HH), 2.25-2.18 (m, 1H, 1'-HH); ^{13}C NMR (151 MHz; CD_3OD) δ 174.8 (C=O), 146.9, 146.0, 123.8, 123.5, 116.3 (C-8), 114.0 (C-5), 55.9 (C-1), 52.5 (CO_2Me), 40.6 (C-3), 30.5 (C-2'), 29.8, 25.6; *m/z* [ES+] 252 ($[MH]^+$, 100%), 238 (13); *m/z* [HRMS ES+] found $[MH]^+$ 252.1243. $C_{13}H_{18}NO_4$ requires 252.1236.

9,10-Dihydroxy-1,2,3,6,7,11b-hexahydro-4*H*-pyrido[2,1-a]isoquinolin-4-one (9)

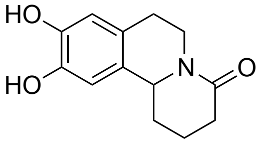

9

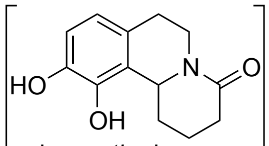

minor *ortho*-isomer

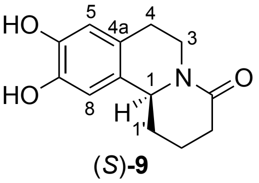

(*S*)-**9**

Synthesis of **9**.

(i) Small-scale (1 mL) KPi reaction.

The procedure described on pS11 was used and reaction yield determined by HPLC. Compound **9** was formed in 89% yield in a *para*:*ortho* isomeric ratio of 9:1 (see Supplementary Figure 9B).

(ii) Reaction for product isolation and characterisation.

To a solution of dopamine hydrochloride (19 mg, 0.10 mmol), ascorbic acid (18 mg, 0.10 mmol), KPi buffer (5 mL, pH 6, 0.3 M) and CH_3CN (5 mL) was added methyl 5-oxopantanoate **7** (22 µL, 0.15 mmol; 93% purity). The mixture was stirred at 60 °C for 18 h under argon. The pH of the solution was then adjusted to 7.5 by adding aq. Na_2CO_3 (1 M) and stirred for another 4 h at 60 °C under argon. The reaction mixture was purified directly by preparative HPLC following *semi-preparative HPLC method C* to obtain a white solid **9** (16 mg, 69%) in a *para*:*ortho* isomeric ratio of 50:1 by NMR. The characterization data is given below for the (*S*)-isomer.

Synthesis of (S)-**9**.

(i) Small-scale (1 mL) NCS reaction.

The procedure described on pS11 was used and reaction yield determined by HPLC. Compound (*S*)-**9** was was formed in 96% yield (see Supplementary Figure 9D) and no minor isomeric product was observed. The *ee* was determined to be 96% by chiral HPLC (of intermediate (*S*)-**8**:see Supplementary Figure 6B and Table S2).

(ii) Preparative scale reaction for product isolation and characterisation.

Dopamine hydrochloride (76 mg, 0.40 mmol) and sodium ascorbate (80 mg, 0.40 mmol) were added to HEPES buffer (38.9 mL, pH 7.5, 100 mM). Methyl 5-oxopantanoate **5** (80 μL, 0.60 mmol) in DMSO (0.4 mL) was then added to the solution, followed by WT-Δ29*Tf*NCS (final concentration 0.1 mg/mL). The mixture was stirred at 37 °C under argon. After 6 h, the reaction was quenched with aq. HCl (4 mL, 1 M) and adjusted to pH 7.5 by adding aq. Na_2CO_3 (1 M). The mixture was then stirred for another 4 h at 60 °C under argon and extracted with ethyl acetate (4 × 20 mL). The organic layer was washed with brine (3 × 40 mL), dried (Na_2SO_4) and evaporated to give the crude product. The residue was re-suspended in aq. HCl (10 mL, 1 M) and DMC (10 mL), and the aqueous layer was washed with DMC (3 × 5 mL). The DMC fractions were combined and washed with aq. HCl (3 × 5 mL, 1 M). The aqueous phase was co-evaporated with methanol at 55 °C to obtain a white precipitate (*S*)-**9** (81 mg, 87%). No minor regioisomer was observed. Mp. 204-206 °C (MeOH/H_2O); $[\alpha]_D^{20}$ -186 (*c* 0.18, MeOH); IR (neat, cm^{-1}) 3522, 3041, 2292, 1644, 1520; ^1H NMR (600 MHz; CD_3OD) δ 6.70 (s, 1H, 8-H), 6.59 (s, 1H, 5-H), 4.69 (dd, *J* = 10.2, 3.6 Hz, 1H, 1-H), 4.44 (dt, *J* = 12.6, 4.6 Hz, 1H, 3-HH), 3.18 (app. td, *J* = 11.4, 3.6 Hz, 1H, 3-HH), 2.81 (ddd, *J* = 15.0, 10.1, 4.6 Hz, 1H, 4-HH), 2.73–2.63 (m, 2H, 4-HH and 3'-HH), 2.62–2.49 (m, 2H, 1'-HH and 3'-HH), 2.05–1.83 (m, 2H, 2'-H), 1.68 (m, 1H, 1'-HH); ^{13}C NMR (151 MHz; CD_3OD) δ 173.8 (C=O), 145.7, 145.4, 128.1 (C-8a), 126.8 (C-4a), 115.9 (C-5), 113.0 (C-8), 58.4 (C-1), 43.6 (C-3), 31.1 (C-3'), 30.4 (C-1'), 28.5 (C-4), 19.3 (C-2'); *m/z* [ES+] 467 ($[2M+H]^+$, 16%), 234 ($[MH]^+$, 100); *m/z* [HRMS ES+] found $[MH]^+$ 234.1128. $C_{13}H_{16}NO_3$ requires 234.1130.

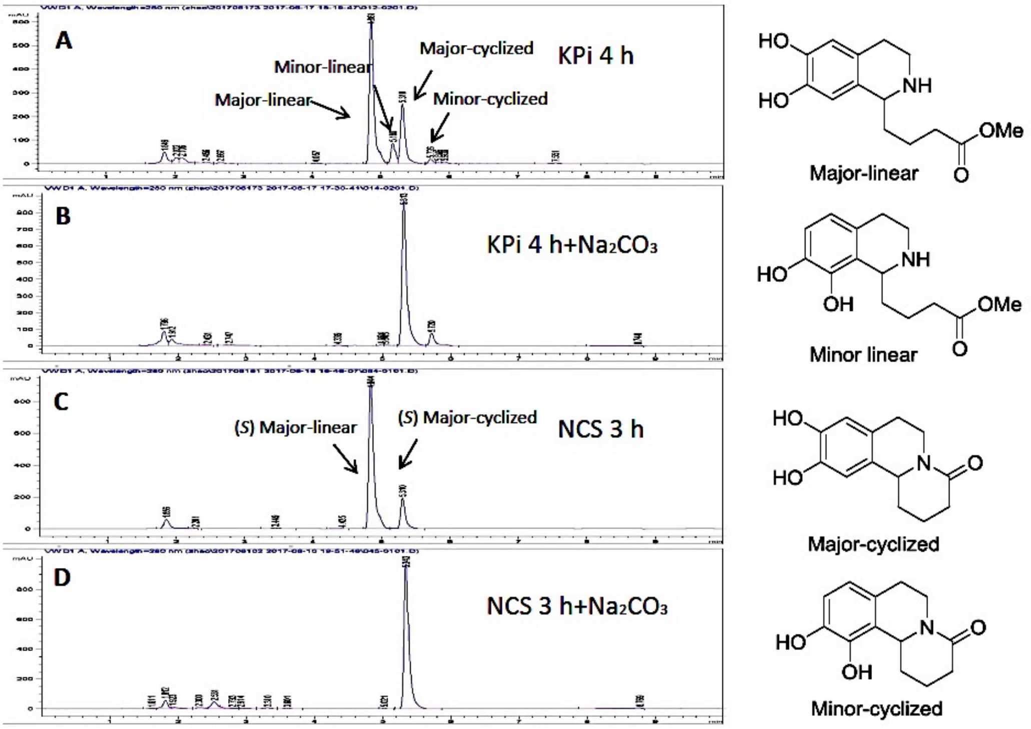

Supplementary Figure 9. Chromatogram of the KPi and NCS mediated synthesis (1 mL scale) of 9,10-dihydroxy-1,2,3,6,7,11b-hexahydro-4*H*-pyrido[2,1-a]isoquinolin-4-one **9**.

Methyl (1*S*)-4-(6,7-dihydroxy-1,2,3,4-tetrahydroisoquinolin-1-yl)butanoate.TFA (8)

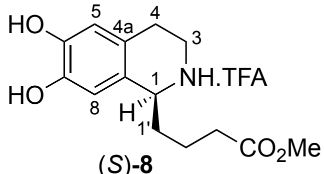

(*S*)-**8**

*Synthesis of (S)-***8**. Dopamine hydrochloride (19 mg, 0.10 mmol) and sodium ascorbate (20 mg, 0.10 mmol) were added to HEPES buffer (9.3 mL, pH 7.5, 100 mM). Methyl 5-oxopantanoate **5** (21 µL, 0.15 mmol) in DMSO (0.10 mL) was then added to the solution, followed by WT-Δ29*Tf*NCS (final concentration 0.10 mg/mL). The mixture was stirred at 37 °C under argon for 6 h. The reaction mixture was centrifuged to remove protein, and the supernatant was washed with ethyl acetate (3 × 5 mL). The aqueous phase was evaporated at 40 °C for 30 min to remove the residue ethyl acetate, and purified by preparative HPLC following the *semi-preparative HPLC method A*. Product (*S*)-**8** was obtained as a white solid as the TFA salt (7.1 mg, 19%) which was then used as a standard in HPLC reaction analysis. ^{1}H NMR (600 MHz; CD_3OD) δ 6.65 (s, 1H, 8-H), 6.61 (s, 1H, 5-H), 4.36 (dd, *J* = 8.1, 4.4 Hz, 1H, 1-H), 3.68 (s, 3H, OMe), 3.51 (dt, *J* = 12.6, 6.0 Hz, 1H, 3-HH), 3.35–3.31 (m, 1H, 3-HH), 2.98 (dt, *J* = 16.8, 6.0 Hz, 1H, 4-HH), 2.90 (dt, *J* = 16.8, 6.0 Hz, 1H, 4-HH), 2.52–2.42 (m, 2H, 3'-H), 2.08–2.03 (m, 1H, 1'-HH), 1.97–1.91 (m, 1H, 1'-HH), 1.78 (m, 2H, 2'-H); ^{13}C NMR (151 MHz; CD_3OD) δ 175.4 (C=O), 146.8, 145.9, 123.9, 123.7, 116.3 (C-8), 113.9 (C-5), 56.4 (C-1), 52.3 (CO_2Me), 40.9 (C-3), 34.3, 34.0, 25.6, 21.6; *m/z* [ES+] 266 ([MH]$^+$, 100%), 234 (15); *m/z* [HRMS ES+] found [MH]$^+$ 266.1392. $C_{14}H_{20}NO_4$ requires 266.1392.

Methyl 5-(6,7-dihydroxy-1,2,3,4-tetrahydroisoquinolin-1-yl)pentanoate (11)

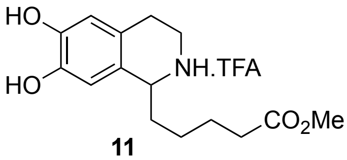

11

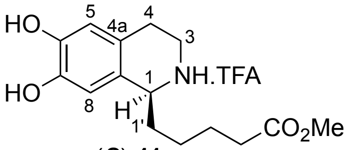

(*S*)-**11**

Synthesis of **11**.

(i) Small-scale (1 mL) KPi reaction.

The procedure described on pS11 was used and reaction yield determined by HPLC. Compound **11** was formed in 72% yield in a *para*:*ortho* isomeric ratio of 7:1 (see Supplementary Figure 10A).

(ii) Reaction for product isolation and characterisation.

To a solution of dopamine hydrochloride (9.5 mg, 0.05 mmol), ascorbic acid (9.0 mg, 0.051 mmol), KPi buffer (2.5 mL, pH 6, 0.3 M) and CH_3CN (2.5 mL) was added methyl 6-oxohexanoate **7** (11 µL, 0.075 mmol). The reaction was stirred at 60 °C for 18 h under argon, then purified using a C_{18} reverse phase semi-preparative column (*semi-preparative HPLC method A*) to obtain a white solid, **11** as the TFA salt (5.0 mg, 26%) in a *para*:*ortho* isomeric ratio of 7:1. Mp. 115-117 °C (CH_3CN/H_2O); IR (neat, cm^{-1}) 2773, 1702, 1476; ^{1}H NMR (600 MHz; CD_3OD) δ 6.64 (s, 1H, 8-H), 6.60 (s, 1H, 5-H), 4.35 (dd, *J* = 8.1, 5.1 Hz, 1H, 1-H), 3.66 (s, 3H, OMe), 3.51 (dt, *J* = 18.0, 6.0 Hz, 1H, 3-HH), 3.33–3.31 (m, 1H, 3-HH), 3.04–2.94 (m, 1H, 4-HH), 2.89 (dt, *J* = 16.8, 6.0 Hz, 1H, 4-HH), 2.38 (t, *J* =7.2 Hz, 2H, 4'-H), 2.09–2.01 (m, 1H, 1'-HH), 1.93–1.85 (m, 1H, 1'-HH), 1.80–1.66 (m, 2H, 2'-H), 1.57–1.46 (m, 2H, 3'-H); ^{13}C NMR (151 MHz; CD_3OD) δ 175.6 (C=O), 146.7, 145.9, 124.1, 123.6, 116.2 (C-8), 113.9 (C-5), 56.5

(C-1), 52.1 (CO_2Me), 40.9 (C-3), 34.8, 34.3, 25.8 (signals superimposed); *m/z* [ES+] 280 ($[MH]^+$, 100%); *m/z* [HRMS ES+] found $[MH]^+$ 280.1547. $C_{15}H_{22}NO_4$ requires 280.1549.

*Synthesis of (S)-***11**.

(i) Small-scale (1 mL) NCS reaction.

The procedure described on pS11 was used and reaction yield determined by HPLC. Compound (*S*)-**11** was was formed in 92% yield (see Supplementary Figure 10B) and no minor isomeric product was observed. The *ee* was determined to be >99.5% by chiral HPLC (see Supplementary Figure 6C and Table S2).

(ii) Reaction for product isolation and characterisation.

Dopamine hydrochloride (10.0 mg, 0.053 mmol) and sodium ascorbate (11.0 mg, 0.056 mmol) were added to HEPES buffer (4.86 mL, pH 7.5, 100 mM). Methyl 6-oxopantanoate **7** (11 µL, 0.075 mmol) in DMSO (50 µL) was added to the solution, followed by WT-Δ29*Tf*NCS (final concentration 0.1 mg/mL) to make the total volume 5 mL. The mixture was stirred at 37 °C under argon. The reaction was quenched after 3 h by adding HCl (0.5 mL, 1 M) and centrifuged for 10 min at 4000 rpm to remove the protein. The supernatant was purified by preparative HPLC following *semi-preparative HPLC method A*. The purified product was further washed with diethyl ether (3 × 2 mL) and dried under high vacuum to obtain a white solid *(S)*-**11** as the TFA salt (13 mg, 63%). No minor isomeric product was observed. The characterisation data was identical to that above for the racemate and $[\alpha]_D^{20}$ = -34.4 (c 0.13, MeOH).

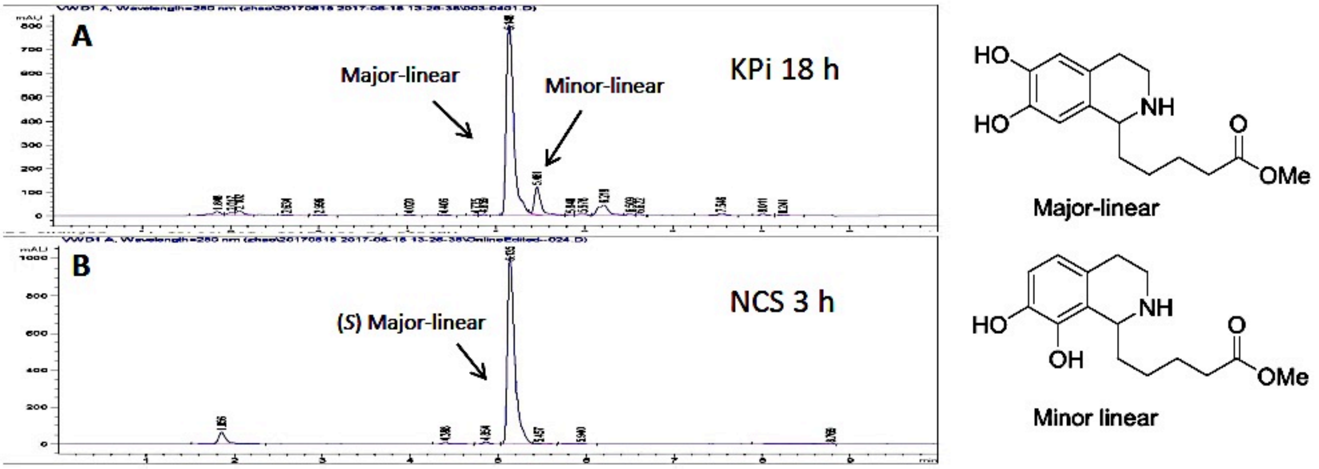

Supplementary Figure 9 Chromatogram of KPi and NCS mediated synthesis (1 mL scale) of methyl 5-(6,7-dihydroxy-1,2,3,4-tetrahydroisoquinolin-1-yl)pentanoate **11**

2-(3-Hydroxyphenyl)ethylamine (13)[3]

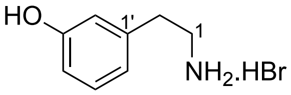

To a stirred solution of 3-methoxyphenylacetonitrile (625 mg, 4.25 mmol) in THF (10 mL) at 0 °C was added BH_3-THF solution (11.0 mL, 11.0 mmol; 1 M). The mixture was gradually warmed to room temperature. After stirring for 24 h, the reaction was cooled to 0 °C and methanol (15 mL) was added dropwise. Then the mixture was warmed to room temperature, stirred for 8 h and concentrated under

vacuum. The residue was dissolved in methanol (20 mL) and evaporated to obtain the crude product, which was purified by SiO_2 column chromatography (CH_2Cl_2 and MeOH (with 5% Et_3N), 25:1 to 5:1), giving 3-methoxyphenethylamine as a pale yellow oil (440 mg, 68%). ^{1}H NMR (300 MHz; $CDCl_3$) δ 7.24 (m, 1H, 5'-H), 6.83–6.75 (m, 3H, 2'-H, 4'-H and 6'-H), 3.80 (s, 3H, OCH_3), 2.97 (m, 2H, 1-H), 2.73 (m, 2H, 2-H).

3-Methoxyphenethylamine (350 mg, 2.31 mmol) was added to anhydrous dichloromethane (10 mL) and the reaction was stirred at -78 ^{o}C. Then boron tribromide (5.1 mL, 5.1 mmol; 1 M) was added dropwise. The mixture was warmed to room temperature and stirred for 12 h. Methanol (10 mL) was added dropwise and the reaction was stirred for another 3 h, then concentrated under vacuum. The residue was dissolved in methanol (10 mL) and evaporated under vacuum, which was repeated several times. The product **13** was obtained as the HBr salt (502 mg, 99%). ^{1}H NMR (600 MHz; CD_3OD) δ 7.15 (t, J = 7.8 Hz, 1H, 5'-H), 6.74 (ddd, J = 7.8, 2.4, 0.6 Hz, 1H, 4'-H), 6.72 (m, 1H, 2'-H), 6.70 (ddd, J = 7.8, 2.4, 0.6 Hz, 1H, 6'-H), 3.16 (t, J = 7.8 Hz, 2H, 1-H), 2.89 (t, J = 7.8 Hz, 2H, 2-H); ^{13}C NMR (151 MHz; CD_3OD) δ 159.0 (C-3'), 139.3 (C-1'), 131.1 (C-5'), 120.8, 116.6, 115.2, 42.0 (C-1), 34.5 (C-2); *m/z* [Cl] 155 ($[MNa]^+$, 23%), 138 ($[MH]^+$, 100).

2-(4-Fluoro-3-hydroxyphenyl)ethylamine hydrobromide (15)[9]

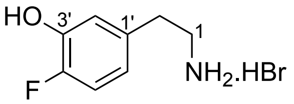

To a stirred solution of sodium carbonate (2.0 g, 19 mmol) and methylamine hydrochloride (2.0 g, 30 mmol) in ethanol (25 mL) was added 4-fluoro-3-methoxybenzaldehyde (9.4 g, 61 mmol) in EtOH (20 mL). Nitromethane (5.3 mL, 94 mmol) was added and the mixture was sealed and left in dark at room temperature. Three days later, the yellow precipitate formed was filtered and washed with cold ethanol, giving the 2-(4-fluoro-3-methoxyphenyl)nitroethylene as yellow crystals (6.3 g, 52% yield). ^{1}H NMR (300 MHz; CD_3OD) δ 8.04 (d, J = 13.5 Hz, 1H, $=CHNO_2$), 7.92 (d, J = 13.5 Hz, 1H, ArC$\underline{H}$=CH), 7.47 (dd, $^4J_{HF}$ = 8.4 Hz, J_{HHm} = 2.1 Hz, 1H, 2'-H), 7.29 (ddd, J_{HHo} = 8.4 Hz, $^4J_{HF}$ = 4.5 Hz, J_{HHm} = 2.1 Hz, 1H, 6'-H), 7.18 (dd, $^3J_{HF}$ = 11.1 Hz, J_{HHo} = 8.4 Hz, 1H, 5'-H), 3.93 (s, 3H, OCH_3).

A solution of 2-(4-fluoro-3-methoxyphenyl) nitroethylene (1.0 g, 5.1 mmol) in anhydrous THF (25 mL) was added dropwise to $LiAlH_4$-THF solution (15 mL, 15 mmol; 1 M) at 0 ^{o}C. The mixture was warmed to room temperature, stirred for 12 h and then quenched by adding H_2O (0.7 mL), 15% NaOH (0.7 mL) and H_2O (2 mL). The solution was filtered, dried (Na_2SO_4), evaporated and purified by SiO_2 column chromatography (CH_2Cl_2 and MeOH (with 5% Et_3N), 10:1 to 5:1) to give 2-(4-fluoro-3-methoxyphenyl)ethylamine as a pale yellow oil (210 mg, 24%). ^{1}H NMR (600 MHz; $CDCl_3$) δ 6.99 (dd, $^3J_{HF}$ = 11.4 Hz, J_{HHo} = 8.4 Hz, 1H, 5'-H), 6.79 (dd, $^4J_{HF}$ = 8.4 Hz, J_{HHm} = 1.8 Hz, 1H, 2'-H), 6.71 (ddd, J_{HHo} = 8.4 Hz, $^4J_{HF}$ = 4.2 Hz, J_{HHm} = 1.8 Hz, 1H, 6'-H), 3.88 (s, 3H, OCH_3), 2.95 (t, J = 7.2 Hz, 2H, $C\underline{H}_2NH_2$), 2.70 (t, J = 7.2 Hz, 2H, $C\underline{H}_2CH_2NH_2$).

A solution of 2-(4-fluoro-3-methoxyphenyl)ethylamine (210 mg, 1.24 mmol) and 48% HBr (6 mL; solution in water) was heated at reflux for 3 h. The solvent was remove *in vacuo*, the residue was dissolved with ethanol and evaporated twice. The solid was dissolved in 2-propanol (1 mL) and ether was added. The white precipitate formed was collected by filtration and dried to give **15** as the HBr salt (270 mg, 92%). ^{1}H NMR (600 MHz; CD_3OD) δ 7.01 (dd, $^3J_{HF}$ = 11.4 Hz, J_{HHo} = 8.4 Hz, 1H, 5'-H), 6.85 (dd, $^4J_{HF}$ = 8.4 Hz, J_{HHm} = 2.4 Hz, 1H, 2'-H), 6.71 (ddd, J_{HHo} = 8.4 Hz, $^4J_{HF}$ = 4.2 Hz, J_{HHm} = 2.4 Hz, 1H,

6'-H), 3.14 (t, J = 7.8 Hz, 2H, C$\underline{H}_2$NH$_2$), 2.88 (t, J = 7.8 Hz, 2H, C$\underline{H}_2$CH$_2$NH$_2$); ^{13}C NMR (151 MHz; CD$_3$OD) δ 152.3 ($^1J_{CF}$ = 240 Hz, C-4'), 146.4 ($^2J_{CF}$ = 13.6 Hz, C-3'), 134.3 ($^4J_{CF}$ = 3.0 Hz, C-1'), 121.0 ($^3J_{CF}$ = 6.0 Hz, C-2' or C-6'), 119.2 ($^3J_{CF}$ = 3.0 Hz, C-2' or C-6'), 117.2 ($^2J_{CF}$ = 19.6 Hz, C-5'), 42.0 (C-1), 33.9 (C-2); *m/z* [Cl] 173 ([MH+NH$_3$]$^+$, 24%), 156 ([MH]$^+$, 100).

8-Hydroxy-1,5,6,10b-tetrahydropyrrolo [2,1-a] isoquinolin-3(2*H*)-one (16)

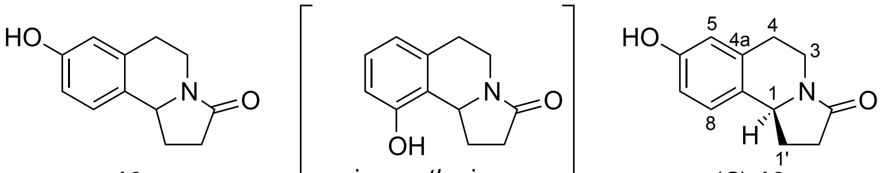

16 minor *ortho*-isomer (*S*)-**16**

Racemic synthesis of **16**.

(i) Small-scale (1 mL) KPi reaction.

The procedure described on pS11 was used and reaction yield determined by HPLC. Compound **16** was formed in 97% yield in a *para*:*ortho* isomeric ratio of 6:1 (see Supplementary Figure 11A).

(ii) Reaction for product isolation and characterisation.

To a solution of 2-(3-hydroxyphenyl)ethylamine **13** (HBr salt) (44 mg, 0.20 mmol), ascorbic acid (36 mg, 0.20 mmol), KPi buffer (10 mL, pH 6, 0.3 M) and CH$_3$CN (10 mL) was added methyl 4-oxobutanoate (32 µL, 0.30 mmol). The mixture was stirred at 60 ^{o}C for 18 h under argon. Then Na$_2$CO$_3$ (0.5 mL, 1 M) was added and the reaction was stirred for another 4 h at 60 ^{o}C. The mixture was extracted with ethyl acetate (3 × 10 mL) and the organic layer was dried (Na$_2$SO$_4$) and evaporated. The residue was re-suspended in aq. HCl (5 mL, 1 M) and DMC (5 mL), and the aqueous layer was washed with DMC (2 × 5 mL). The DMC fractions were combined and washed with aq. HCl (2 × 5 mL, 1 M). The aqueous phase was evaporated to give a white solid **16** (28 mg, 69%) in a *para*:*ortho* isomeric ratio of 10:1. Mp. >230 ^{o}C (MeOH/H$_2$O) decomposed; IR (neat, cm^{-1}) 3121, 1657, 1618, 1578; ^{1}H NMR (600 MHz; CD$_3$OD) δ 6.99 (d, J = 8.4 Hz, 1H, 8-H), 6.69 (dd, J = 8.4, 2.4 Hz, 1H, 7-H), 6.59 (d, J = 2.4 Hz, 1H, 5-H), 4.79 (t, J = 7.8 Hz, 1H, 1-H), 4.07 (ddd, J = 12.9, 6.0, 3.0 Hz, 1H, 3-$\underline{H}$H), 3.16–3.09 (m, 1H, 3-H$\underline{H}$), 2.84–2.80 (m, 1H, 4-$\underline{H}$H), 2.78–2.66 (m, 2H, 4-H$\underline{H}$ and 1'-$\underline{H}$H), 2.65–2.54 (m, 1H, 2'-$\underline{H}$H), 2.40 (ddd, J = 16.2, 9.0, 1.5 Hz, 1H, 2'-H$\underline{H}$), 1.81 (m, 1H, 1'-H$\underline{H}$); ^{13}C NMR (151 MHz; CD$_3$OD) δ 176.0 (C=O), 157.4 (C-6), 135.9, 129.7, 126.9 (C-8), 116.0 (C-5), 115.4 (C-7), 58.4 (C-1), 38.6 (C-3), 32.7, 29.5, 28.7; *m/z* [ES+] 204 ([MH]$^+$, 100%), 102 (33); *m/z* [HRMS ES+] found [MH]$^+$ 204.1023. C$_{12}$H$_{14}$NO$_2$ requires 204.1024.

Synthesis of (S)-**16**.

(i) Small-scale (1 mL) NCS reaction.

The procedure described on pS11 was used and reaction yield determined by HPLC. Compound (*S*)-**16** was was formed in 51% yield (see Supplementary Figure 11C) and no minor isomeric product was observed. The *ee* was determined to be 93% by chiral HPLC (see Supplementary Figure 6D and Table S2).

(ii) Reaction for product isolation and characterisation.

2-(3-Hydroxyphenyl)ethylamine **13** (HBr salt) (11.0 mg, 0.050 mmol) and sodium ascorbate (9.0 mg, 0.045 mmol) were added to HEPES buffer (4.02 mL, pH 7.5, 100 mM). Methyl 4-oxobutanoate (9 µL, 0.075 mmol, 95% purity) in DMSO (0.5 mL, 10% v/v) was added to the solution, followed by WT-

Δ29*Tf*NCS (final concentration 0.5 mg/mL) to a total volume of 5 mL. The mixture was stirred at 37 °C under argon. After 18 h, the reaction was quenched with aq. HCl (0.5 mL, 1 M) and adjusted to pH 7.5 by adding aq. Na_2CO_3 (0.75 mL, 1 M). The mixture was then stirred for another 4 h at 50 °C under argon. The reaction mixture was then centrifuged to remove proteins, and purified by preparative HPLC following *semi-preparative HPLC method B* to obtain *(S)*-**16** (1.9 mg, 19%) as a yellow solid. The characterization data were consistent with that described above and $[\alpha]_D^{20}$ -20 (*c* 0.2, MeOH), but no minor regioisomer was present (see spectra).

In addition, experiments were performed to investigate the possible racemisation of the products such as (*S*)-**16**. When compound (*S*)-**16** was stirred at 60 °C for 4 h stirred at 60 °C for 4 h in HEPES buffer (and 1% DMSO) under Ar with sodium ascorbate, Na_2CO_3 (1 M) and at pH 7-8, the *ee* afterwards was the same within experimental error to that before being subjected to these reaction conditions (0 to 1% *ee* difference).

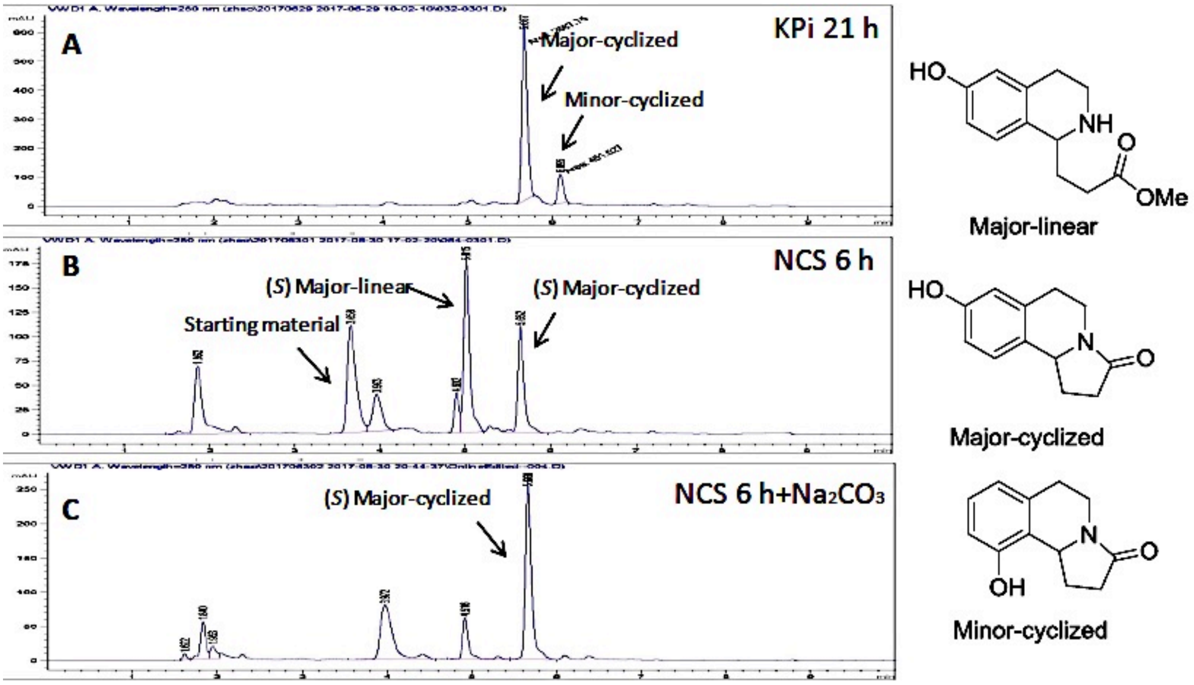

Supplementary Figure 11. Chromatogram of KPi and NCS mediated synthesis (1 mL scale) of 8-hydroxy-1,5,6,10b-tetrahydropyrrolo [2,1-a] isoquinolin-3(2*H*)-one **16**.

9-Fluoro-8-hydroxy-1,5,6,10b-tetrahydropyrrolo [2,1-a] isoquinolin-3(2*H*)-one (18)

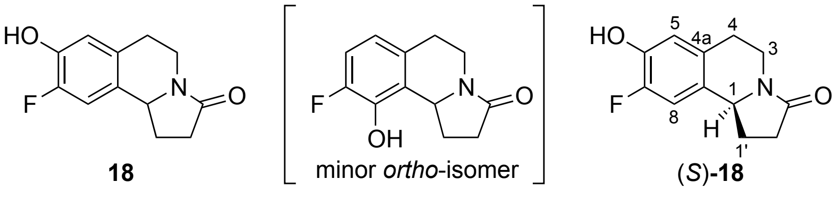

Synthesis of **18**.

(i) Small-scale (1 mL) KPi reaction.

The procedure described on pS11 was used and reaction yield determined by HPLC. Compound **18** was formed in 100% yield in a *para*:*ortho* isomeric ratio of 13:1 (see Supplementary Figure 12A).

(ii) Reaction for product isolation and characterisation.

To a solution of 2-(4-fluoro-3-hydroxyphenyl)ethylamine (HBr salt) **15** (47 mg, 0.20 mmol), ascorbic acid (36 mg, 0.20 mmol), KPi buffer (10 mL, pH 6, 0.3 M) and CH_3CN (10 mL) was added methyl 4-oxobutanoate (32 µL, 0.31 mmol). The mixture was stirred at 60 for 18 h under argon. Then 1 M Na_2CO_3 was added to adjust the pH to 7.5 and the reaction was stirred for another 3 h at 60 ^{o}C. The mixture was extracted with ethyl acetate (3 × 10 mL), the organic layer was dried (Na_2SO_4) and evaporated. The residue was re-suspended in aq. HCl (10 mL, 1 M) and DMC (10 mL), and the aqueous layer was washed with DMC (2 × 5 mL). The DMC fractions were combined and washed with aq. HCl (2 × 5 mL, 1 M). The aqueous phase was evaporated to give **18** (25 mg, 57%), in a *para:ortho* ratio of 10:1, as a yellow solid. The spectroscopic characterization data was consistent with that given below for the NCS generated compound.

*Synthesis of (S)-***18**.

(i) Small-scale (1 mL) NCS reaction.

The procedure described on pS11 was used and reaction yield determined by HPLC. Compound (*S*)-**18** was was formed in 57% yield (see Supplementary Figure 12C) and no minor isomeric product was observed. The *ee* was determined to be 87% by chiral HPLC (see Supplementary Figure 6E and Table S2).

(ii) Reaction for product isolation and characterisation.

2-(4-Fluoro-3-hydroxyphenyl)ethylamine **15** (12 mg, 0.053 mmol) and sodium ascorbate (9.0 mg, 0.045 mmol) were added to HEPES buffer (4.52 mL, pH 7.5, 100 mM). Methyl 4-oxobutanoate (9 µL, 0.075 mmol; 95% purity) in DMSO (10 µL) was added to the solution, followed by WT-Δ29*Tf*NCS (final concentration 0.5 mg/mL) to a volume of 5 mL. The mixture was stirred at 37 ^{o}C under argon. After 15 h, the reaction was quenched with aq. HCl (0.5 mL) and adjusted to pH 7.5 by adding aq. Na_2CO_3 (0.75 mL, 1 M). The mixture was then stirred for another 4 h at 60 ^{o}C under argon. The reaction mixture was centrifuged to remove protein, and purified by preparative HPLC following s*emi-preparative HPLC method B* to obtain a white solid, *(S)*-**18** (4.8 mg, 41%). Mp. 192-195 ^{o}C; $[\alpha]_D^{20}$ -109.1 (*c* 0.22, MeOH); IR (neat, cm^{-1}) 3365, 1640, 1617, 1597, 1522; ^{1}H NMR (600 MHz; CD_3OD) δ 6.88 (d, $^{3}J_{HF}$ = 11.4 Hz, 1H, 8-H), 6.71 (d, $^{4}J_{HF}$ = 8.4 Hz, 1H, 5-H), 4.75 (t, *J* = 8.0 Hz, 1H, 1-H), 4.09 (ddd, *J* = 13.2, 5.4, 2.4 Hz, 1H, 3-$\underline{H}$H), 3.10–3.04 (m, 1H, 3-H$\underline{H}$), 2.80–2.65 (m, 3H, 4-H and 1'-$\underline{H}$H), 2.64–2.58 (m, 1H, 2'-$\underline{H}$H), 2.41 (ddd, *J* = 16.2, 9.0, 1.2 Hz, 1H, 2'-H$\underline{H}$), 1.80–1.77 (m, 1H, 1'-H$\underline{H}$); ^{13}C NMR (151 MHz; CD_3OD) δ 176.0 (C=O), 152.0 ($^{1}J_{CF}$ = 240 Hz, C-7), 145.0 ($^{2}J_{CF}$ = 13.6 Hz, C-6), 130.8 ($^{4}J_{CF}$ = 3.0 Hz, C-4a), 130.0 ($^{3}J_{CF}$ = 6.0 Hz, C-8a), 118.6 ($^{3}J_{CF}$ = 3.0 Hz C-5), 113.2 ($^{2}J_{CF}$ = 19.5 Hz, C-8), 58.2 (C-1), 38.5 (C-3), 32.6, 28.8, 28.6; *m/z* [ES+] 443 ($[2M+H]^+$, 51%), 222 ($[MH]^+$, 100), 214 (17); *m/z* [HRMS ES+] found $[MH]^+$ 222.0934. $C_{12}H_{13}FNO_2$ requires 222.0930.

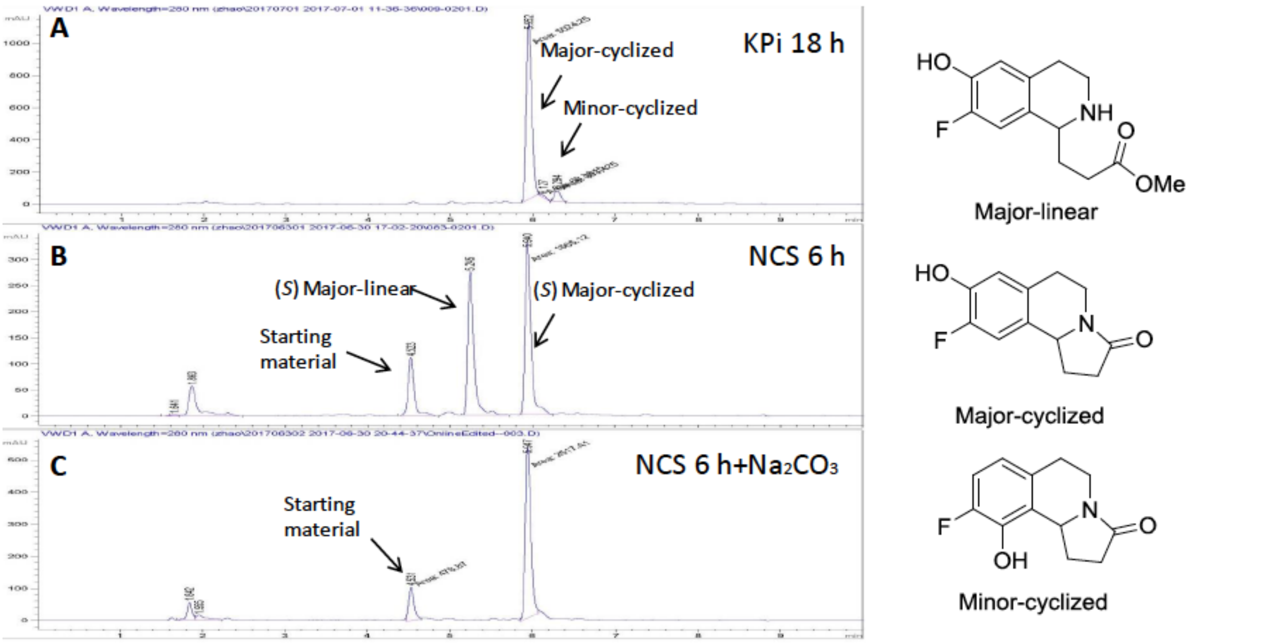

Supplementary Figure 12. Chromatogram of KPi and NCS mediated synthesis (1 mL scale) of 9-fluoro-8-hydroxy-1,5,6,10b-tetrahydropyrrolo [2,1-a] isoquinolin-3(2*H*)-one **18**.

(5*S*,6*R*,10b*S*)-6,8-Dihydroxy-5-methyl-1,5,6,10b–tetrahydropyrrolo[2,1-a]isoquinolin-3(2*H*)-one (17)

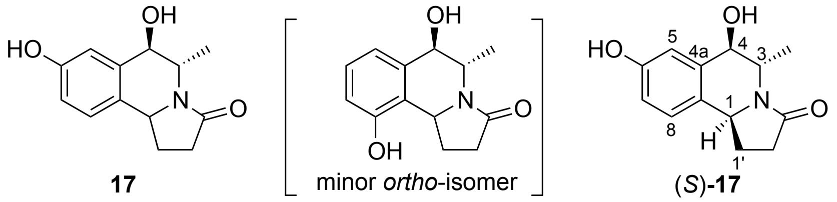

17 minor *ortho*-isomer (*S*)-**17**

Synthesis of **17**.

(i) Small-scale (1 mL) KPi reaction.

The procedure described on pS11 was used and reaction yield determined by HPLC. Compound **17** (all isomers (*S*)-**17**, (*R*)-**17** and an *ortho*-product) was formed in 82% yield (See Supplementary Figure 13B).

(ii) Preparative scale reaction for product isolation and characterisation.

To a solution of Metaraminol (+)-bitartrate salt **14** (64 mg, 0.20 mmol), KPi buffer (18 mL, pH 7, 1 M) and CH_3CN (2 mL) was added methyl 4-oxobutanoate (32 µL, 0.31 mmol). The mixture was stirred at 60 °C for 18 h under argon. Then 1 M Na_2CO_3 was added to adjust the pH to 7.5 and the reaction was stirred for another 4 h at 50 °C. The reaction mixture was then centrifuged, and the supernatant was purified directly by C_{18} preparative HPLC following the *semi-preparative HPLC method A*, giving the product as a mixture of (*S*)-**17**, (*R*)-**17** and an *ortho*-product (Ratio, 100:76:47, Supplementary Figure 14A) (5.0 mg, 11% combined yield).

*Synthesis of (*1*S)*-**17**.

(i) Small-scale (1 mL) NCS reaction.

The procedure described on pS11 was used and reaction yield determined by HPLC. Compound (*S*)-**17** was was formed in 76% yield (see Supplementary Figure 13D). The ratio of (1*S*)-**17**, (1*R*)-**17** and an *ortho*-product was confirmed to be 100:4:6 by ^{1}H NMR spectroscopy (SI Figure 14) and HPLC.

(ii) Preparative scale reaction for product isolation and characterisation.

Metaraminol (+)-bitartrate salt **14** (16.5 mg, 0.052 mmol) and sodium ascorbate (10.9 mg, 0.056 mmol) were dissolved in HEPES buffer (4 mL, pH 7.5, 100 mM). Methyl 4-oxobutanoate (8 µL, 0.075 mmol) in acetonitrile (0.5 mL) was added to the solution, followed by WT-Δ29*Tf*NCS stock solution (final concentration 0.5 mg/mL) to make the total volume 5 mL. The mixture was stirred at 37 ^{o}C under argon. After 18 h, the reaction was quenched with aq. HCl (1 mL, 1 M) and adjusted to pH 7.5 by adding aq. Na_2CO_3 (1 M). The mixture was stirred for another 3 h at 60 ^{o}C under argon. The reaction mixture was then centrifuged, and the supernatant purified directly by C_{18} preparative HPLC following the *semi-preparative HPLC method A* to give a white solid (1*S*)-**17** (6.0 mg, 49%). The ratio of isomers present was confirmed by ^{1}H NMR spectroscopy (SI Figure 14) and HPLC. An NOE signal between 1-H and CH_3 was observed indicating the stereochemistry at C-1 as being the *S*-configuration. Mp. >260 ^{o}C decomposed; $[\alpha]_D^{20}$ -94.7 (*c* 0.15, MeOH); IR (neat, cm^{-1}) 3064, 2970, 2913, 2835, 1646, 1611; ^{1}H NMR (600 MHz; CD_3OD) δ 7.06 (d, *J* = 8.4 Hz, 1H, 8-H), 6.81 (dd, *J* = 8.4, 2.4 Hz, 1H, 7-H), 6.77 (d, *J* = 2.4 Hz, 1H, 5-H), 4.80 (dd, *J* = 9.6, 7.2 Hz, 1H, 1-H), 4.44 (qd, *J* = 7.2, 2.1 Hz, 1H, 3-H), 4.40 (d, *J* = 2.1 Hz, 1H, 4-H), 2.75–2.65 (m, 1H, 1'-H̲H), 2.63–2.56 (m, 1H, 2'-H̲H), 2.42 (ddd, *J* = 16.2, 9.6, 1.2 Hz, 1H, 2'-HH̲), 1.92–1.83 (m, 1H, 1'-HH̲), 1.08 (d, *J* = 7.2 Hz, 3H, CH_3); ^{13}C NMR (151 MHz; CD_3OD) δ 177.0 (C=O), 157.7 (C-6), 135.5 (C-4a), 129.0 (C-8a), 127.1 (C-8), 118.0, 117.4, 72.2 (C-4), 55.2 (C-1), 51.1 (C-3), 32.9, 28.9, 14.8 (CH_3); *m/z* [CI] 467 ([2M+H]$^+$, 100%), 251 ([MH+NH_3]$^+$, 39), 234 ([MH]$^+$, 97); *m/z* [HRMS CI] found [MH]$^+$ 234.1125. $C_{13}H_{16}NO_3$ requires 234.1125.

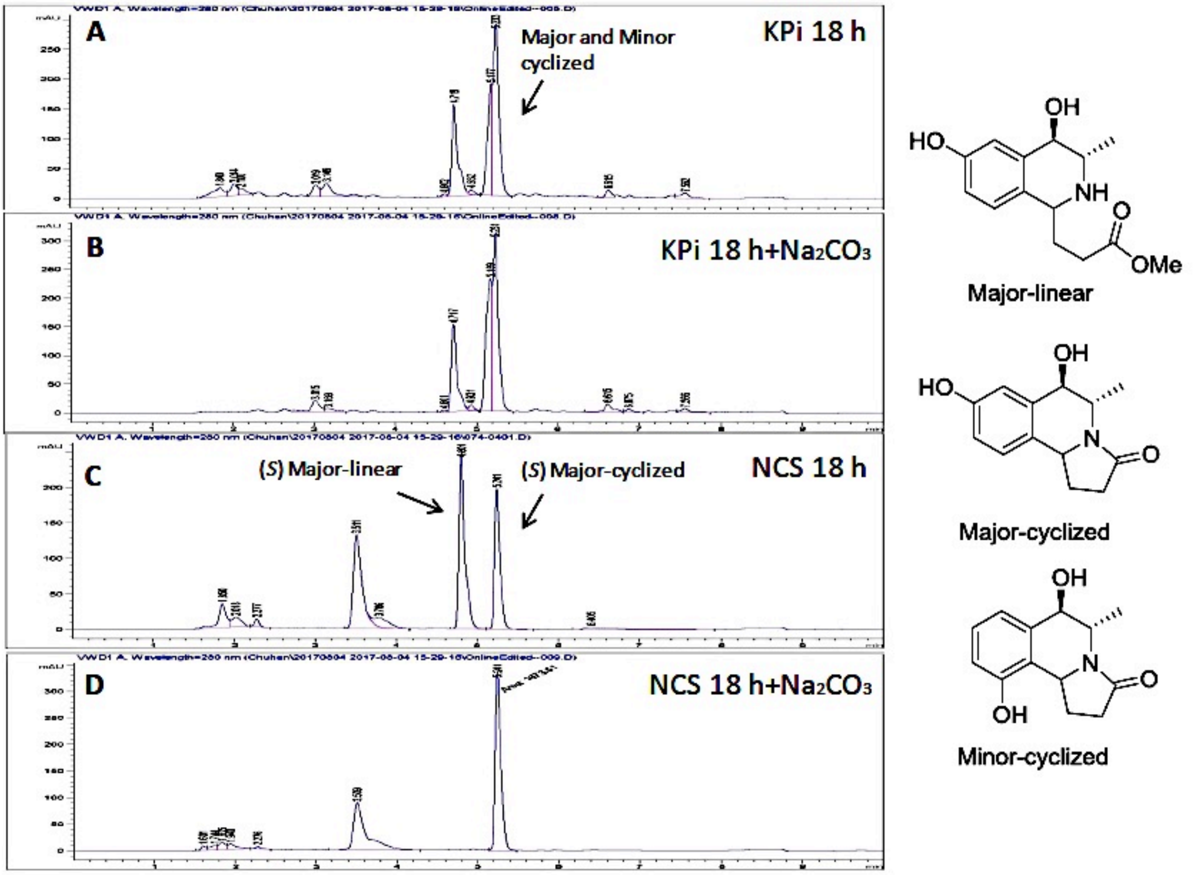

Supplementary Figure 13. Chromatogram of KPi and NCS mediated synthesis (1 mL scale) of (5*S*,6*R*,10b*S*)-6,8-Dihydroxy-5-methyl-1,5,6,10b–tetrahydropyrrolo[2,1-a]isoquinolin-3(2*H*)-one **17.**

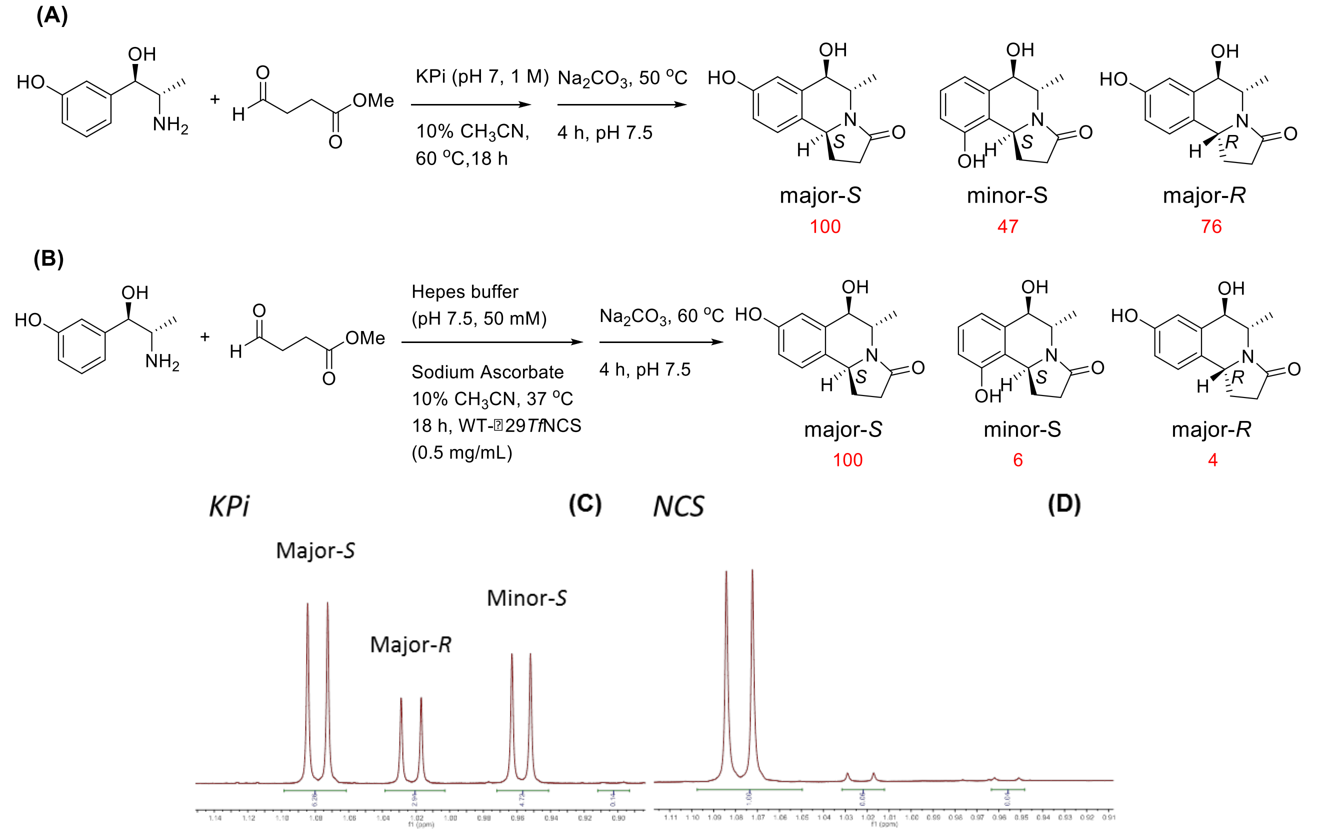

Supplementary Figure 14. 5 mL Scale up KPi (A) and NCS (B) reactions with metaraminol **14**; ^{1}H NMR spectra of isolated product **17** (C) and (1*S*)-**17** (D) and the ratio of the regioisomers and diastereoisomers.

4 Spectral Data

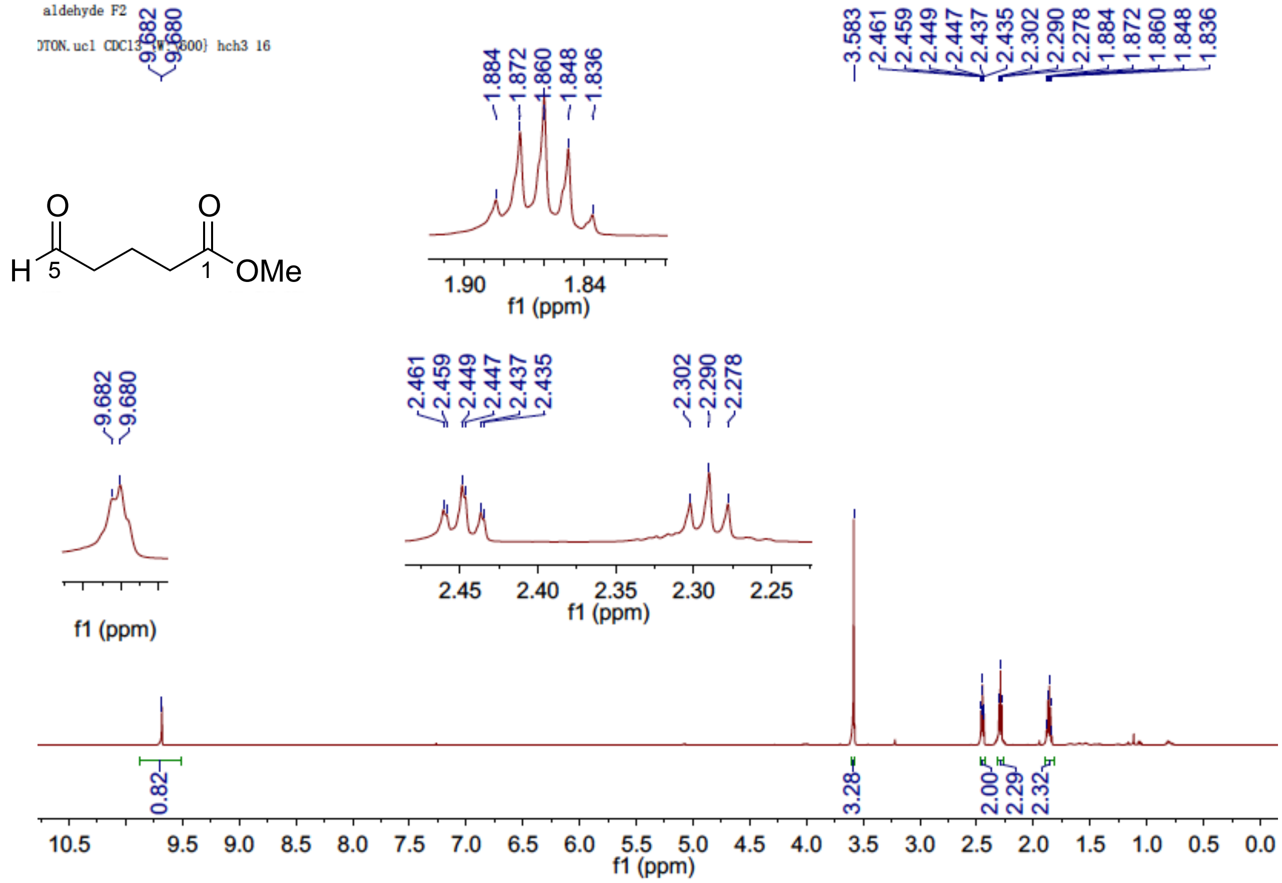

^{1}H NMR spectrum (compound **7**)

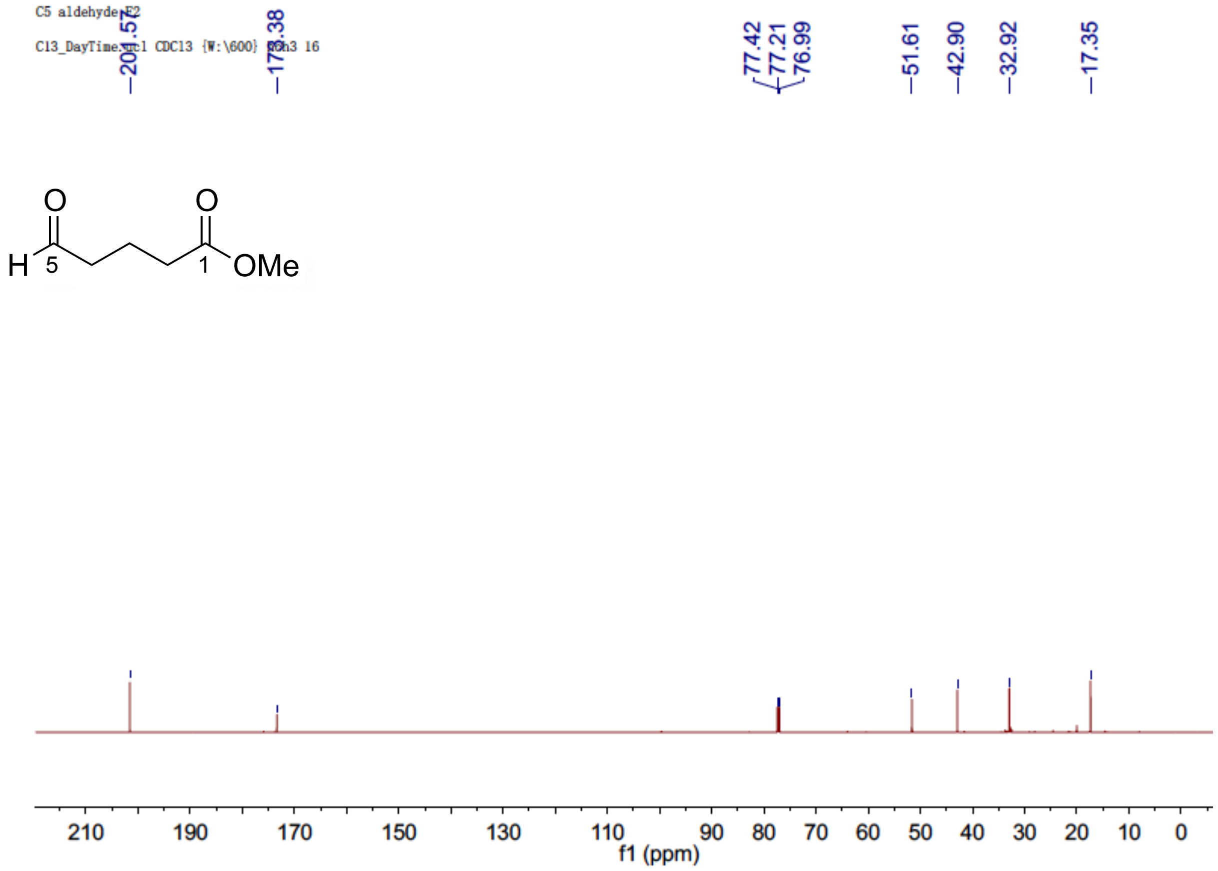

^{13}C NMR spectrum (compound **7**)

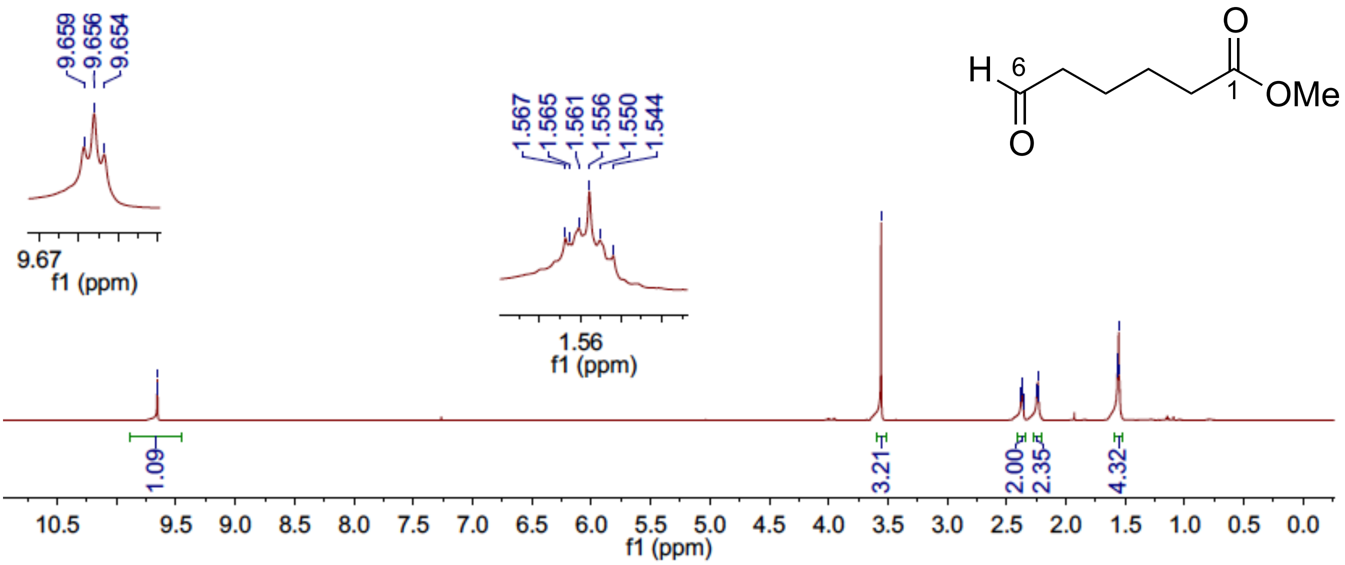

^{1}H NMR spectrum (compound **10**)

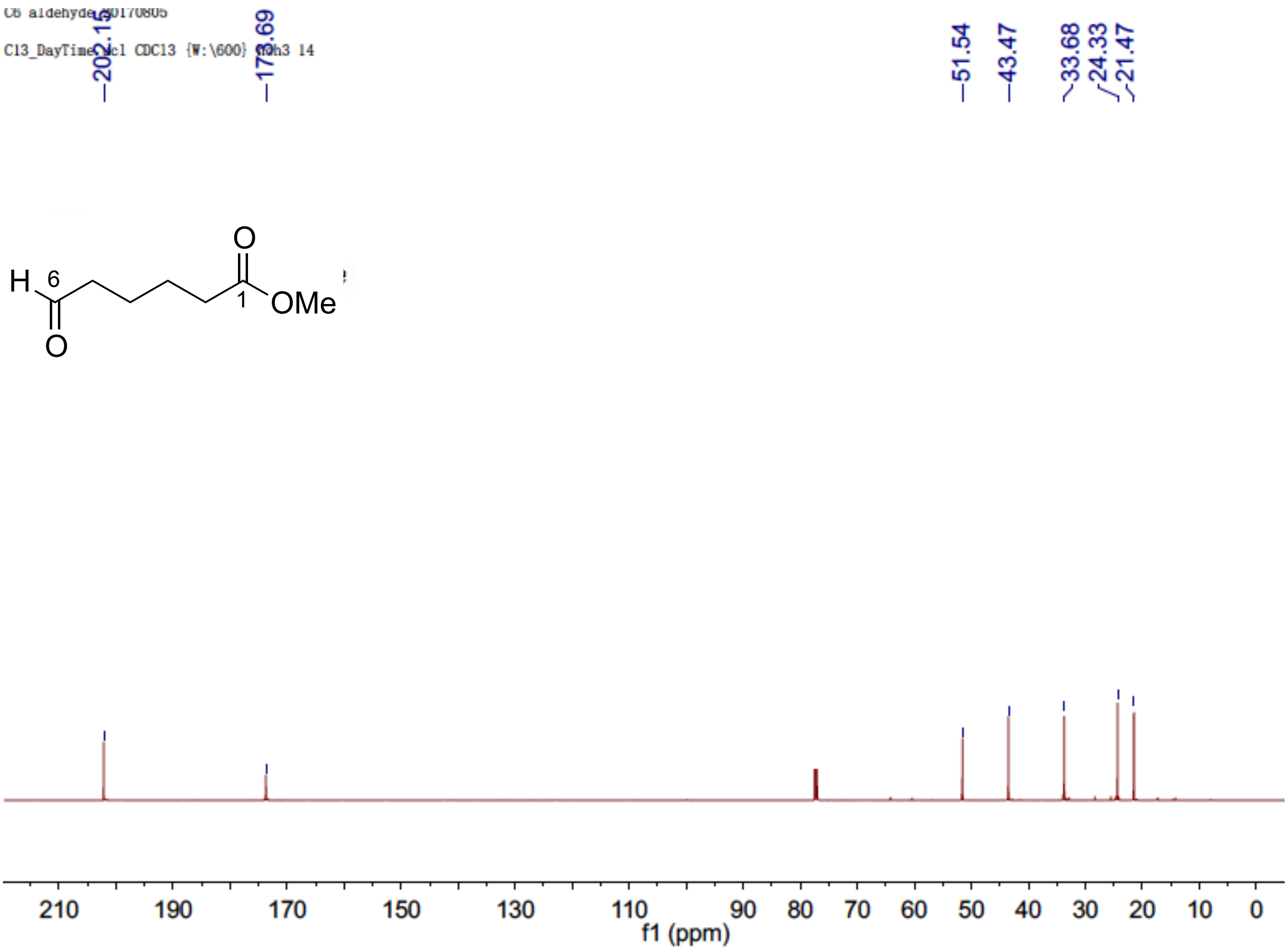

^{13}C NMR spectrum (compound **10**)

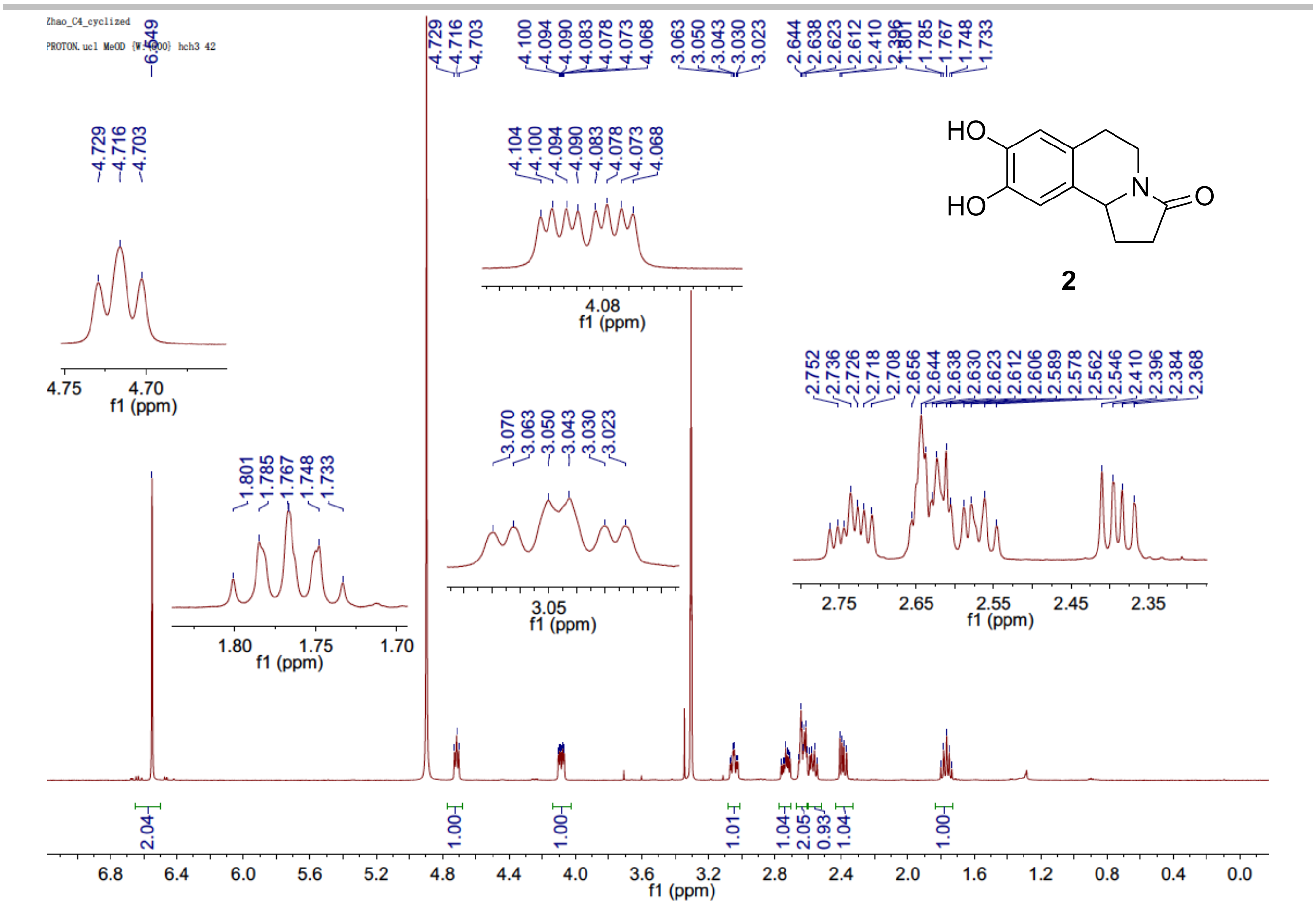

^{1}H NMR spectrum (compound **2**)

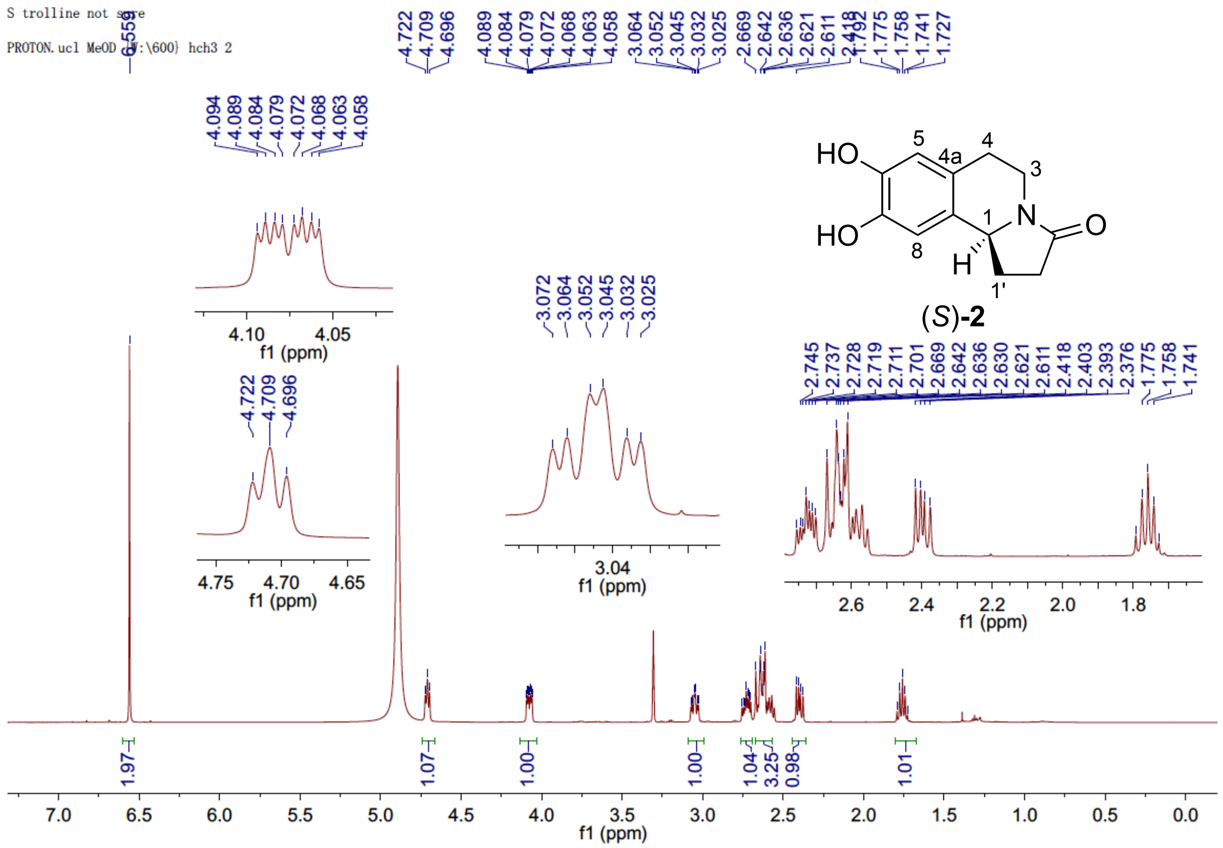

^{1}H NMR spectrum (compound (*S*)-**2**)

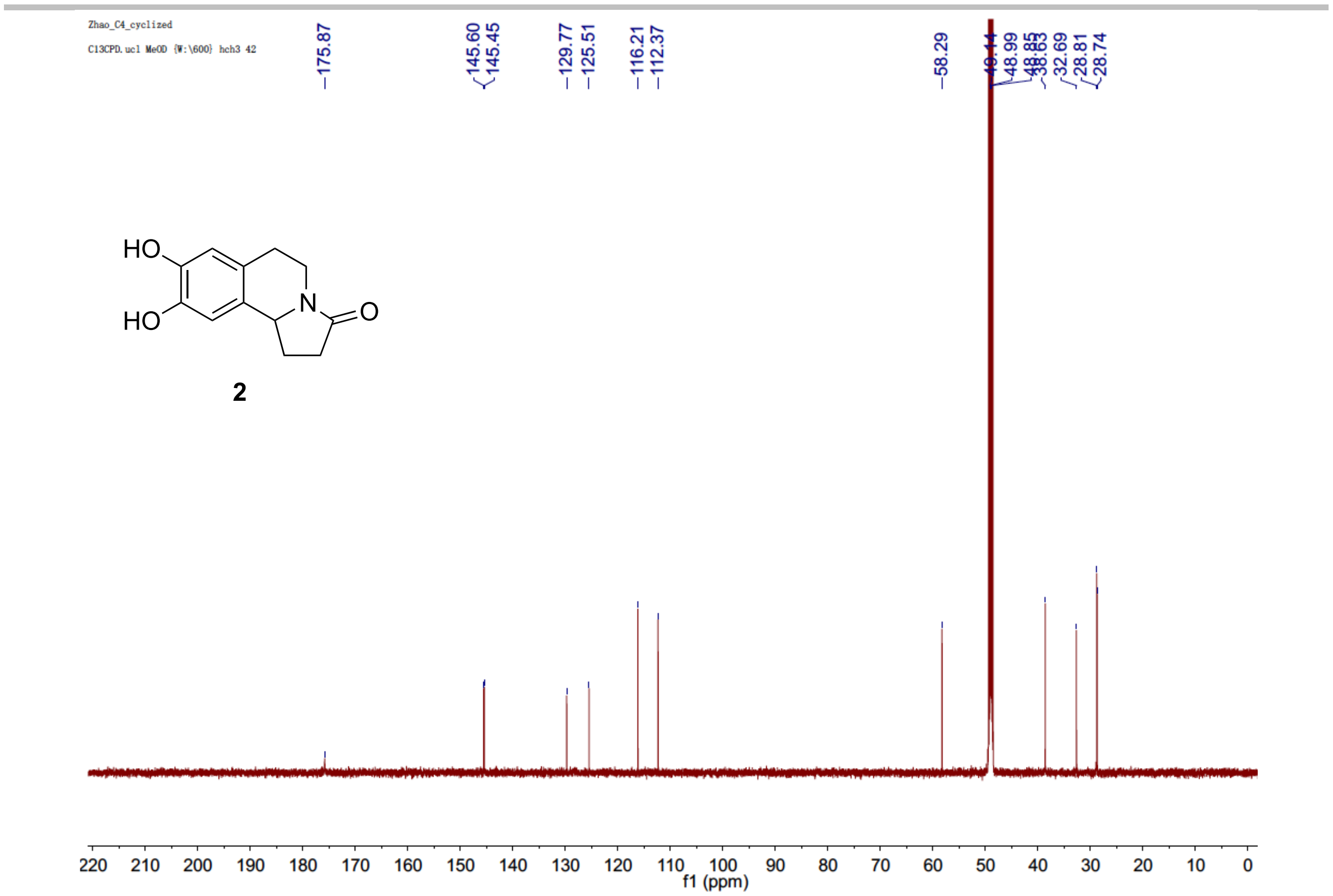

^{13}C NMR spectrum (compound **2**)

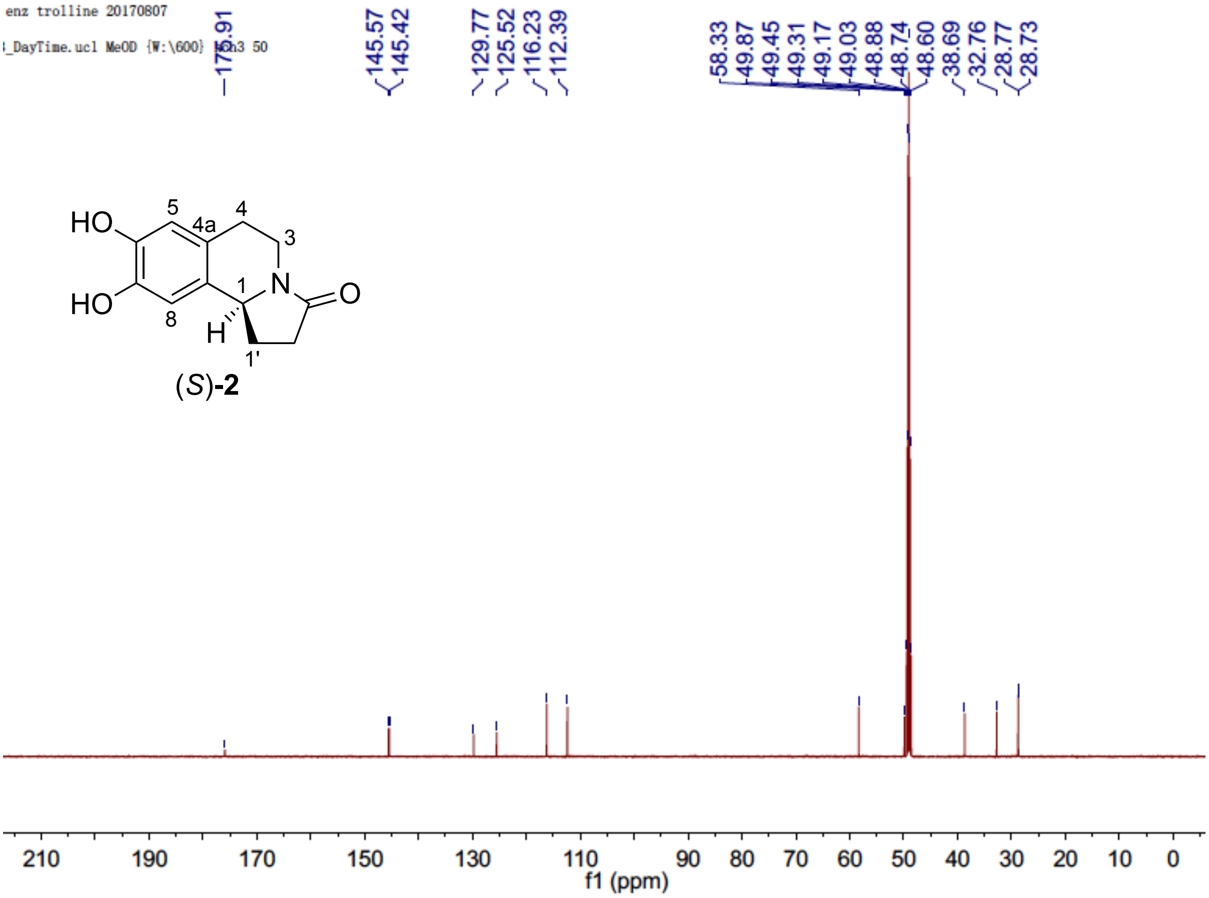

^{13}C NMR spectrum (compound (*S*)-**2**)

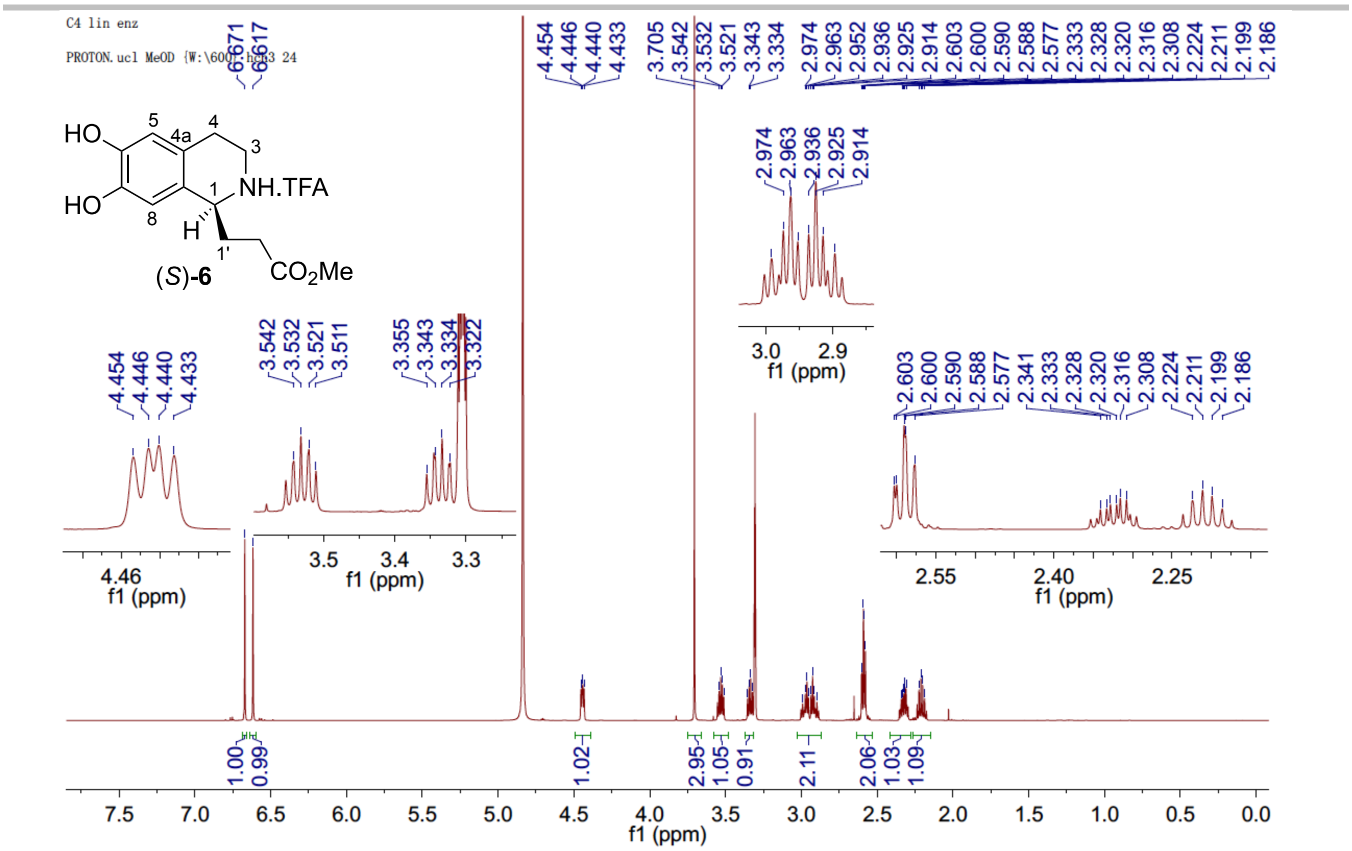

^{1}H NMR spectrum (compound (*S*)-**6**.TFA)

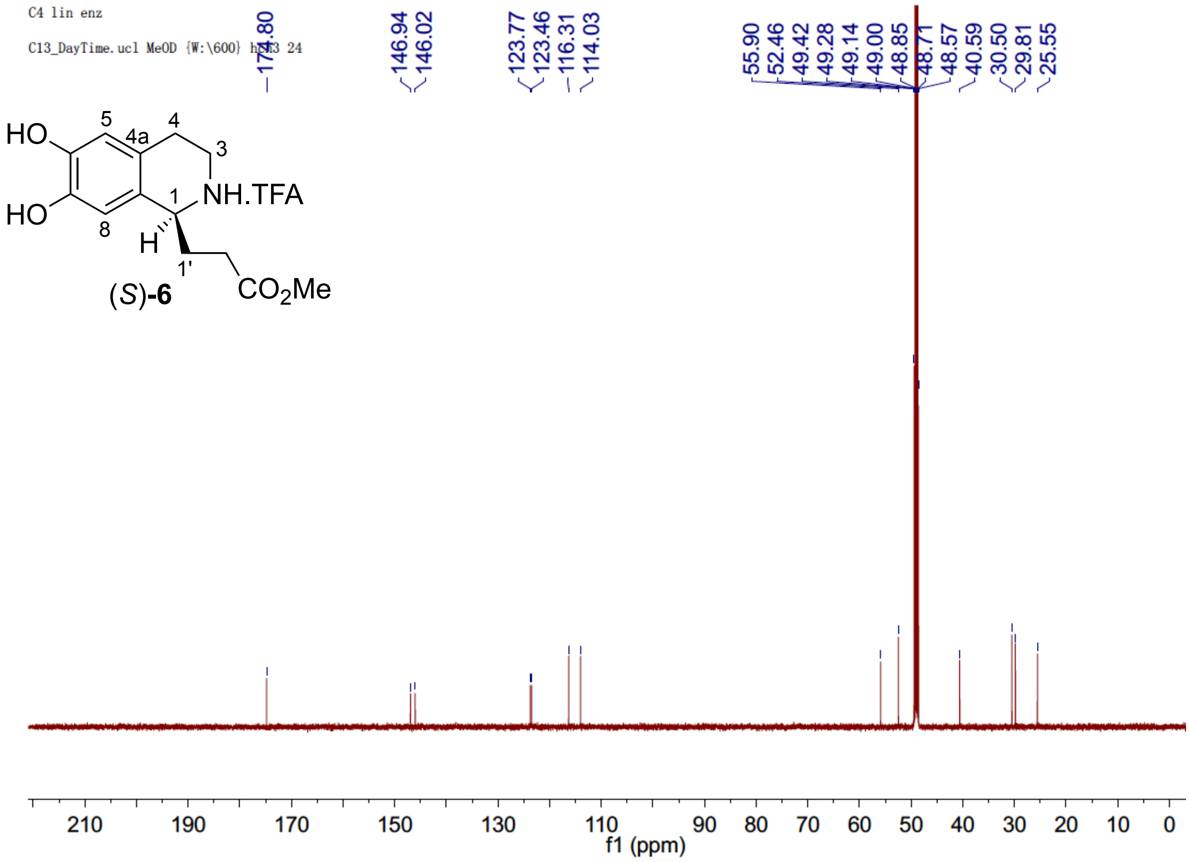

^{13}C NMR spectrum (compound (*S*)-**6**.TFA)

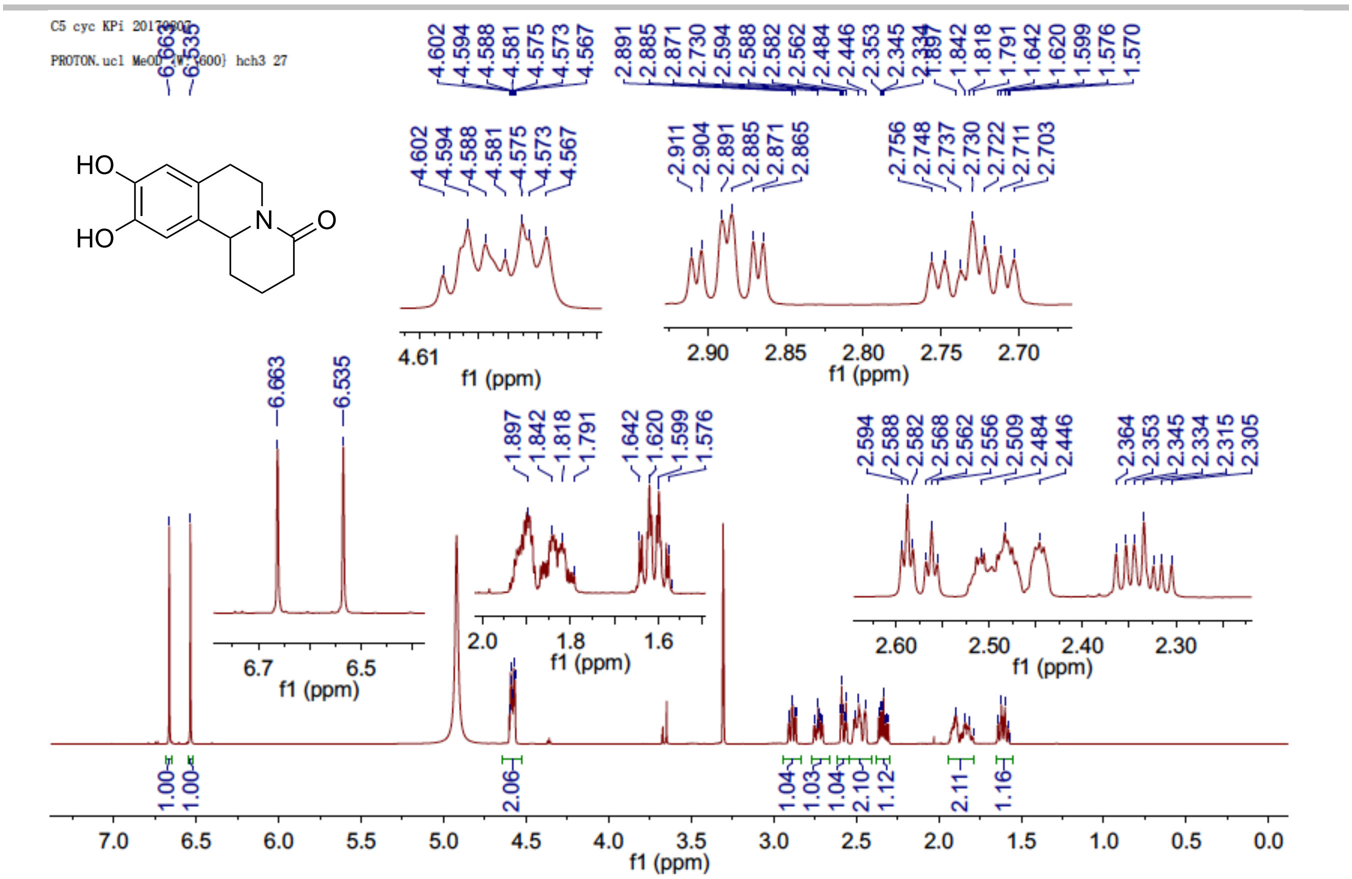

^{1}H NMR spectrum (compound **9**)

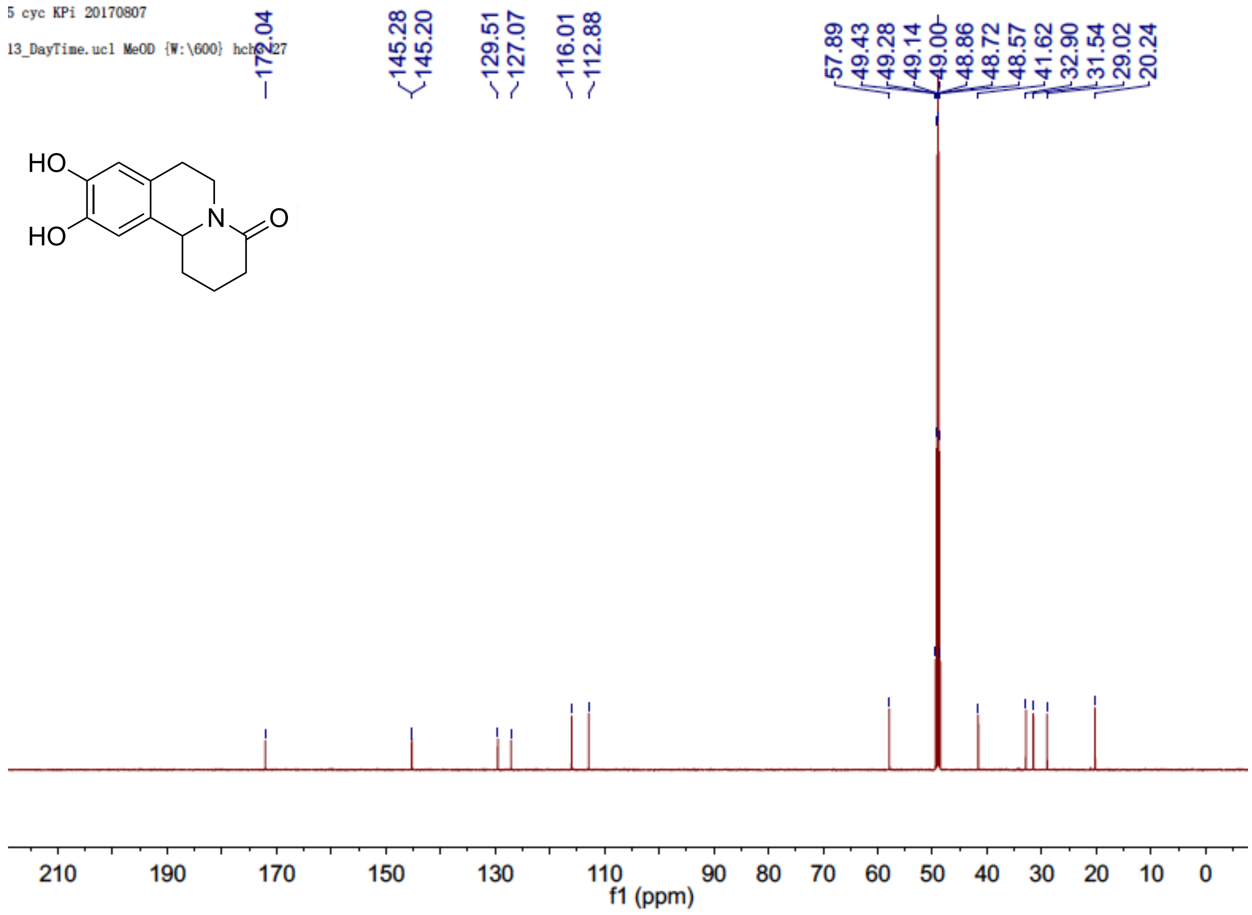

^{13}C NMR spectrum (compound **9**)

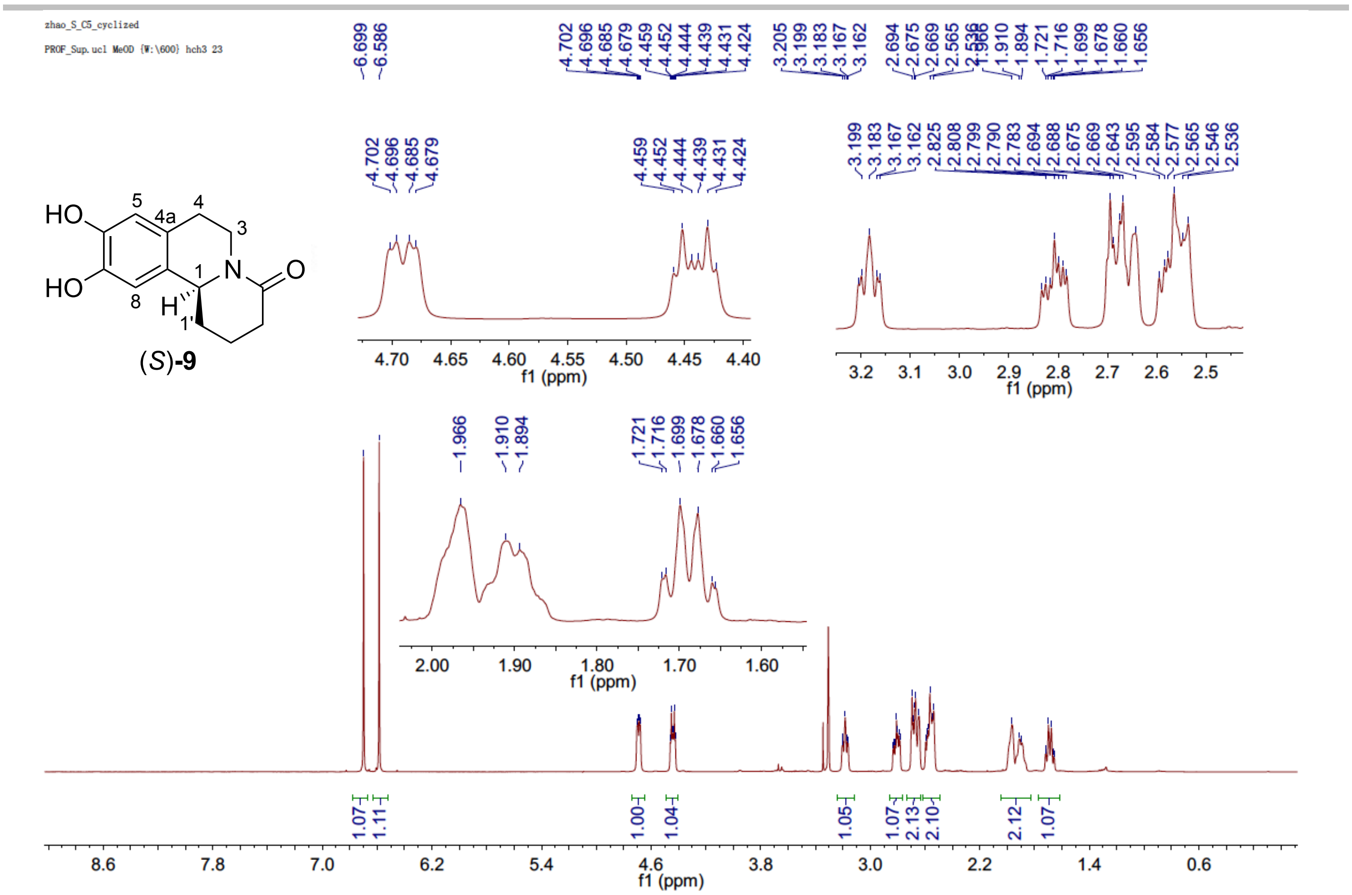

^{1}H NMR spectrum (compound (*S*)-**9**) using water suppression

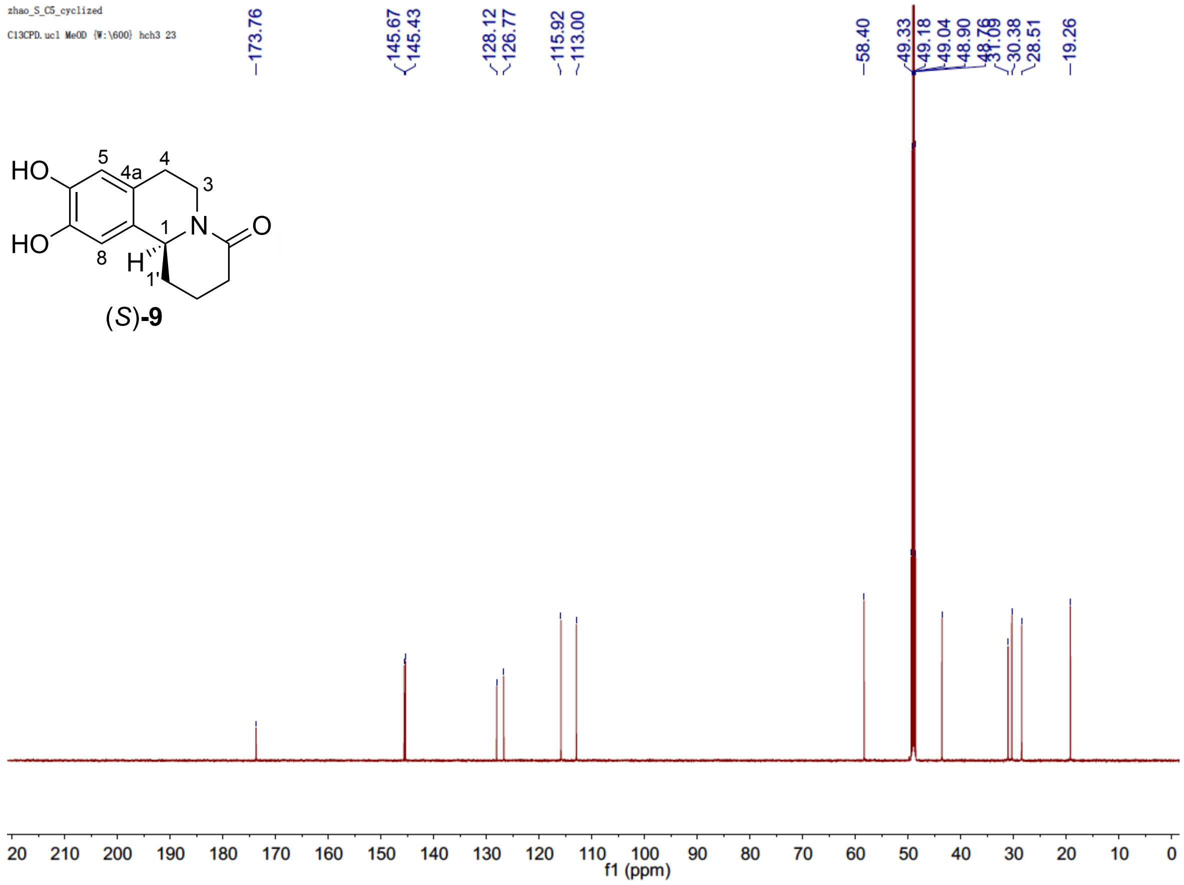

^{13}C NMR spectrum (compound (*S*)-**9**)

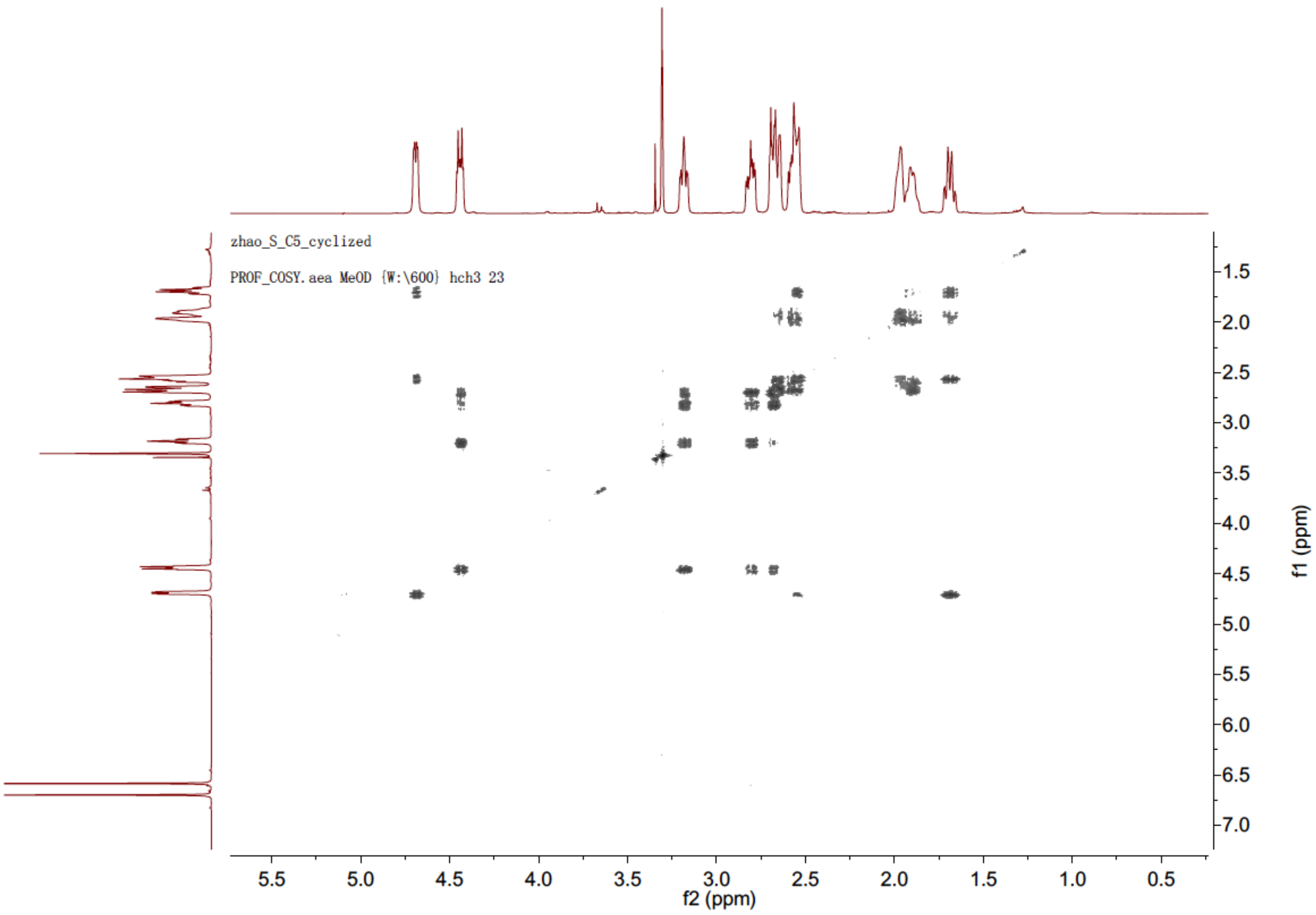

COSY NMR spectrum (compound (*S*)-**9**)

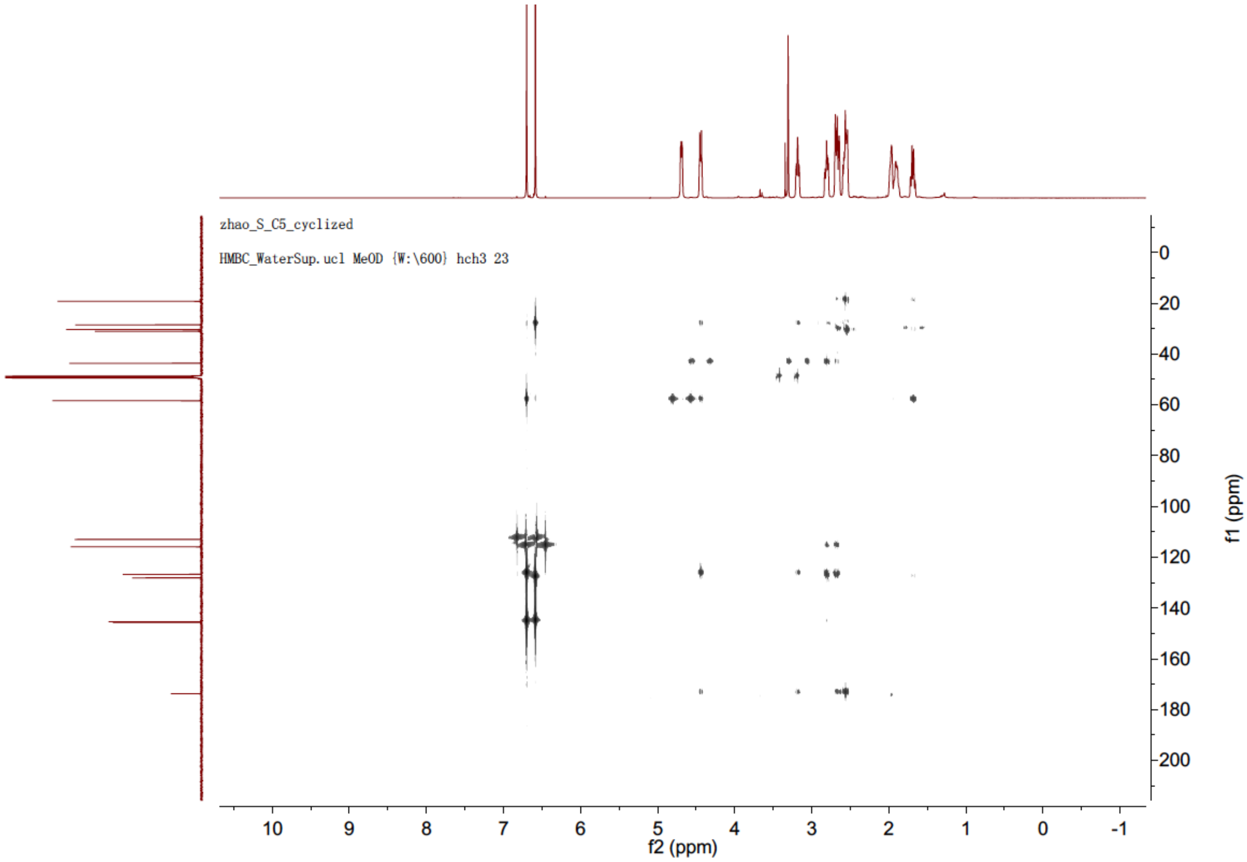

HMBC NMR spectrum (compound (*S*)-**9**)

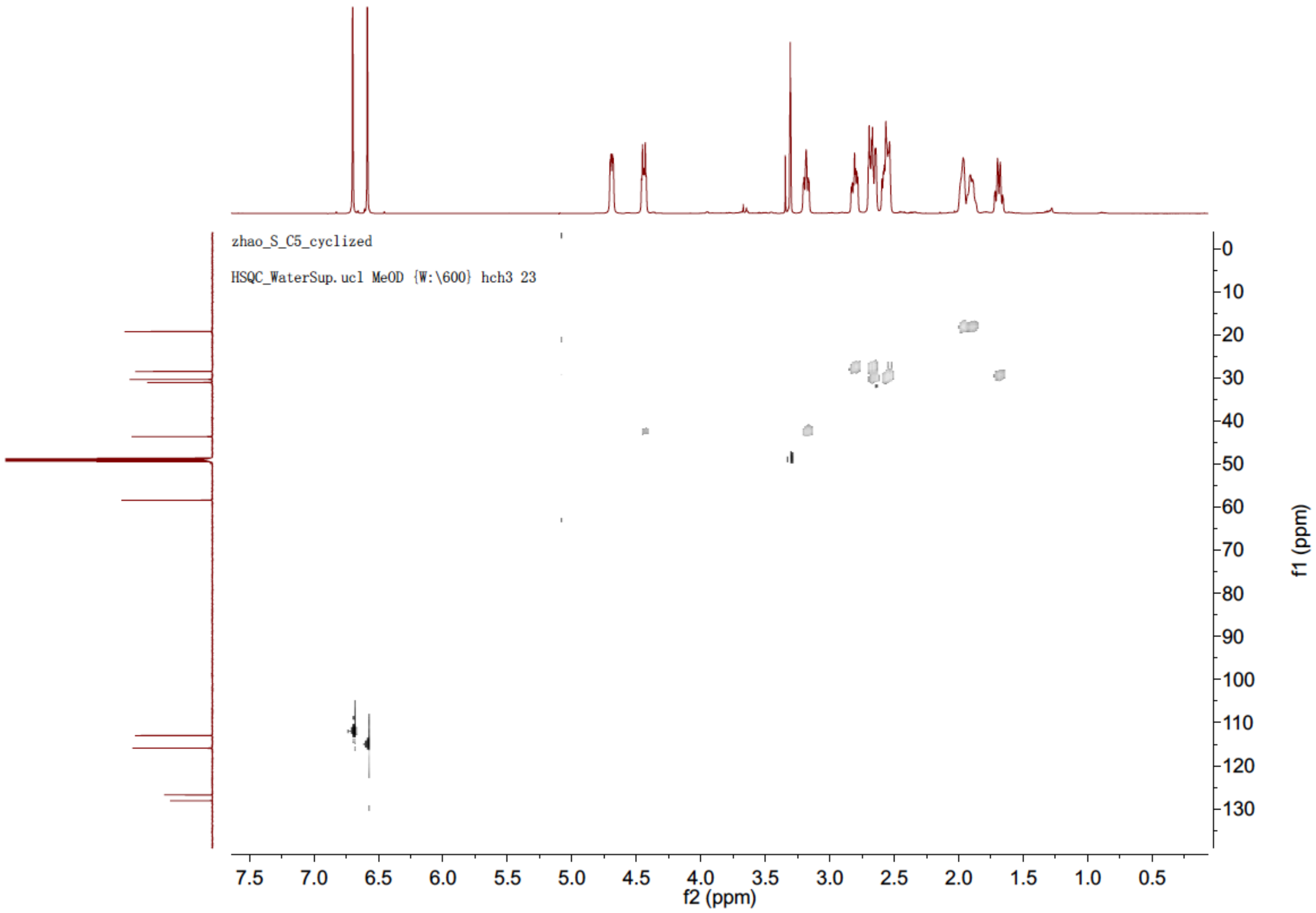

HSQC NMR spectrum (compound (*S*)-**9**)

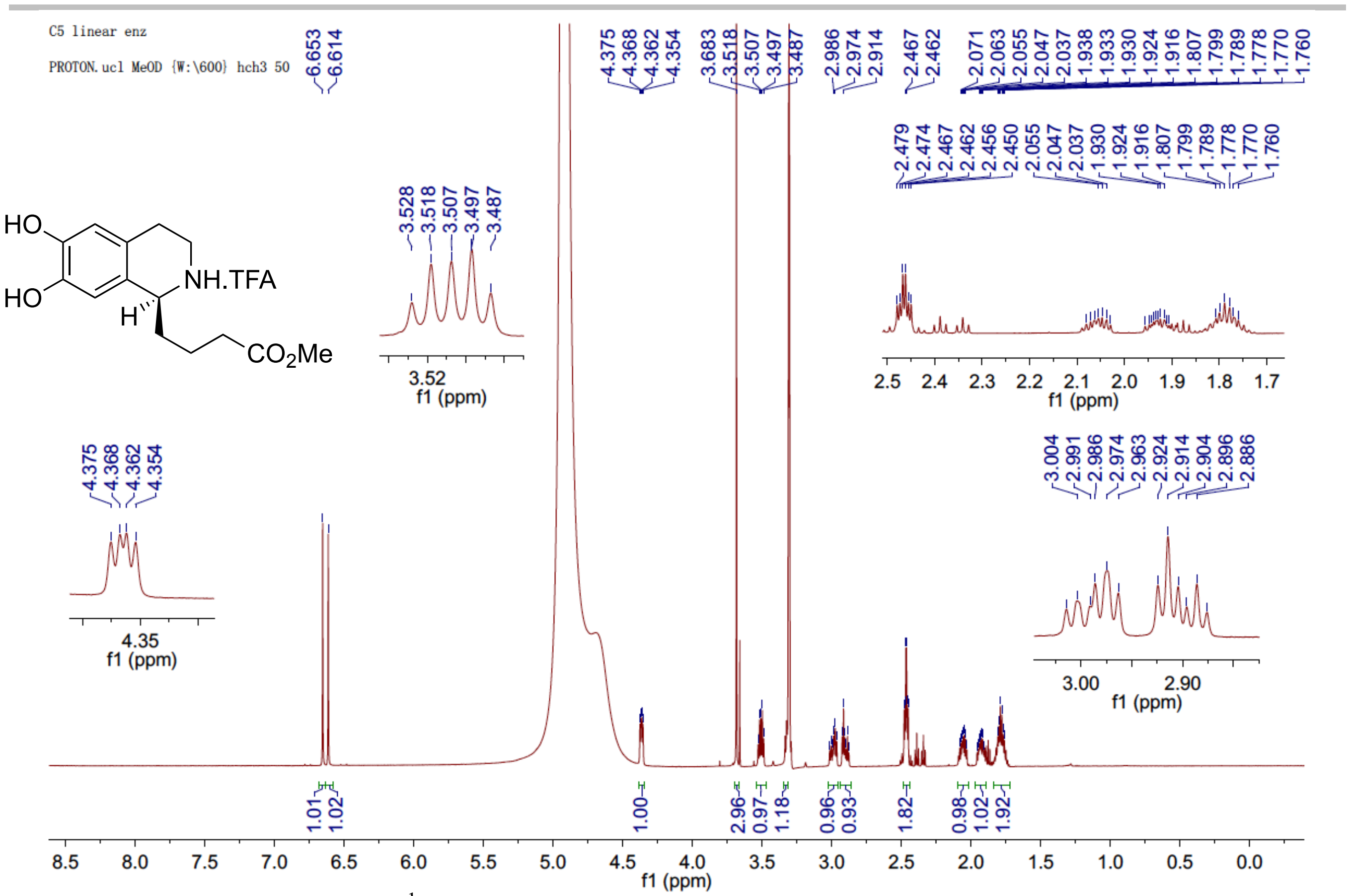

^{1}H NMR spectrum (compound (*S*)-**8**.TFA)

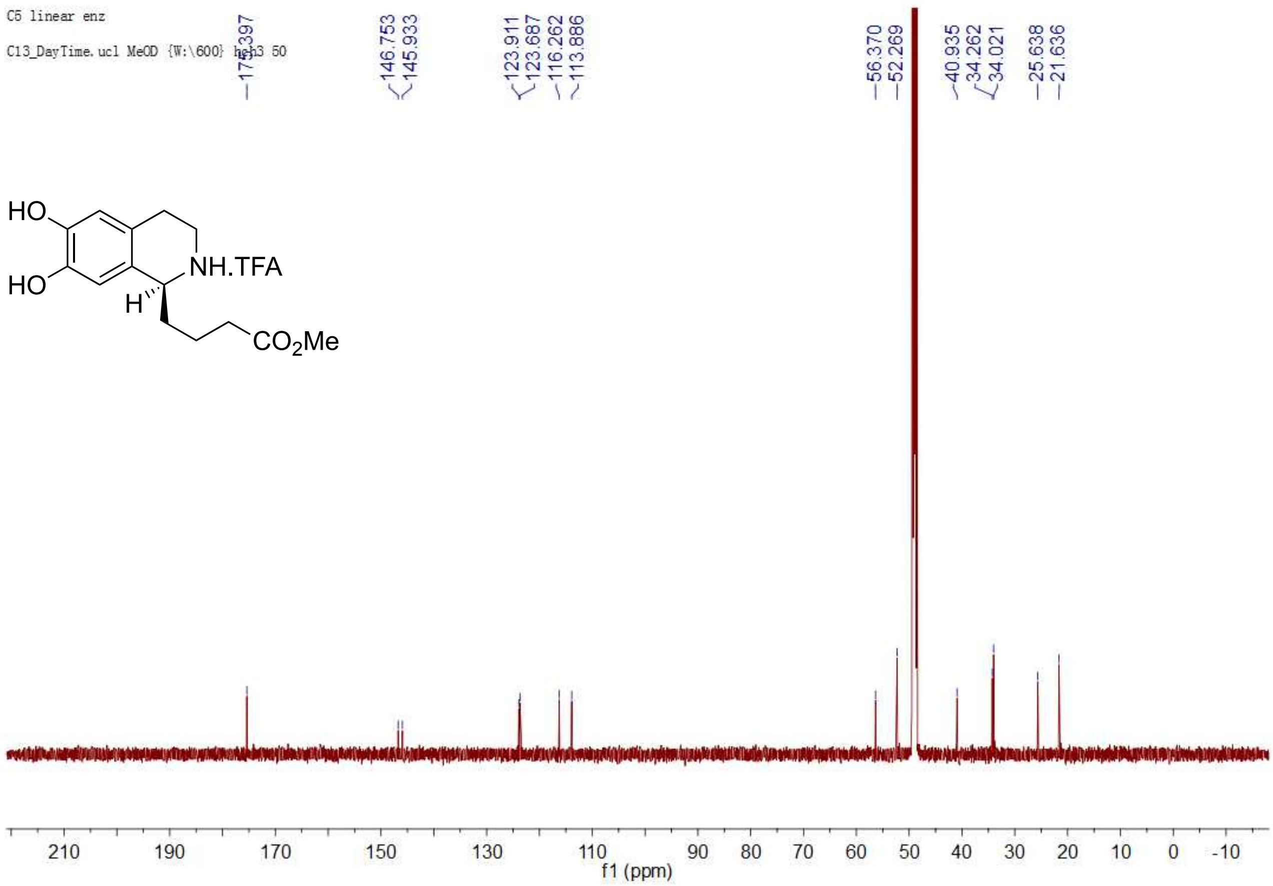

^{13}C NMR spectrum (compound (*S*)-**8**.TFA)

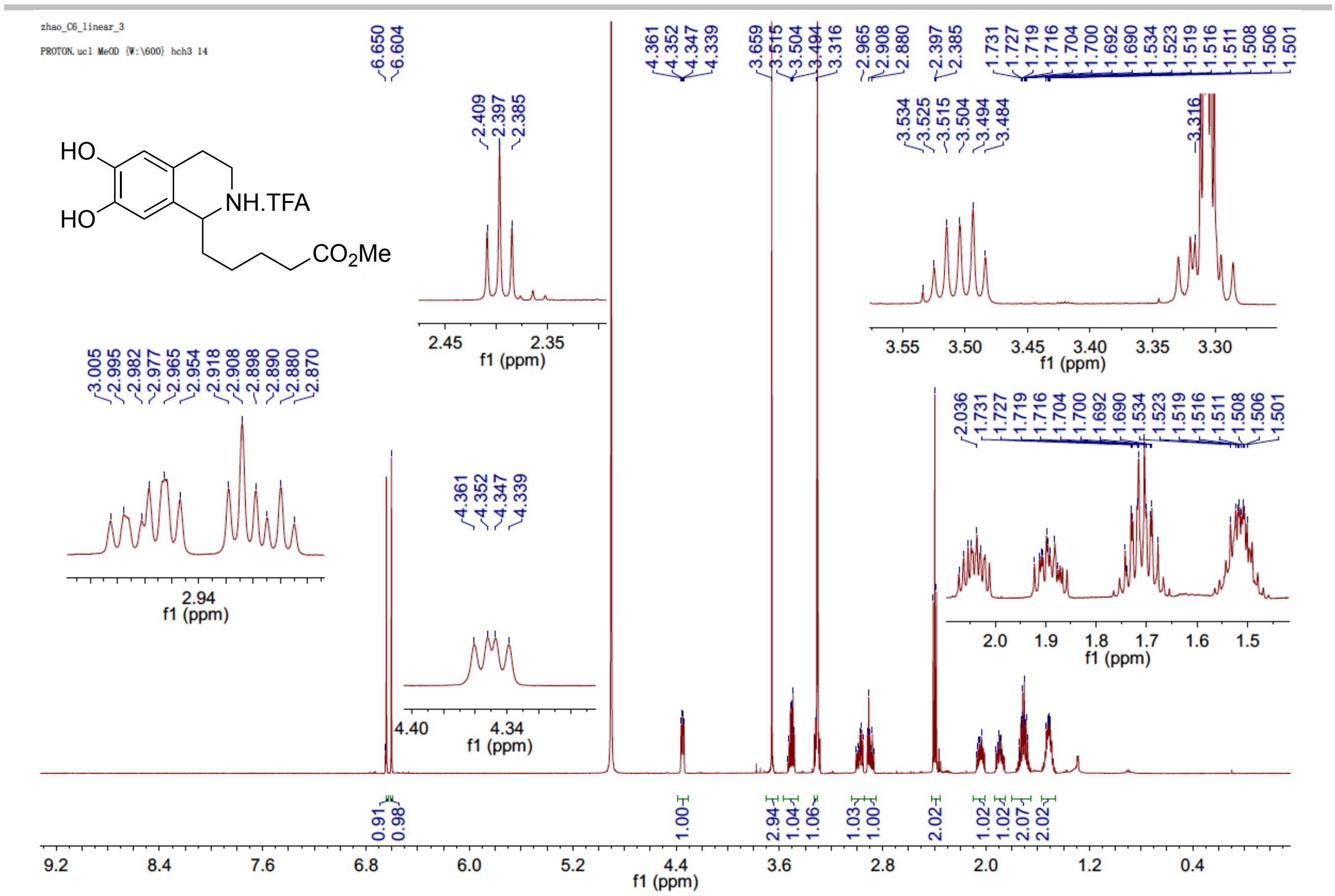

^{1}H NMR spectrum (compound **11**.TFA)

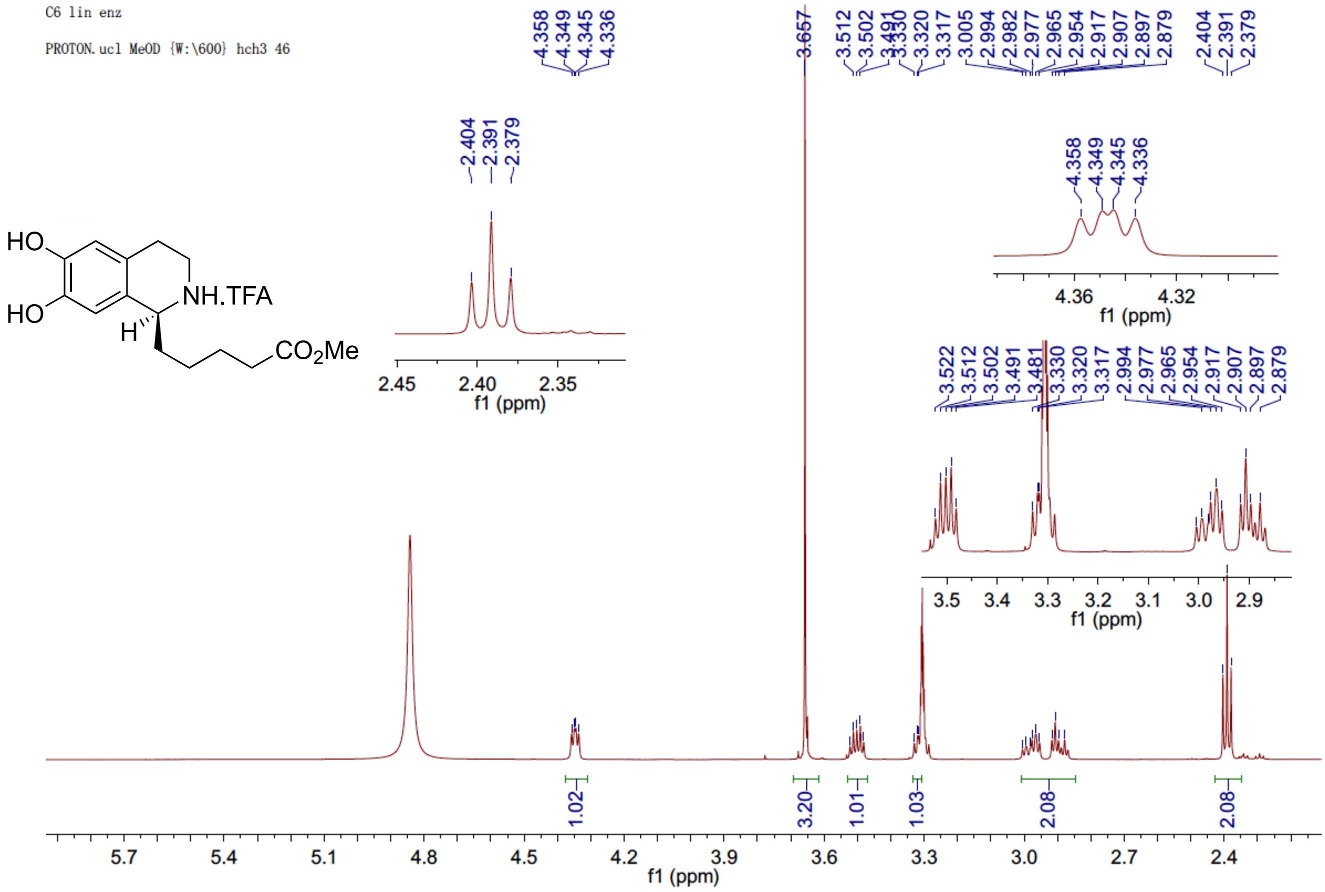

^{1}H NMR spectrum (compound (*S*)-**11**.TFA)

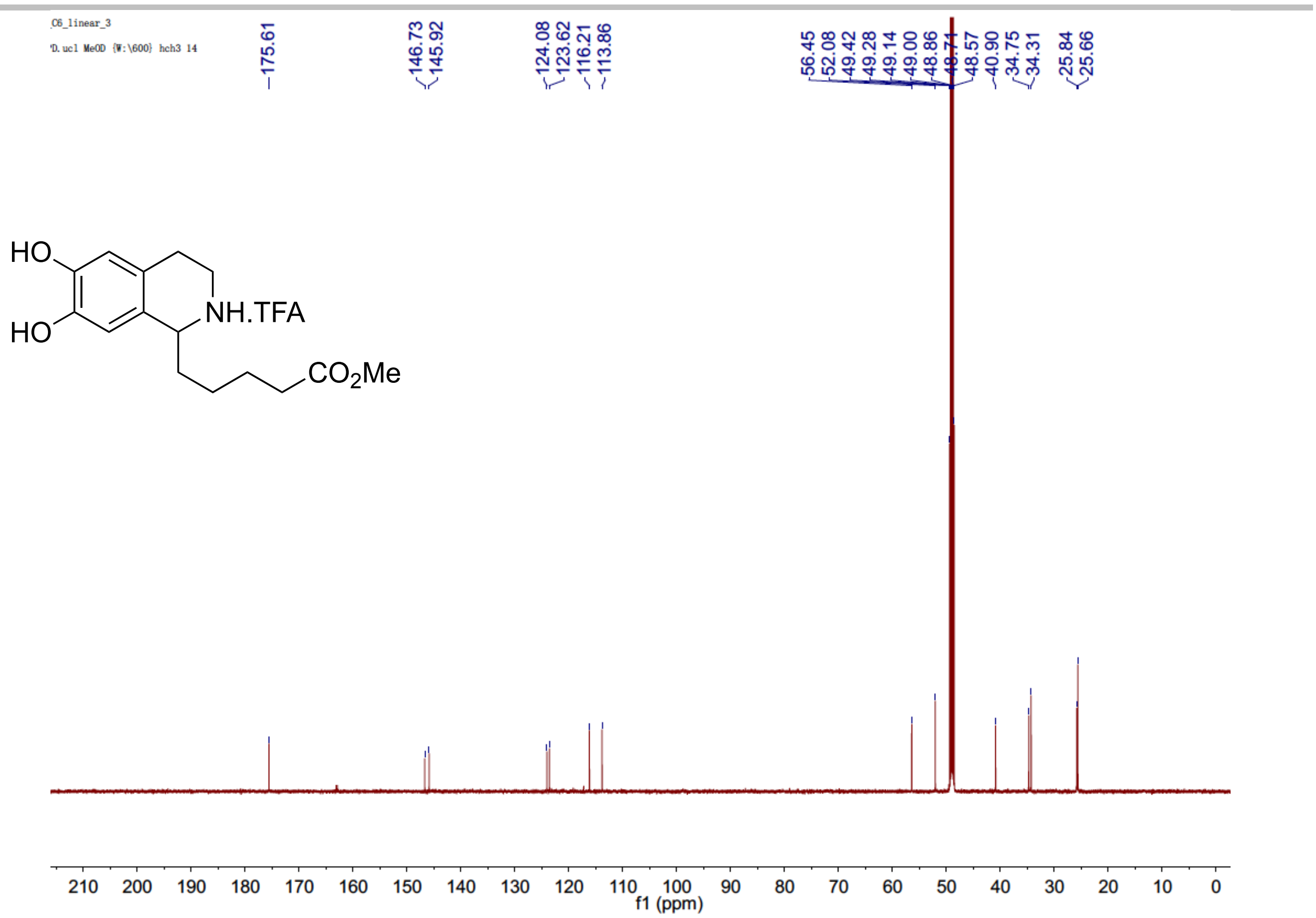

^{13}C NMR spectrum (compound **11**.TFA)

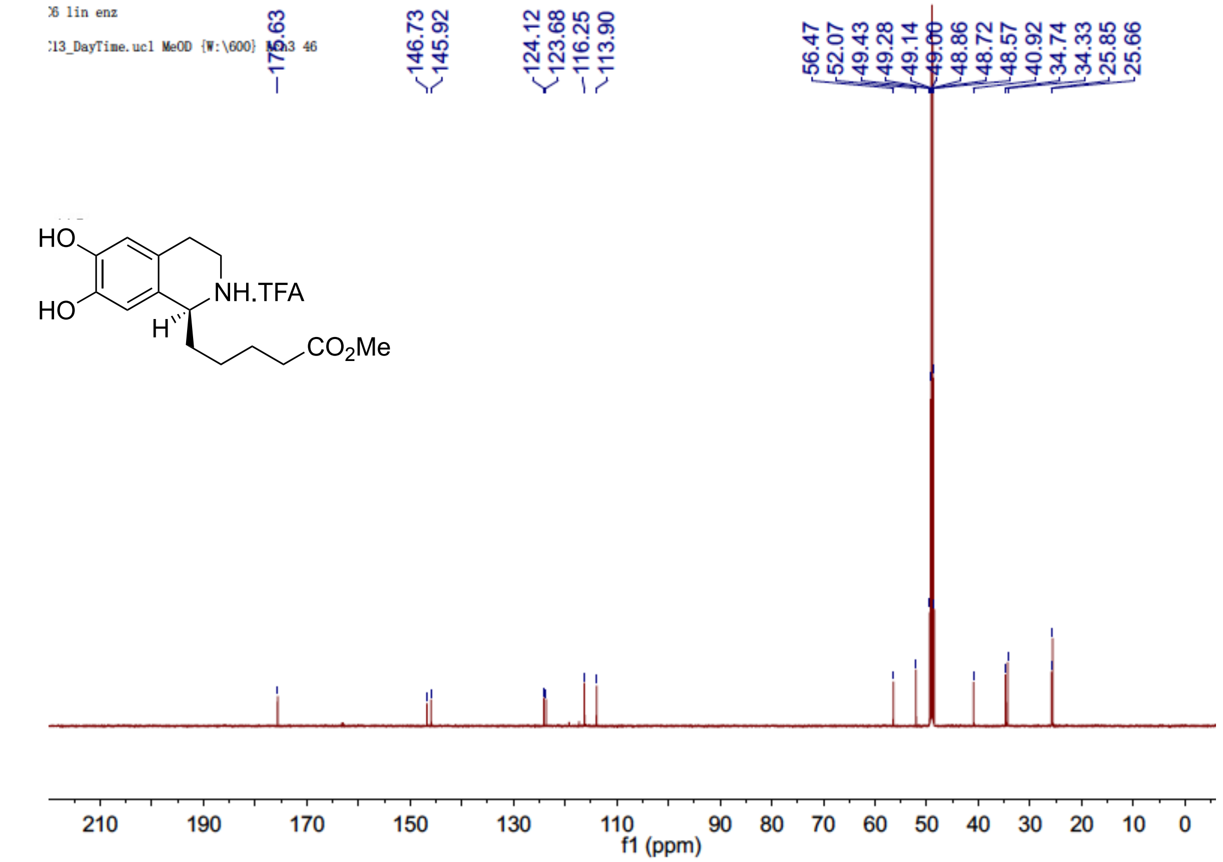

^{13}C NMR spectrum (compound (*S*)-**11**.TFA)

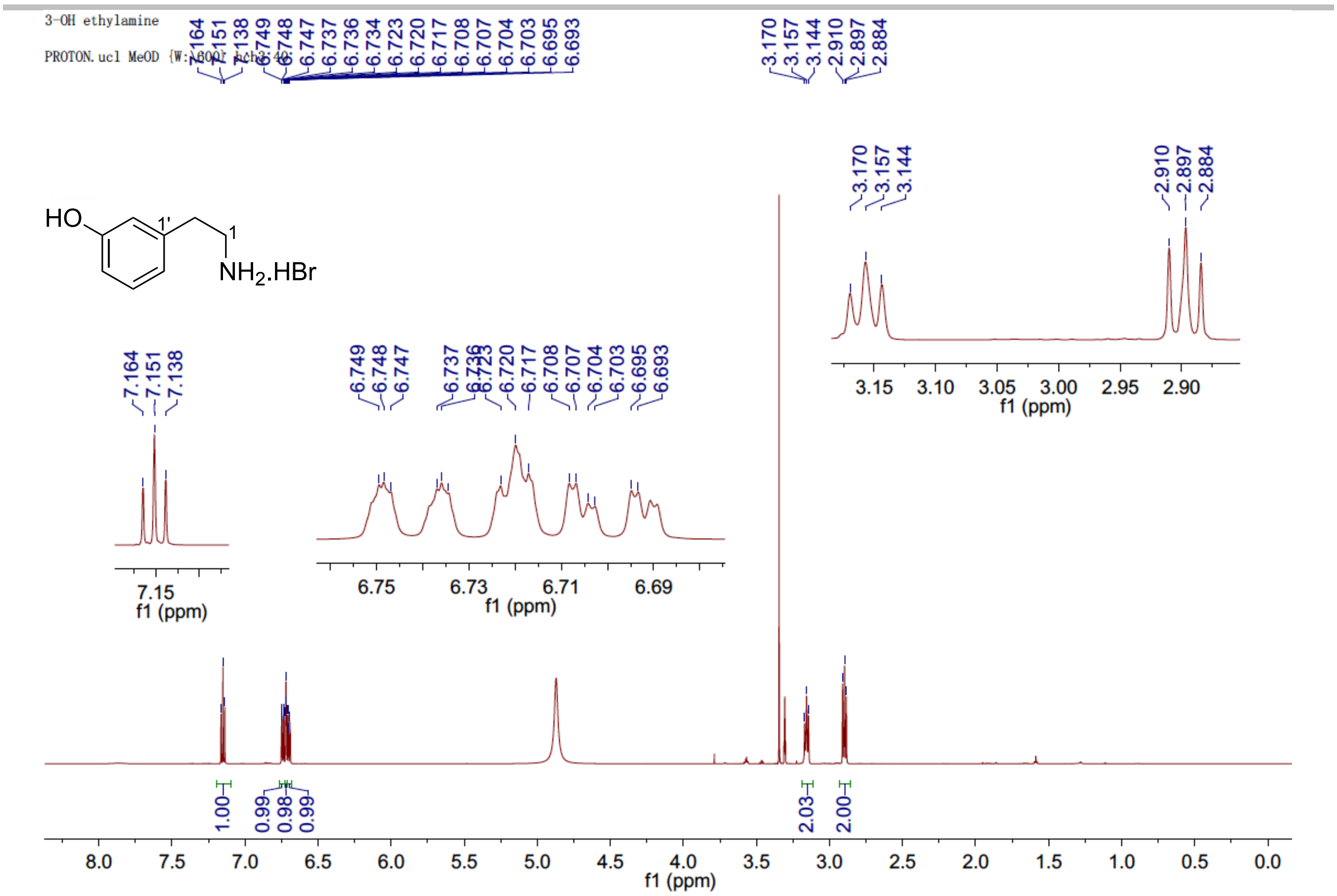

^{1}H NMR spectrum (compound **13**.HBr)

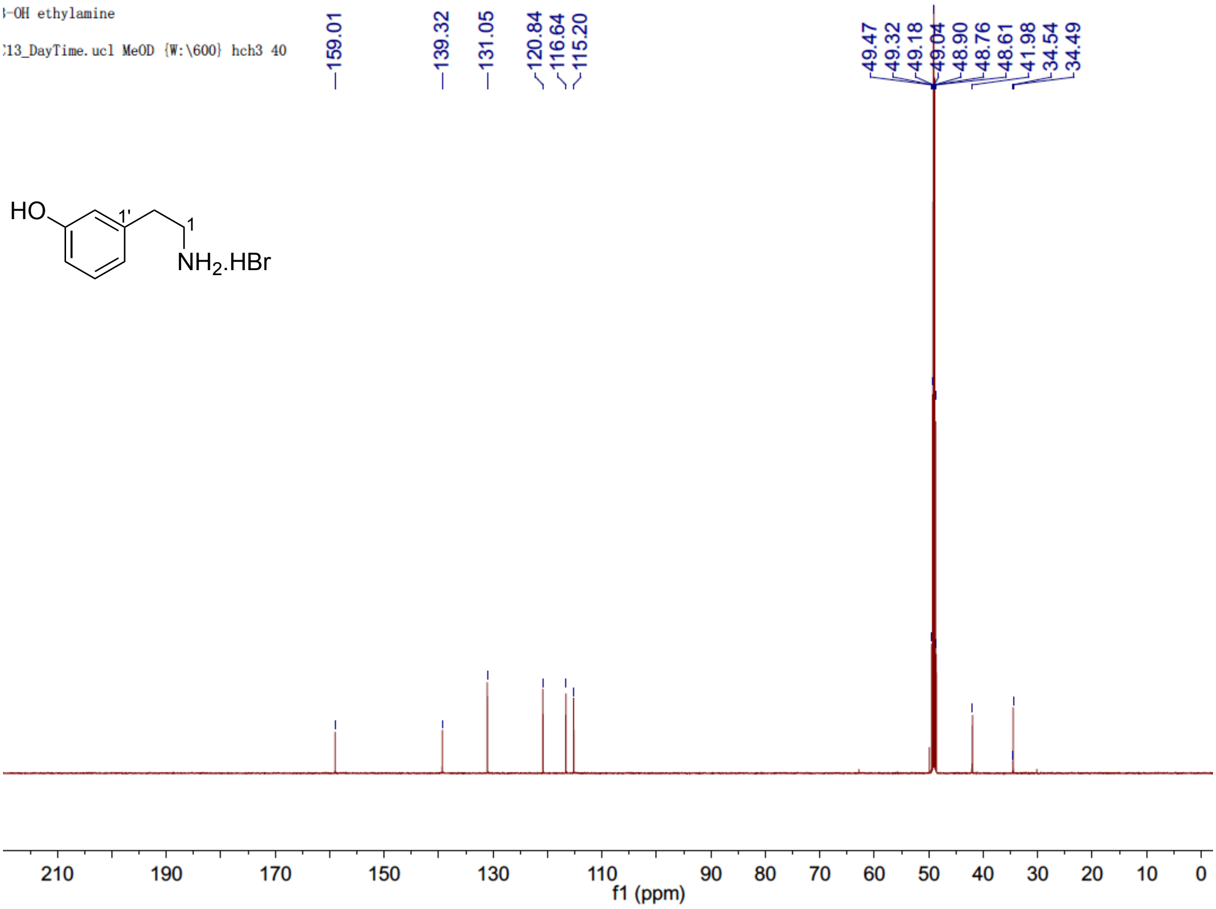

^{13}C NMR spectrum (compound **13**.HBr)

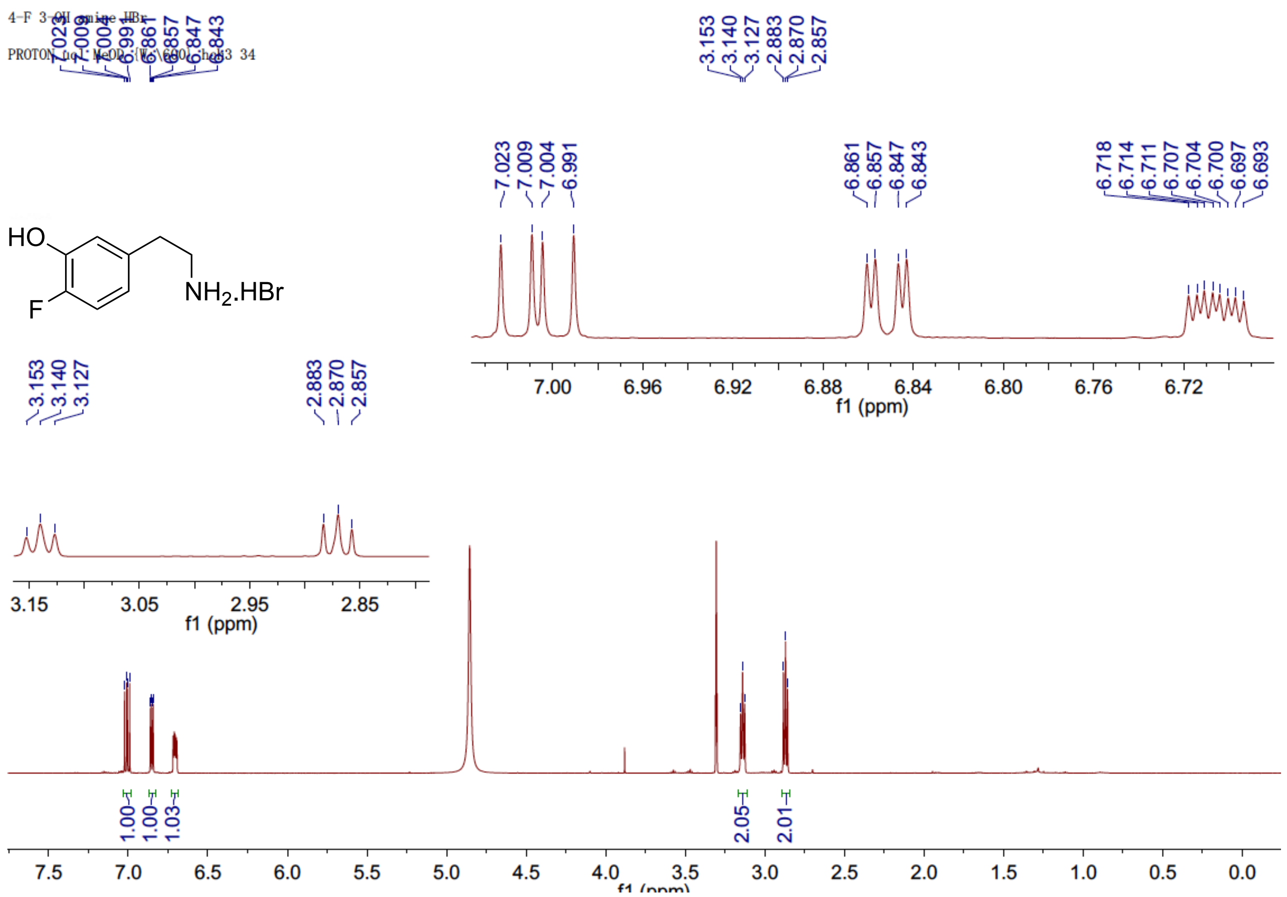

^{1}H NMR spectroscopy (compound **15**.HBr)

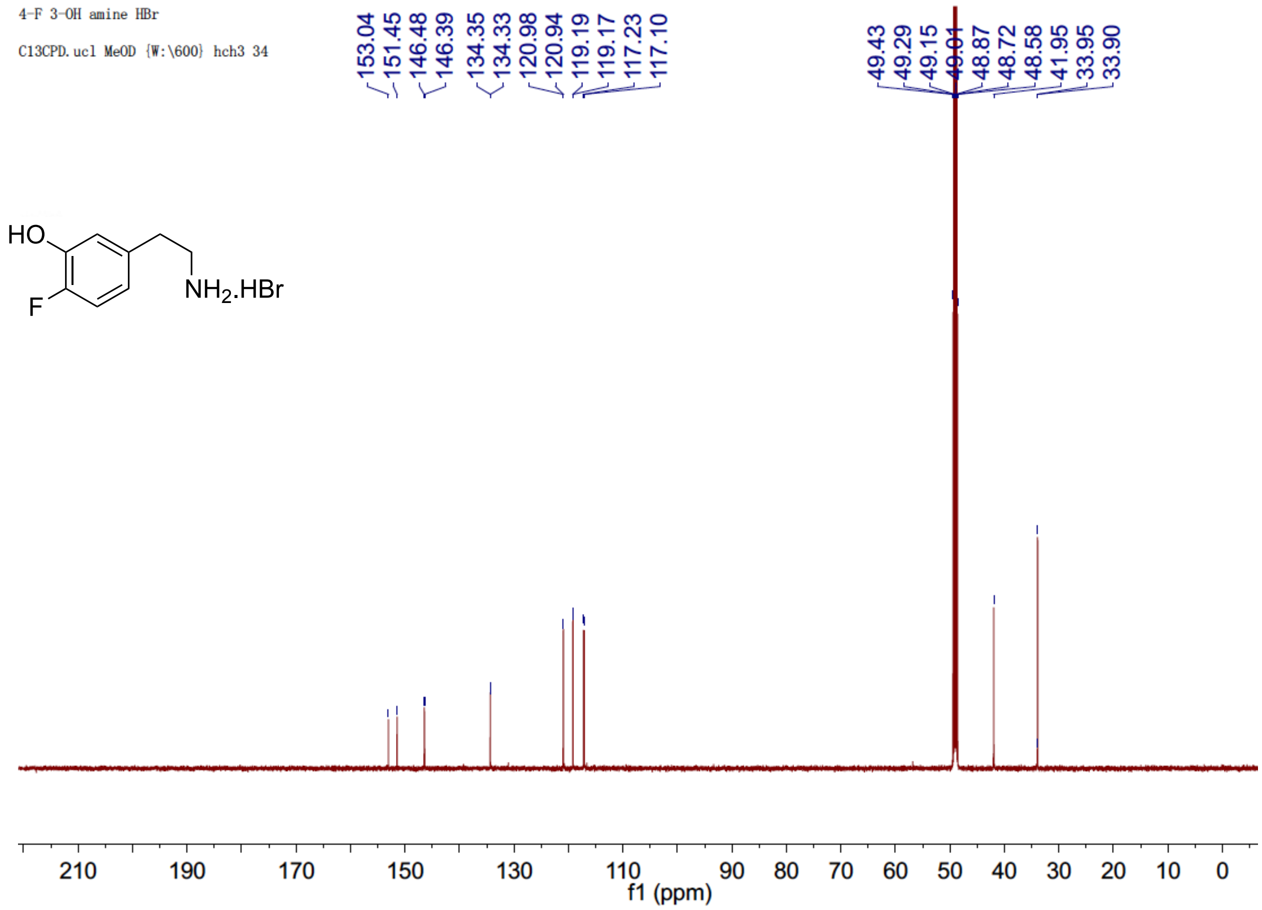

^{13}C NMR spectrum (compound **15**.HBr)

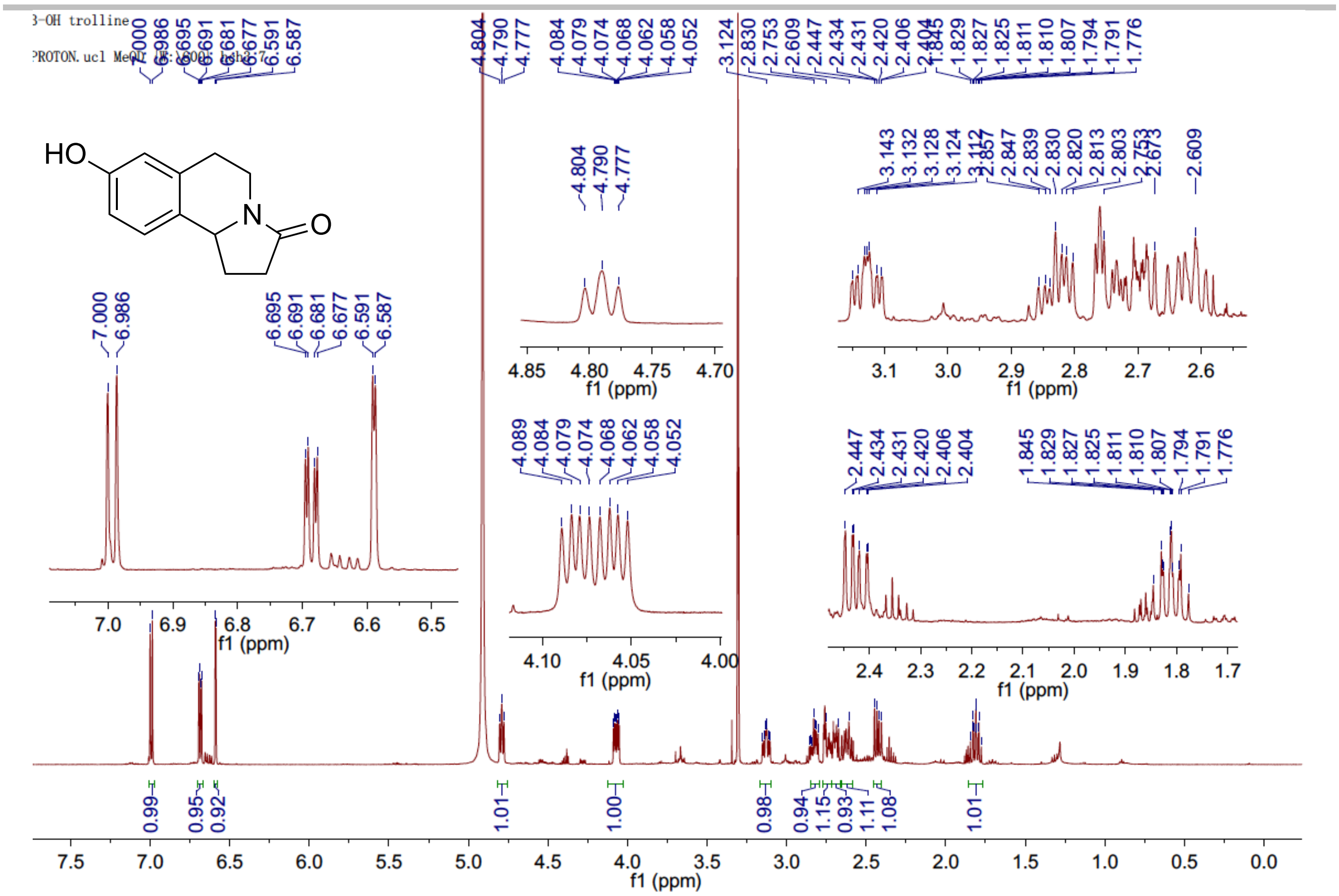

^{1}H NMR spectrum (compound **16**)

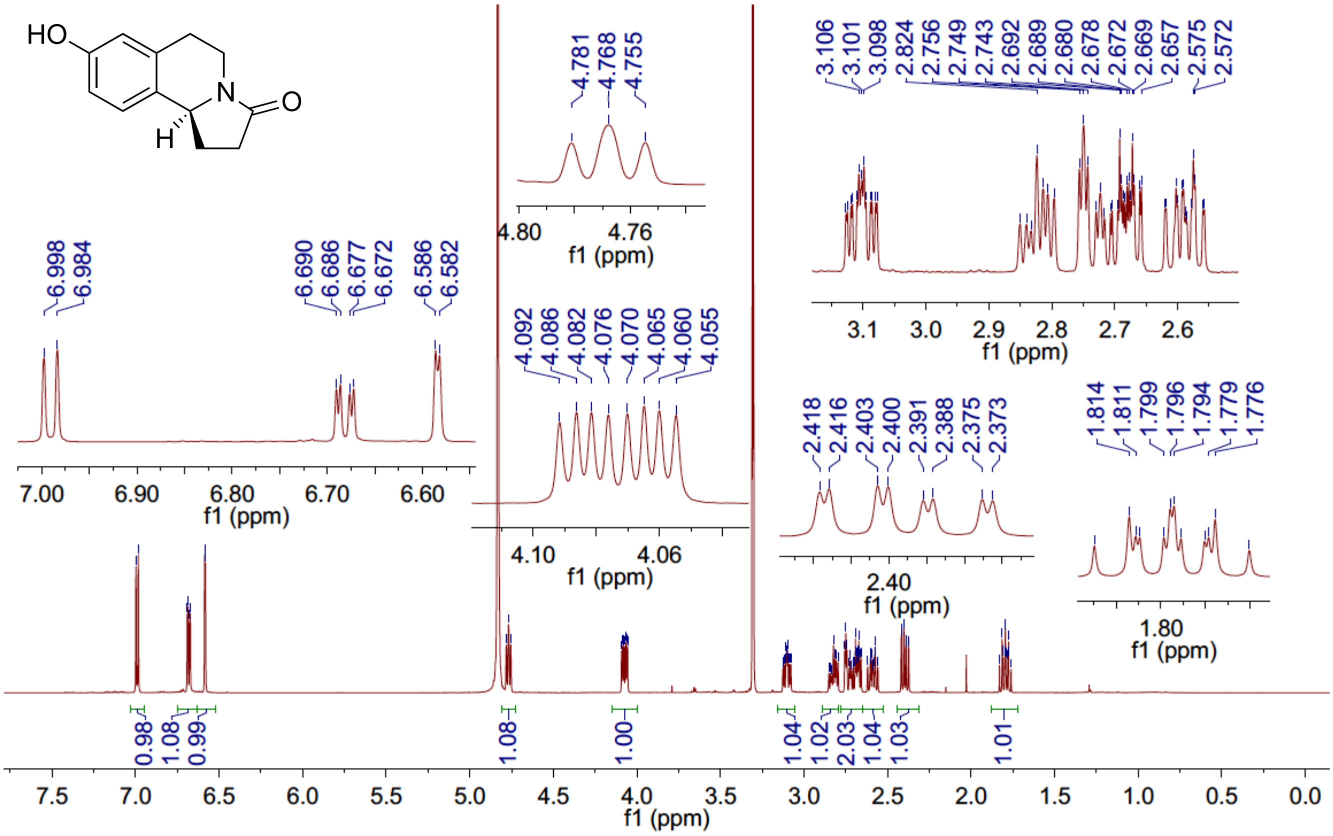

^{1}H NMR spectrum (compound (*S*)-**16**)

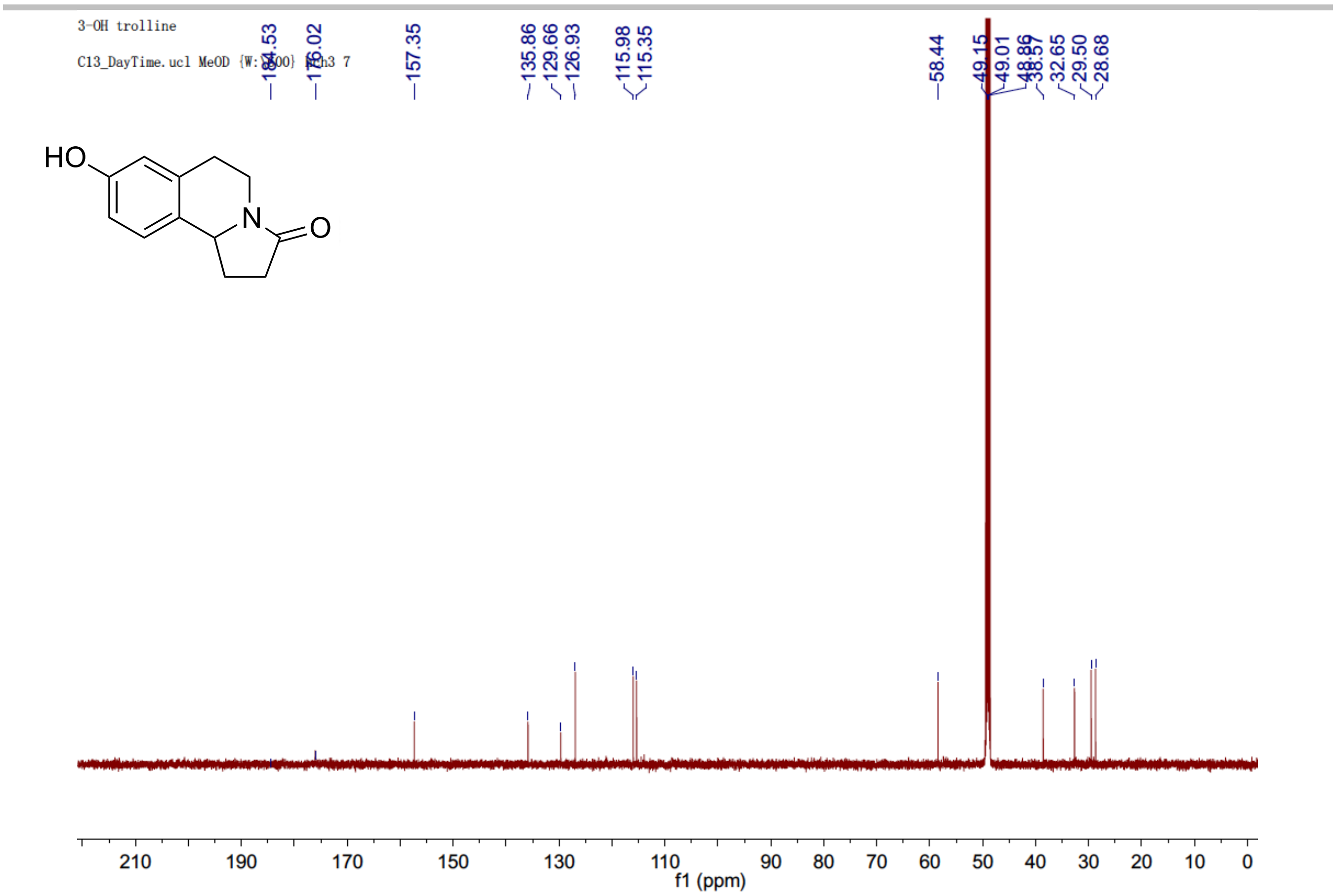

^{13}C NMR spectrum (compound **16**)

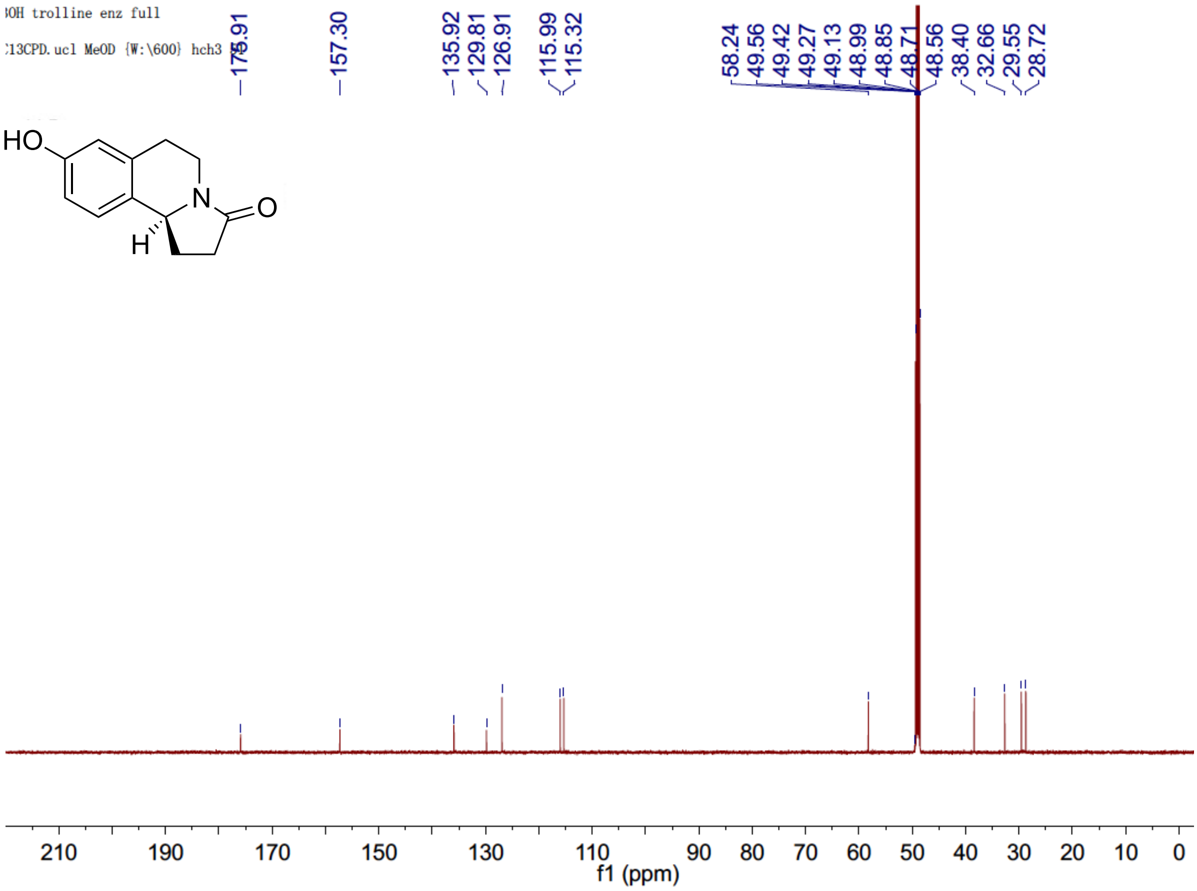

^{13}C NMR spectrum (compound (*S*)-**16**)

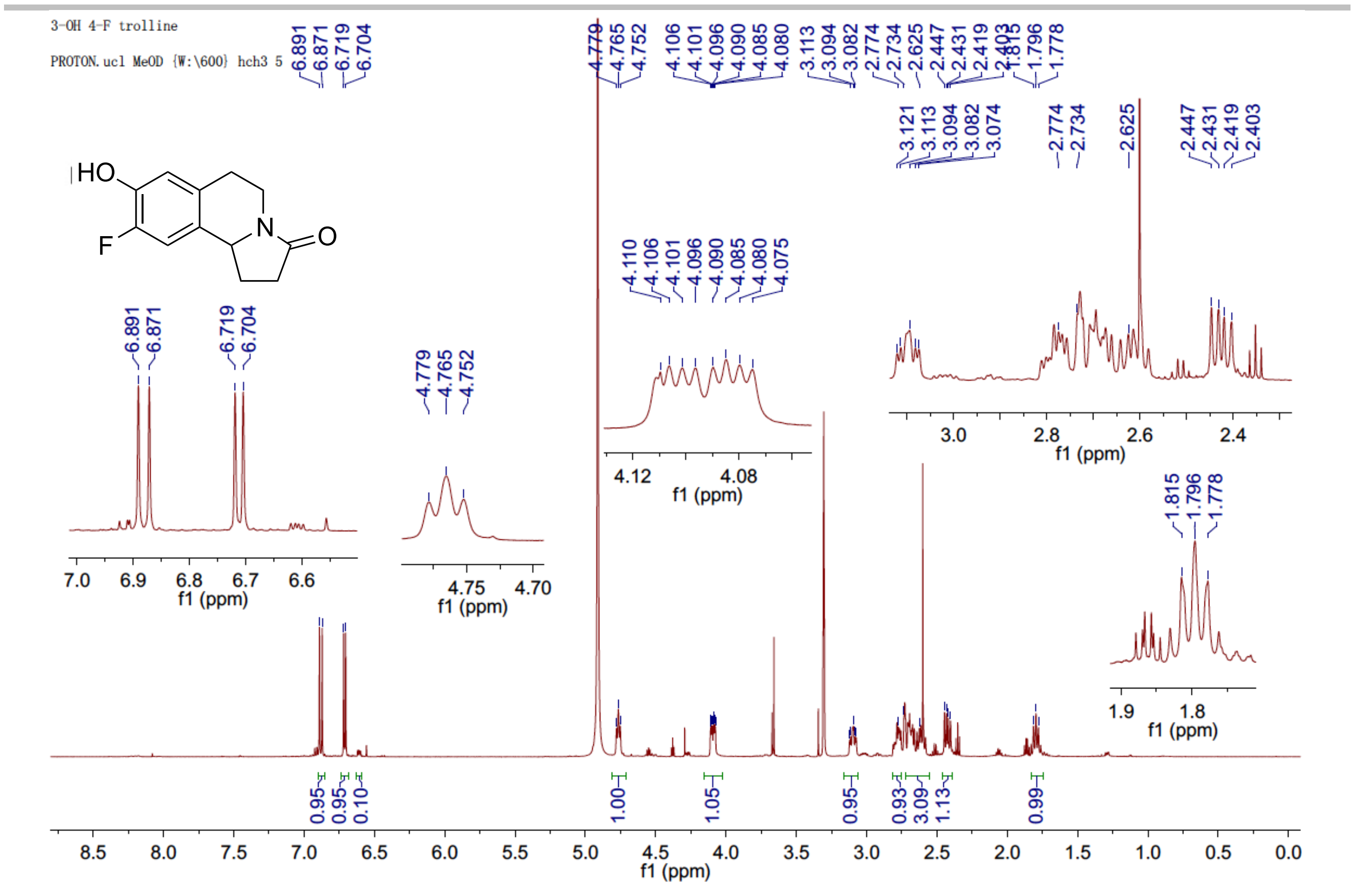

^{1}H NMR spectrum (compound **18**)

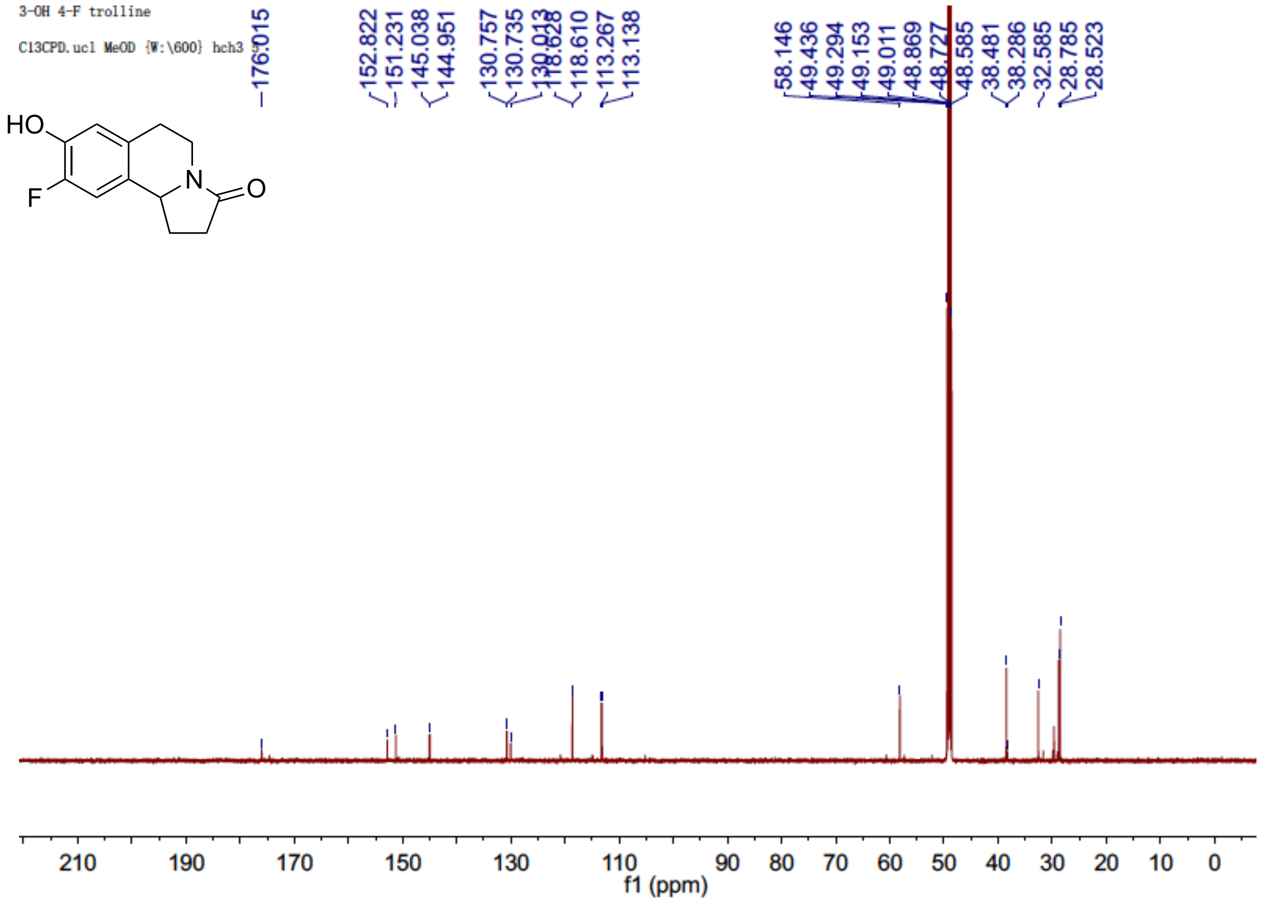

^{13}C NMR spectrum (compound **18**)

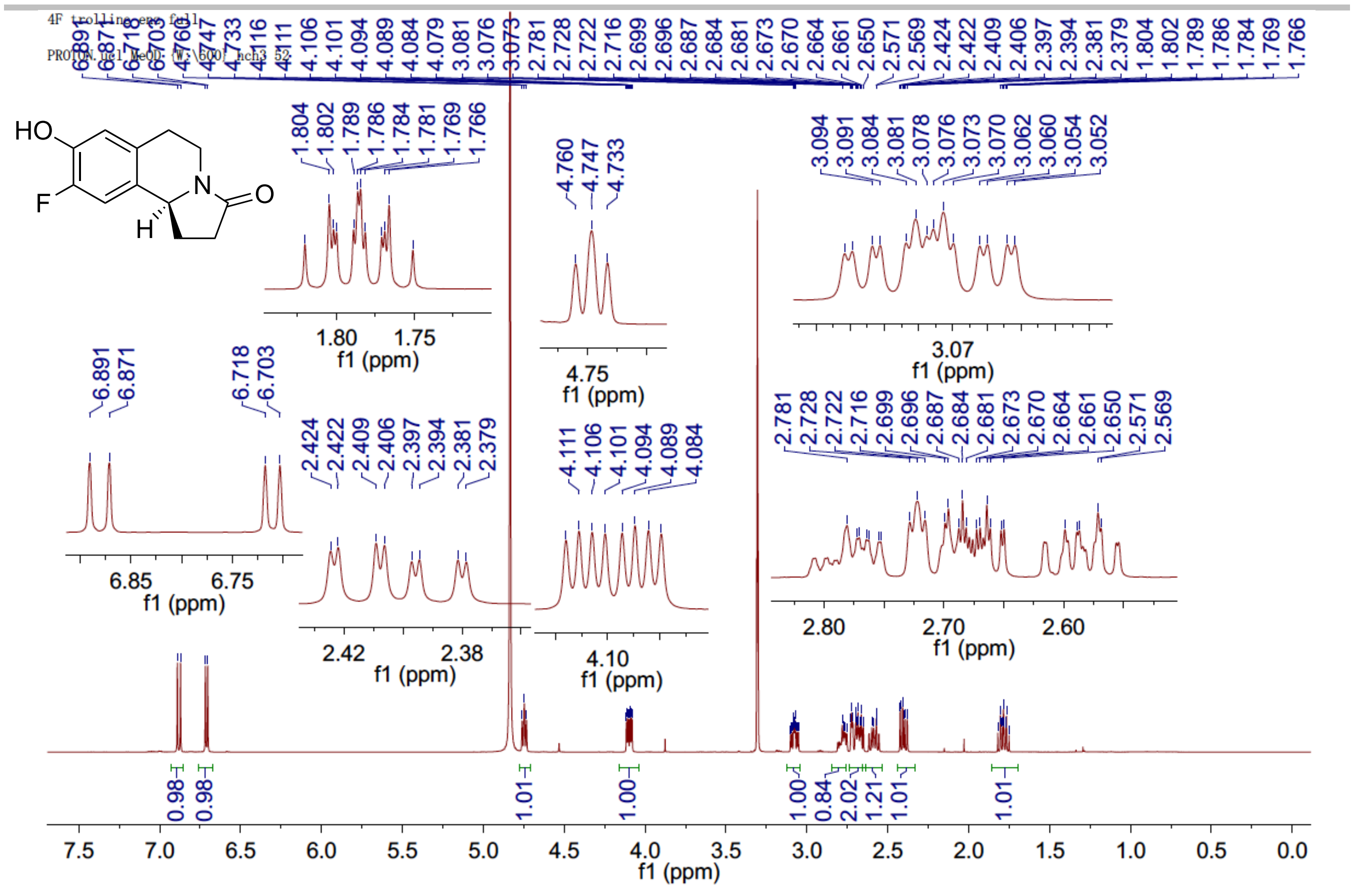

^{1}H NMR spectrum (compound (*S*)-**18**)

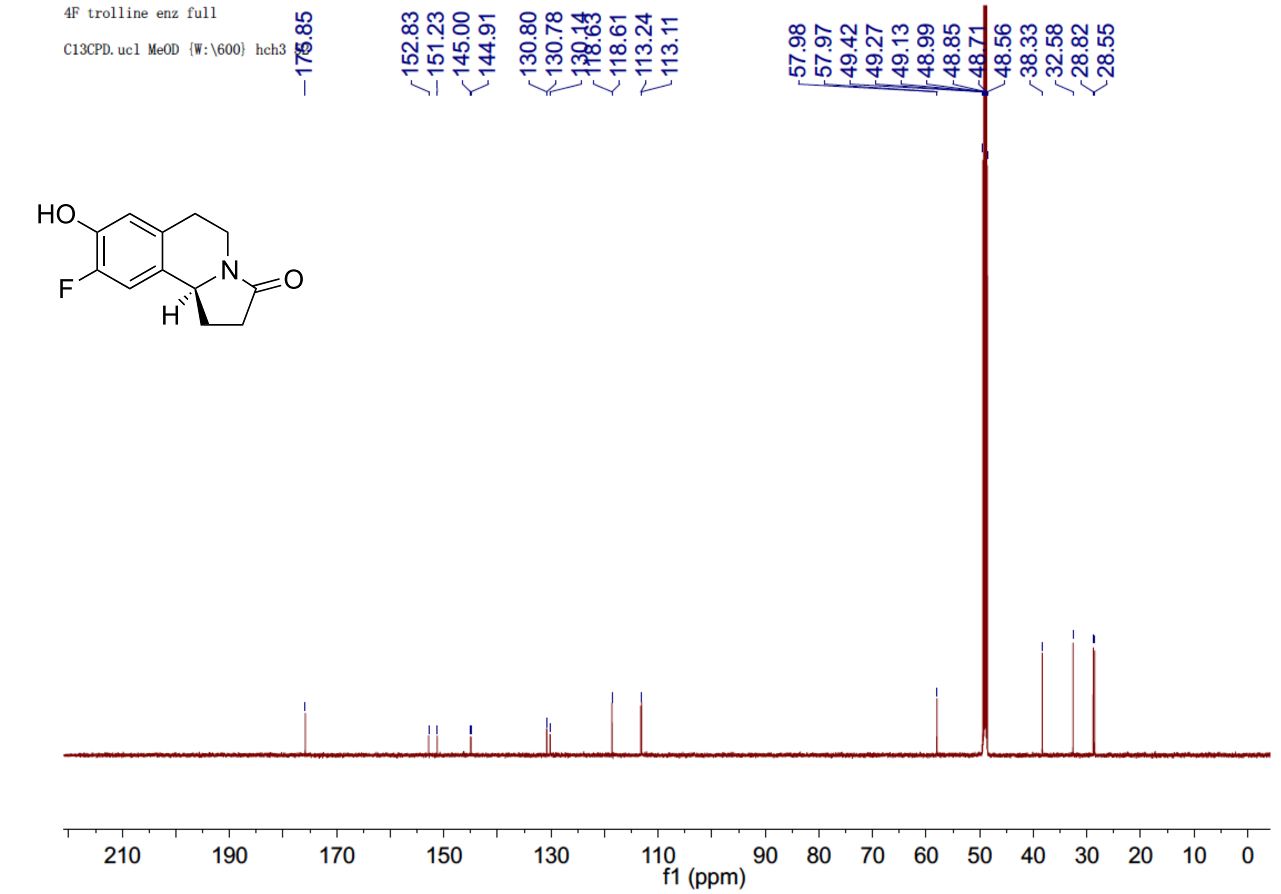

^{13}C NMR spectrum (compound (*S*)-**18**)

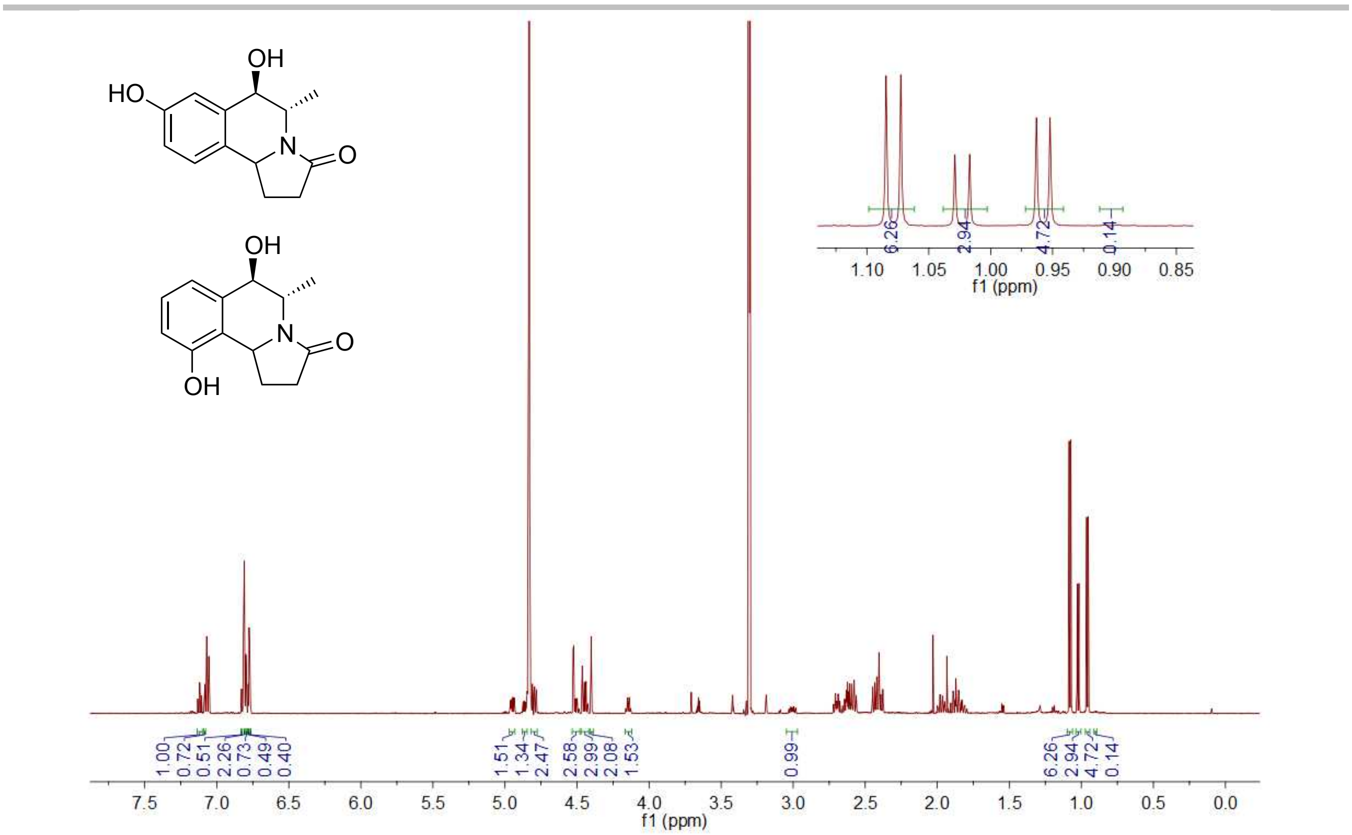

^{1}H NMR spectrum (compound **17** isomers)

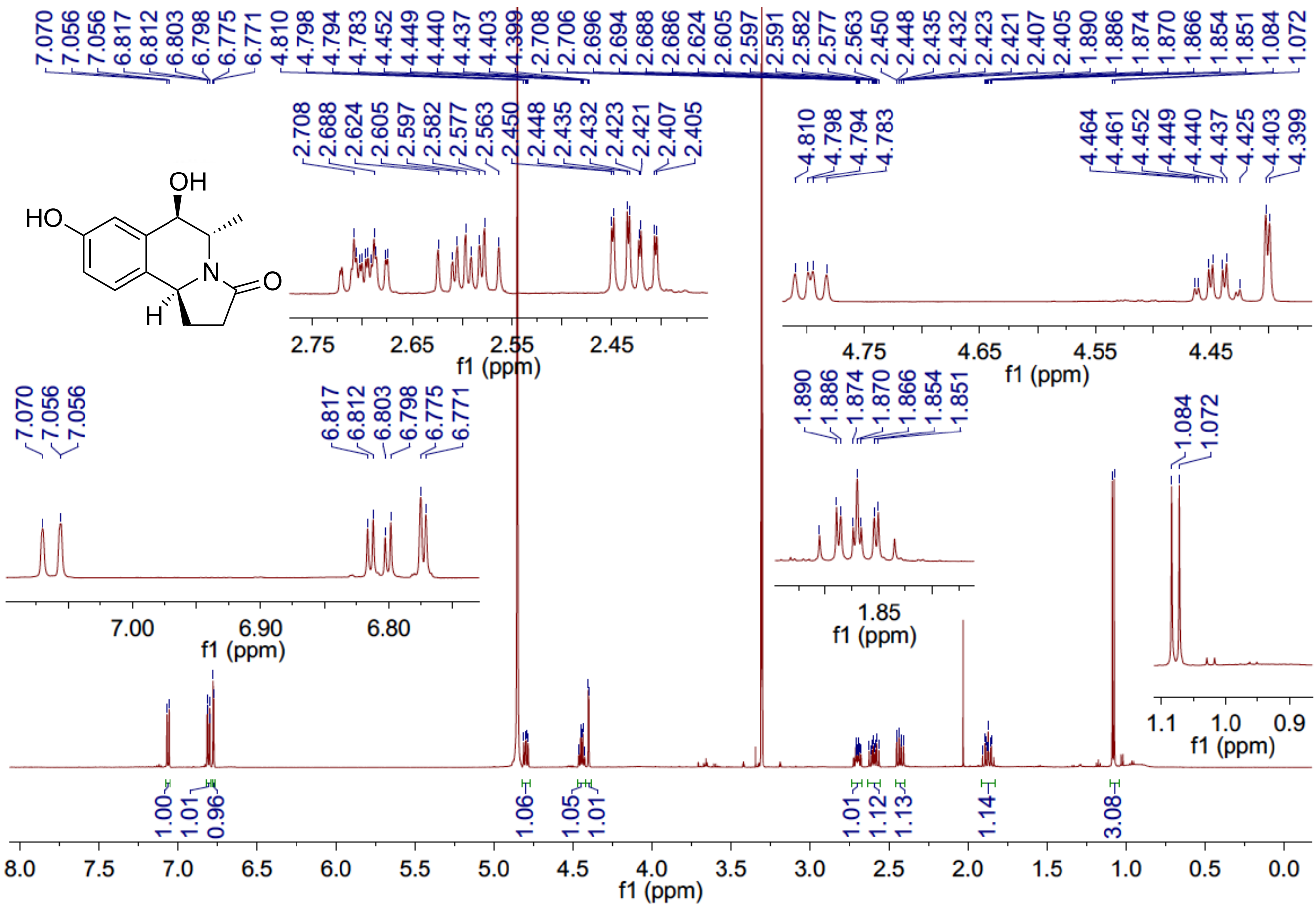

^{1}H NMR spectrum (compound (*S*)-**17**)

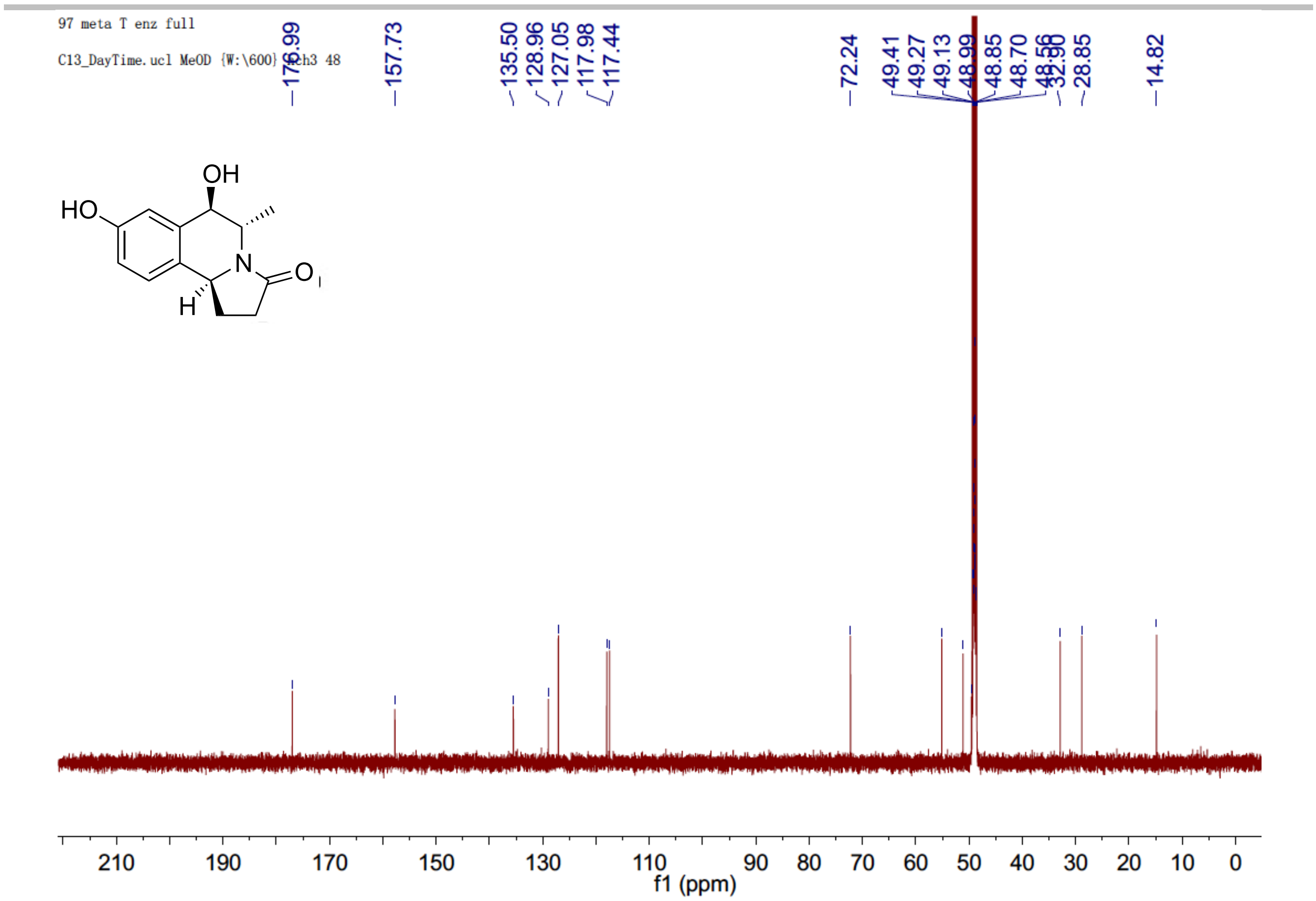

^{13}C NMR spectrum (compound (*S*)-**17**)

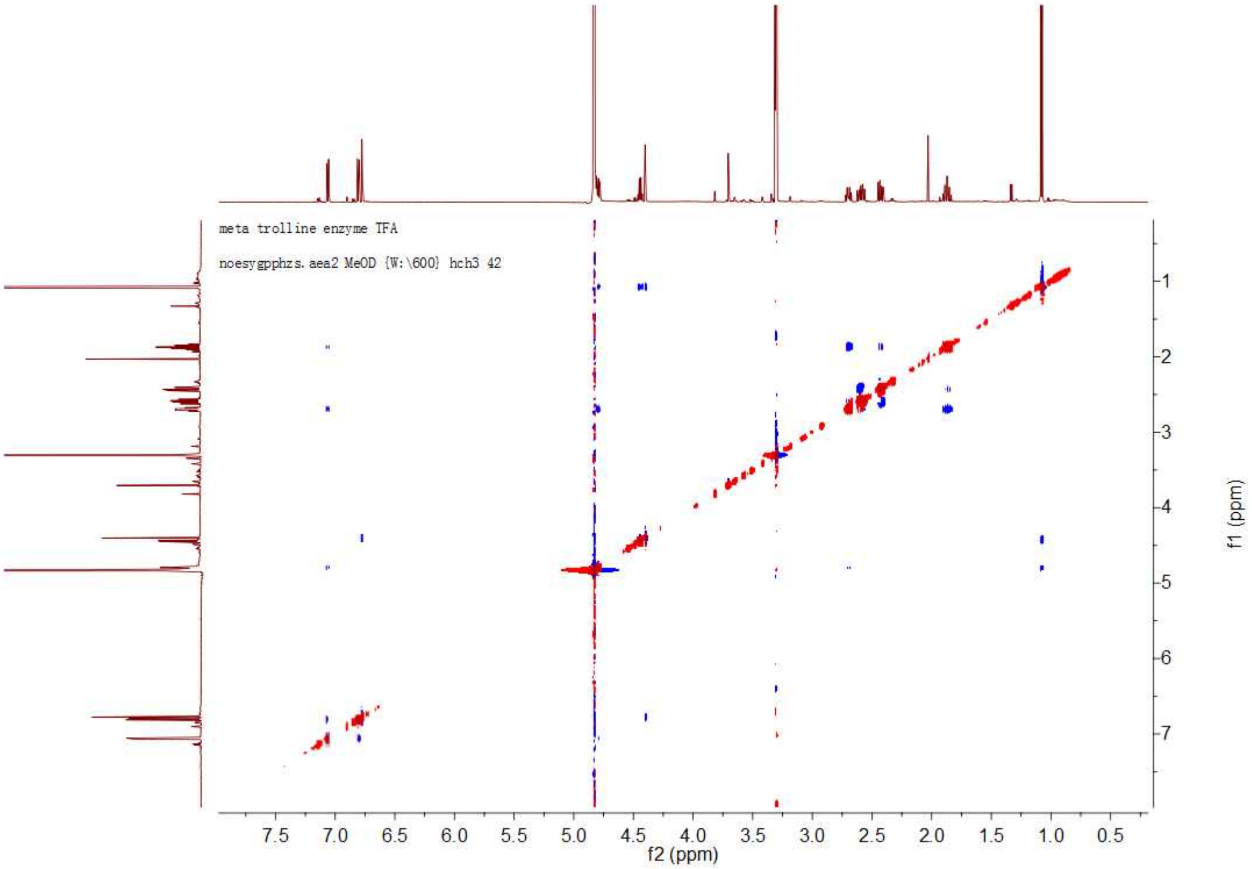

NOESY spectrum (compound (*S*)-**17**)

5 References

1. B. R. Lichman, J. Zhao, H. C. Hailes and J. M. Ward, *Nat. Commun.,* 2017, **8**, 14883.
2. B. R. Lichman, A. Sula, T. Pesnot, H. C. Hailes, J. M. Ward and N. H. Keep, *Biochemistry*, 2017, **56**, 5274.
3. B. R. Lichman, E. D. Lamming, T. Pesnot, J. M. Smith, H. C. Hailes and J. M. Ward, *Green Chem.*, 2015, **17**, 852.
4. O. Trott and A. J. Olson, *J. Comput. Chem.,* 2010, **31**, 455.
5. B. R. Lichman, M. C. Gershater, E. D. Lamming, T. Pesnot, A. Sula, N. H. Keep, H. C. Hailes and J. M. Ward, *FEBS J.,* 2015*,* **282**, 1137.
6. C. Cook, F. Liron, X. Guinchard and E. Roulland, *J. Org. Chem.*, 2012, **77**, 6728.
7. S. E. Choi and M. K. H. Pflum, *Bioorg. Med. Chem. Lett.,* 2012, **22**, 7084.
8. N. Kawai, M. Matsuda and J. Uenishi, *Tetrahedron*, 2011, **67**, 8648.
9. F. Claudi, M. Cardellini, G. M. Cingolani, A. Piergentili, G. Peruzzi and W. Balduini, *J. Med. Chem.,* 1990, **33**, 2408.
